# A multi-centre, phase 1a/1b dose escalation and expansion study of the HER2-directed antibody–drug conjugate T-Bren (BL-M07D1) in advanced breast cancer and other solid tumours

**DOI:** 10.1016/j.ebiom.2026.106351

**Published:** 2026-06-25

**Authors:** Herui Yao, Ruihua Zhao, Xiuping Lai, Qing Wen, Meili Sun, Ying Wang, Suiwen Ye, Jun Jia, Ling Guan, Yongqiang Zhang, Xian Wang, Yahua Zhong, Jianying Huang, Mei Li, Junlan Guo, Fuguo Tian, Yuan Yuan, Sa Xiao, Hai Zhu, Yi Zhu, Rongbo Lin, Hong Zong, Erwei Song

**Affiliations:** a Sun Yat-sen Memorial Hospital, Sun Yat-sen University, Guangzhou, China; b The First Affiliated Hospital of Zhengzhou University, Zhengzhou, China; c Central Hospital Affiliated to Shandong First Medical University, Jinan, China; d The Tenth Affiliated Hospital, Southern Medical University (Dongguan People's Hospital), Dongguan, China; e Department of Medical Oncology, Beijing Hospital, National Center of Gerontology, National Health Commission, Institute of Geriatric Medicine, Chinese Academy of Medical Sciences, Beijing, China; f Sir Run Run Shaw Hospital, School of Medicine, Zhejiang University, Hangzhou, China; g Zhongnan Hospital of Wuhan University, Wuhan, China; h West China Hospital, Sichuan University, Chengdu, China; i Anyang Tumor Hospital, Anyang, China; j Shanxi Cancer Hospital, Taiyuan, China; k Xuzhou Central Hospital, Southeast University, Xuzhou, China; l Baili-Bio (Chengdu) Pharmaceutical, Chengdu, China; m SystImmune, Redmond, WA, USA; n Sichuan Biokin Pharmaceutical, Chengdu, China; o Department of Gastrointestinal Oncology, Clinical Oncology School of Fujian Medical University, Fujian Cancer Hospital, Fuzhou, China

**Keywords:** ErbB receptors, Antibody-drug conjugate, Breast neoplasms, Drug resistance

## Abstract

**Background:**

Trastuzumab brengitecan (T-Bren; BL-M07D1) is a HER2-directed antibody-drug conjugate (ADC) comprising a monoclonal antibody, a cathepsin B–cleavable linker, and a potent topoisomerase I inhibitor payload (Ed-04). We aimed to evaluate the safety, tolerability, pharmacokinetics, and preliminary antitumour activity of T-Bren in patients with advanced breast cancer and other solid tumours.

**Methods:**

This phase 1 dose escalation and expansion study enrolled patients with inoperable locally advanced or metastatic breast cancer or other solid tumours pretreated with systemic therapy. Patients received intravenous T-Bren at doses ranging from 1.0 mg/kg on days 1 and 8 every 3 weeks (D1D8 Q3W) (accelerated titration), to 2.6 mg/kg, 3.2 mg/kg, 3.8 mg/kg, 4.4 mg/kg, 5.0 mg/kg, 5.6 mg/kg, 6.2 mg/kg on day 1 every 3 weeks (D1Q3W) (i3+3 design). Primary objectives were to assess dose-limiting toxicity/maximum tolerated dose (DLT/MTD), and establish the recommended phase 2 dose (RP2D).

**Findings:**

Overall, 253 patients were treated: DLTs occurred in two patients treated at 6.2 mg/kg Q3W, including grade 4 neutropenia with myelosuppression and grade 3 thrombocytopaenia in one patient, and grade 4 febrile neutropenia in another. In patients with breast cancer, ORR was 81.5% (66/81) in HER2-positive subgroup, 69.5% (57/82) in hormone receptor–positive/HER2-low disease, and 58.3% (14/24) in hormone receptor–negative/HER2-low disease. Median PFS was 18.2 months, 14.0 months, and 7.2 months in the HER2-positive, HR-positive/HER2-negative, and HR-negative/HER2-negative subgroups, respectively.

**Interpretation:**

T-Bren demonstrated a manageable safety profile and clinically meaningful antitumour activity across a broad spectrum of HER2 expression in advanced breast cancer. The 4.4 mg/kg every three weeks regimen was established as the RP2D in breast cancer.

**Funding:**

This study was funded by the sponsor Baili-Bio (Chengdu) Pharmaceutical Co., Ltd.

**Research in context**

**Evidence before this study**

We searched PubMed using the search terms “human epidermal growth factor receptor 2 or trastuzumab deruxtecan or trastuzumab emtansine” and “antibody-drug conjugate” and “clinical trial” for clinical trial papers (phase 1–3) on HER2-directed ADCs in solid tumours published from 1 January 2016 to 26 February 2026 reporting primary analysis results, with no language restrictions. In total, 44 papers were identified, of which 10 papers were on phase 3 studies, focussing on trastuzumab deruxtecan. Trastuzumab deruxtecan demonstrated a statistically significant and clinically meaningful benefit in HER2-positive and HER2-low metastatic breast cancer. In addition, in phase 2 studies, trastuzumab deruxtecan showed encouraging antitumour activity in HER2-mutant non-small cell lung cancer, HER2-positive colorectal cancer, and HER2-positive gastric cancer.

**Added value of this study**

This phase 1 study demonstrated a manageable safety profile of T-Bren, a HER2-directed ADC with a potent topoisomerase I inhibitor payload (Ed-04) and a cleavable linker. This study also showed encouraging antitumour activity of T-Bren in patients with HER2-positive and HER2-low breast cancer, as well as other solid tumours (e.g. lung cancer and gastric cancer).

**Implications of all the available evidence**

Findings from this study support further clinical studies of T-Bren in HER2-expressing breast cancer and other solid tumours.

## Introduction

Human epidermal growth factor receptor 2 (HER2), is a member of the epidermal growth factor receptor (EGFR) family of receptor tyrosine kinases and plays a central role in regulating cellular proliferation, differentiation, and survival.^1^ Lacking a known soluble ligand, HER2 signals primarily through heterodimerisation with other EGFR family members such as HER1 and HER3, leading to activation of oncogenic downstream pathways.^2^ Aberrant HER2 activation has been established as a key oncogenic driver in multiple solid tumours, including breast cancer.^3^ HER2 overexpression or amplification is observed across multiple solid tumour types, including urothelial cancer (35.8%),^4^ gastric cancer (22.1%),^5^ and breast cancer (13%),^6^ supporting HER2 as a validated therapeutic target. In breast cancer, HER2-targeted therapies have transformed clinical outcomes for patients with HER2-positive disease, establishing HER2-directed treatment as a cornerstone of modern oncology practice. Nevertheless, despite substantial advances, disease progression remains inevitable for many patients, particularly in later-line settings, highlighting persistent unmet clinical needs.^7^, ^8^, ^9^ Clinically, HER2 expression in breast cancer has traditionally been dichotomised based on immunohistochemistry (IHC) and in situ hybridisation (ISH) into HER2-positive and HER2-negative categories.^10^ HER2-positive disease is defined as IHC 3+ or IHC 2+ with HER2 gene amplification, whereas HER2-negative disease includes tumours with IHC 0, 1+, or IHC 2+ without amplification.^10^ This binary classification has guided treatment selection for more than two decades. However, emerging evidence suggests that HER2 expression exists along a biological continuum rather than a strict binary state, raising important questions about the therapeutic relevance of lower levels of HER2 expression.

Patients with advanced HER2-positive breast cancer derive substantial benefit from first-line dual HER2 blockade combined with chemotherapy.^7^^,^^11^ Yet, treatment efficacy diminishes with successive lines of therapy, and central nervous system involvement becomes increasingly common, underscoring the need for more effective and durable HER2-directed strategies.^12^^,^^13^

In parallel, patients with hormone receptor–positive/HER2-negative breast cancer and triple-negative breast cancer (TNBC)—which together account for the majority of breast cancer cases—face limited treatment options in later lines.^14^^,^^15^ Endocrine therapy combined with CDK4/6 inhibitors yields incomplete and often transient responses in hormone receptor–positive disease, while TNBC is characterised by aggressive clinical behaviour and poor survival after chemotherapy failure.^16^ These challenges highlight a substantial unmet need for novel therapeutic approaches that can extend benefit beyond conventional HER2-positive populations.

Antibody-drug conjugates (ADCs) have emerged as a promising therapeutic modality capable of bridging target specificity with potent cytotoxic activity.^17^ By coupling a monoclonal antibody to a cytotoxic payload through a cleavable linker, ADCs enable selective delivery of chemotherapy to tumour cells expressing the target antigen, as well as adjacent tumour cells with low levels of target expression, which can be caused by cytotoxic by-stander effect of the payloads, thereby expanding its potentiality for overcoming tumour heterogeneity.^18^

Trastuzumab brengitecan (T-Bren, also known as BL-M07D1), is a HER2-directed antibody–drug conjugate composed of an anti-HER2 monoclonal antibody linked via a cathepsin B–cleavable linker to a potent topoisomerase I inhibitor payload (Ed-04), with a drug-to-antibody ratio of eight.^19^ In preclinical studies, T-Bren demonstrated robust antitumour activity in xenograft models with both high and low HER2 expression, supporting its potential to address the HER2 expression continuum. On the basis of these findings, we conducted a phase 1 dose escalation and expansion study to evaluate the safety, tolerability, pharmacokinetics and preliminary antitumour activity of T-Bren in patients with locally advanced or metastatic breast cancer and other solid tumours.

## Methods

### Study design and patients

This was a phase 1, multicentre, open-label, non-randomised, dose-escalation (phase 1a) and expansion study (phase 1b) conducted in China. Study design schema is presented in the Supplementary Material. Eligible patients were with histologically or cytologically confirmed, inoperable locally advanced or metastatic breast cancer and other solid tumours; failed, lacked access to, or were not suitable for standard treatment; and HER2 status positive (defined as IHC 3+, or IHC 2+ and ISH +) or HER2-negative (including HER2-low and HER2 IHC 0; HER2-low was defined as IHC 2+ and ISH −, or IHC 1+). Baseline ER, PR, and HER2 expression status was determined according to patients' medical records except for those with HER2 IHC0, which was reassessed by local hospitals at study entry. Other inclusion criteria included aged ≥18 years old (18–75 years for dose-escalation part); Eastern Cooperative Oncology Group (ECOG) performance status of 0 or 1; left ventricular ejection fraction (LVEF) of at least 50%; at least one measurable lesion per Response Evaluation Criteria in Solid Tumours (RECIST) version 1.1; toxicities of previous anticancer therapy had recovered to grade ≤1 per National Cancer Institute-Common Terminology Criteria for Adverse Events (NCI-CTCAE) version 5.0; and adequate organ function within 14 days before first dose of study drug.

Key exclusion criteria included prior treatment with a topoisomerase I inhibitor-based ADC (for expansion part only); a history of clinically significant cardiac disease; QT interval prolongation of more than 450 ms in men or more than 470 ms in women, complete left bundle branch block or third-degree atrioventricular block; grade ≥3 lung disease, or grade ≥2 radiation-induced lung disease per NCI-CTCAE version 5.0, or with history of interstitial lung disease (ILD) or ILD at screening; active autoimmune and inflammatory disease; unstable thromboembolic events that required therapeutic intervention within 6 months before screening; and previously treated with anthracyclines at a cumulative dose >360 mg/m^2^.

### Ethics

The study protocols, amendments, and informed consent forms were approved by the ethics committee at the leading study hospital Sun Yat-sen Memorial Hospital, Sun Yat-sen University (reference number: SYSYW-2022-092-01), as well as ethics committees at each participating study hospitals (Supplementary Table S1). This study was conducted in accordance with the Declaration of Helsinki, the International Council for Harmonisation E6 Guideline for Good Clinical Practice, and local regulations. All patients provided written informed consent before start of any study procedure. This study was approved by the China National Medical Products Administration (reference number: CXSL2200209) and is registered at Clinicaltrials.gov (NCT05461768).

### Study treatment

During dose-escalation part, an accelerated titration combined with interval 3+3 (i3+3) design was used. At each dose level, two to four patients (one patient at starting dose level for accelerated titration) were to complete the dose-limiting toxicity (DLT) assessment before moving to the next dose level. Detailed rules are presented in Supplementary Material. Dose escalation with the i3+3 design was guided by using a Bayesian framework and Beta-Bernoulli model. For the i3+3 design, a target DLT rate of 28% with equivalence interval of 23%–33% (target DLT rate ±5%) was adopted.

In dose-escalation part, dose levels of 1.0 mg/kg D1D8 Q3W, 2.6, 3.2, 3.8, 4.4, 5.0, 5.6, 6.2, 6.8, and 7.4 mg/kg D1 Q3W were planned and detailed rationale for planned dose levels is presented in Supplementary Material. During dose-escalation phase, two out of five patients at 6.2 mg/kg D1 Q3W dose level experienced three DLT events. According to i3+3 rules, no additional patient was enrolled and dose escalation was stopped at 6.2 mg/kg D1 Q3W dose level. No DLT was observed at dose levels 3.8, 4.4, 5.0, and 5.6 mg/kg D1 Q3W and these dose levels were selected for expansion part.

Treatment was continued until disease progression, intolerable toxicity, start of new anticancer therapy, or other discontinuation events. Dose interruptions due to treatment toxicities were permitted. If the subsequent dose was delayed for more than 28 days, the patient was discontinued from the study. A total of two dose reductions were allowed after the DLT observation period (the first treatment cycle after drug administration at each dose level). Once dose was reduced, further escalation was not allowed.

### Study assessments

Tumour assessments were performed by using enhanced CT or MRI at baseline, every 6 weeks through the end of first year of treatment, and every 12 weeks during the second year of treatment (treatment could be continued beyond the second year of treatment until discontinuation criteria was met). After end of treatment, tumour assessments were performed every 12 weeks during the first year and every 24 weeks during the second year and afterwards. Tumour response was assessed by investigators per RECIST version 1.1. Adverse events (AEs) were graded according to the National Cancer Institute Common Terminology Criteria for Adverse Events (version 5.0). Baseline sex data were self-reported by patients.

### Determination of maximum tolerated dose

Dose-limiting toxicities (DLTs) were observed during the first treatment cycle after drug administration at each dose level. When DLT observation period was completed, safety review was conducted by the investigator and sponsor and the decision on dose escalation or dose expansion was made. After completion of dose-escalation part, the maximum tolerated dose (MTD) was determined according to assessments of the DLT rates at each dose level, which were estimated by using the pool-adjacent-violator algorithm and isotonic regression.

### Study outcomes

Primary outcomes for dose-escalation part of this study were DLT and MTD. The primary outcome for expansion part was recommended phase 2 dose (RP2D). Secondary outcomes for this phase 1 study included safety and efficacy. Efficacy outcomes included objective response rate (ORR), defined as the proportion of patients who achieved a complete response (CR) or partial response (PR) as best response; disease control rate (DCR), defined as the proportion of patients with CR, PR, or stable disease (SD) as best response; clinical benefit rate (CBR), defined as the proportion of patients who achieved a CR, PR, or SD (lasting for at least 6 months) as best response; time to response (TTR), defined as the time from the first dose to the first documented response (confirmed CR or PR) in patients who achieved confirmed CR or PR; duration of response (DoR), defined as the time from first documented CR or PR to progressive disease (PD) or death in patients with confirmed CR or PR, whichever occurred first; PFS, defined as the time from first dose of study treatment to first documented PD or death, whichever occurred first; OS, defined as the time from first dose of study treatment to death.

### Statistics

Safety set (SS) included patients who received at least one dose of T-Bren. Modified intention-to-treat population (mITT) included patients who received at least one dose of T-Bren, excluding those who still on treatment and did not reach the timepoint for the first tumour assessment. Safety outcomes were analysed in SS. Efficacy outcomes were analysed in mITT. Plasma drug concentration was analysed in patients who received at least one dose of T-Bren and had at least one evaluable plasma drug concentration. Pharmacokinetic parameter was analysed in patients who received at least one dose of T-Bren and had at least one computable pharmacokinetic parameter.

Kaplan–Meier method was used to estimate DoR, PFS, and OS and corresponding 95% confidence intervals (CIs) were determined by using the Brookmeyer-Crowley method. The 95% CIs for survival rates were calculated by using the Greenwood's formula. The 95% CIs for ORR, DCR and CBR were calculated using the Clopper–Pearson exact method. All statistical analyses were performed by using SAS Software version 9.4 (Cary, NC). The pharmacokinetic parameters of T-Bren and the released payload were estimated using a noncompartmental model by Phoenix WinNonlin software (Certara, Princeton, NJ), version 8.5.2.

There was no statistical hypothesis specified for this study. For dose-escalation phase, accelerated titration was used for the first dose level and i3+3 design was used for the subsequent nine dose levels. A total of about 28 patients (one in accelerated titration cohort and three in each of dose-escalation cohorts) were needed for observation of DLT. The maximum sample size was determined as 55 according to traditional 3+3 design (one in accelerated titration cohort and six in each of dose-escalation cohorts). Dose levels of 3.8, 4.4, 5.0, and 5.6 mg/kg D1 Q3W were selected for expansion part. The sample size in each of expansion cohorts was adjusted according to accumulated safety and efficacy data, aiming at further assessment of safety and preliminary efficacy in certain tumour types.

### Role of funders

The study was funded by the sponsor Baili-Bio (Chengdu) Pharmaceutical Co., Ltd, which was involved in study design, data collection, data analysis, data interpretation, review and approval of the final version of the manuscript for publication.

## Results

### Patients

**Fig. 1:**
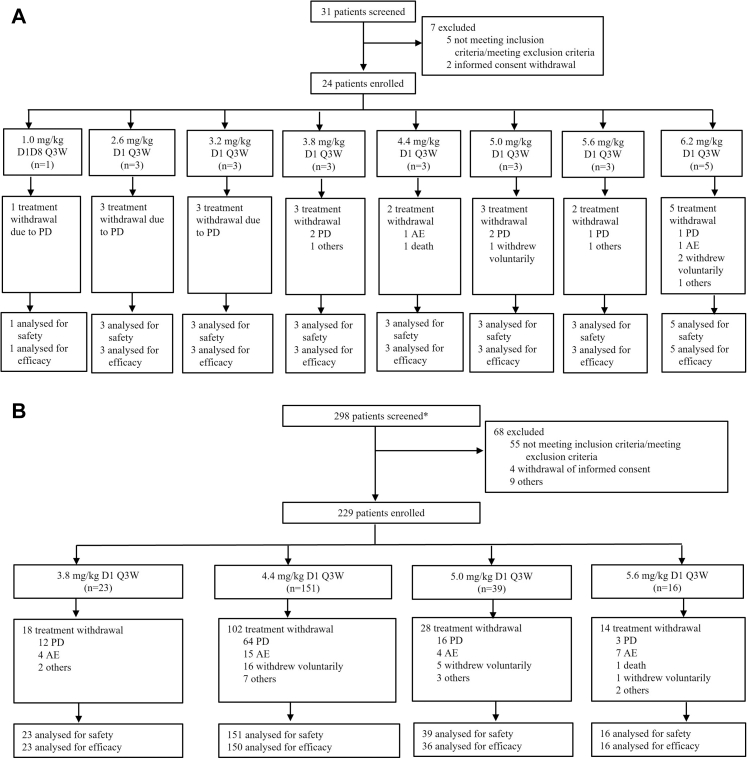
Patient disposition. A. Patient disposition in phase 1a study. B. Patient disposition in phase 1b study. ∗One patient was under screening at data cutoff date (31 July 2025). AE, adverse event; D, day; PD, progressive disease; Q3W, once every three weeks.

Patient disposition is presented in Fig. 1. Between 9 Aug 2022 and 17 July 2025, 253 patients with inoperable locally advanced or metastatic breast cancer or other solid tumours were enrolled across 12 centres in China (Supplementary Table S1) and received T-Bren. Of these, 24 patients were treated in the dose-escalation part (Fig. 1A) and 229 in expansion part (Fig. 1B). The data cutoff for this analysis was 31 July 2025. At data cutoff, two patients in the dose-escalation part and 67 patients in expansion part remained on study treatment. Disease progression was the most common reason for treatment discontinuation.

Baseline demographics and disease characteristics are summarised in Tables 1 and 2. In dose-escalation part, the median age of all patients was 53 years (IQR 43.5–58.5) (Table 1). Most patients had an ECOG performance status of 1 (17 [70.8%]). Patients were heavily pretreated, with 12 (50.0%) have received three or more prior lines of systemic therapy. Breast cancer accounted for 18 patients (75.0%). In expansion part, the median age of all patients was 55 years (IQR 48–61) and 195 (85.2%) patients had an ECOG performance status of 1 (Table 2). Median prior line of therapy was 2 (IQR 2–4). Most patients had breast cancer (n = 181, 79%). In addition, patients with lung cancer (n = 13, 5.7%), gastric cancer (n = 18, 7.9%), and colorectal cancer (n = 17, 7.4%) were enrolled.

**Table 1:** Patient demographics and baseline characteristics for Phase 1a (safety population).

Characteristics	Total (N = 24)	T-Bren D1D8 Q3W	T-Bren D1 Q3W						
		1.0 mg/kg (N = 1)	2.6 mg/kg (N = 3)	3.2 mg/kg (N = 3)	3.8 mg/kg (N = 3)	4.4 mg/kg (N = 3)	5.0 mg/kg (N = 3)	5.6 mg/kg (N = 3)	6.2 mg/kg (N = 5)
Age, years, median (Q1, Q3)	53.0 (43.5, 58.5)	53.0 (53.0, 53.0)	52.0 (46.0, 56.0)	55.0 (39.0, 62.0)	57.0 (43.0, 65.0)	56.0 (44.0, 67.0)	50.0 (47.0, 67.0)	56.0 (38.0, 60.0)	42.0 (40.0, 53.0)
Sex, n (%)									
Male	2 (8.3)	0	0	0	0	0	1 (33.3)	0	1 (20.0)
Female	22 (91.7)	1 (100)	3 (100)	3 (100)	3 (100)	3 (100)	2 (66.7)	3 (100)	4 (80.0)
Race, n (%)									
Asian	24 (100.0)	1 (100.0)	3 (100.0)	3 (100.0)	3 (100.0)	3 (100.0)	3 (100.0)	3 (100.0)	5 (100.0)
ECOG performance status, n (%)									
0	7 (29.2)	0	1 (33.3)	1 (33.3)	1 (33.3)	1 (33.3)	2 (66.7)	0	1 (20.0)
1	17 (70.8)	1 (100)	2 (66.7)	2 (66.7)	2 (66.7)	2 (66.7)	1 (33.3)	3 (100)	4 (80.0)
BMI, kg/m^2^, mean (SD)	22.9 (3.3)	20.7 (−)	20.1 (3.0)	23.8 (4.5)	24.3 (5.5)	24.8 (1.6)	21.2 (0.7)	25.5 (1.3)	21.7 (3.2)
Prior lines of therapy, median (Q1, Q3)	2.5 (2.0, 4.5)	2.0 (2.0, 2.0)	2.0 (2.0, 13.0)	3.0 (2.0, 6.0)	2.0 (2.0, 6.0)	3.0 (1.0, 6.0)	2.0 (2.0, 2.0)	5.0 (3.0, 8.0)	3.0 (2.0, 3.0)
Prior lines of therapy, n (%)									
1	1 (4.2)	0	0	0	0	1 (33.3)	0	0	0
2	11 (45.8)	1 (100)	2 (66.7)	1 (33.3)	2 (66.7)	0	3 (100)	0	2 (40.0)
≥3	12 (50.0)	0	1 (33.3)	2 (66.7)	1 (33.3)	2 (66.7)	0	3 (100)	3 (60.0)
Cancer types, n (%)									
Breast cancer	18 (75.0)	1 (100)	3 (100)	2 (66.7)	3 (100)	3 (100)	1 (33.3)	3 (100)	2 (40.0)
HER2-positive^a^	12 (50.0)	1 (100)	2 (66.7)	1 (33.3)	3 (100)	3 (100)	0	1 (33.3)	1 (20.0)
HER2-negative (HER2-low and HER2 0)	6 (25.0)	0	1 (33.3)	1 (33.3)	0	0	1 (33.3)	2 (66.7)	1 (20.0)
Gastric cancer	3 (12.5)	0	0	0	0	0	2 (66.7)	0	1 (20.0)
Colorectal cancer	2 (8.3)	0	0	0	0	0	0	0	2 (40.0)
Others	1 (4.2)	0	0	1 (33.3)	0	0	0	0	0
HER2 status, n (%)									
IHC 1+	4 (16.7)	0	0	1 (33.3)	0	0	1 (33.3)	0	2 (40.0)
IHC 2+ and ISH−	3 (12.5)	0	1 (33.3)	0	0	0	0	2 (66.7)	0
IHC 2+ and ISH+	2 (8.3)	0	0	0	1 (33.3)	1 (33.3)	0	0	0
IHC 2+^b^	3 (12.5)	0	0	0	0	0	1 (33.3)	0	2 (40.0)
IHC 3+	12 (50.0)	1 (100)	2 (66.7)	2 (66.7)	2 (66.7)	2 (66.7)	1 (33.3)	1 (33.3)	1 (20.0)
Number of metastatic sites, n (%)									
1–2	9 (37.5)	0	2 (66.7)	0	0	2 (66.7)	2 (66.7)	2 (66.7)	1 (20.0)
≥3	15 (62.5)	1 (100)	1 (33.3)	3 (100)	3 (100)	1 (33.3)	1 (33.3)	1 (33.3)	4 (80.0)
Baseline lung metastases, n (%)	10 (41.7)	1 (100)	1 (33.3)	1 (33.3)	2 (66.7)	2 (66.7)	1 (33.3)	0	2 (40.0)
Baseline liver metastases, n (%)	10 (41.7)	0	2 (66.7)	3 (100)	1 (33.3)	0	1 (33.3)	2 (66.7)	1 (20.0)
Baseline bone metastases, n (%)	11 (45.8)	0	1 (33.3)	2 (66.7)	2 (66.7)	2 (66.7)	2 (66.7)	0	2 (40.0)
Baseline brain metastases, n (%)	1 (4.2)	0	0	0	0	1 (33.3)	0	0	0
Baseline SOD, mm, median (Q1, Q3)	40.0 (32.6, 97.0)	28.0 (28.0, 28.0)	40.0 (34.3, 40.0)	72.2 (34.7, 111.4)	32.0 (20.0, 34.7)	96.7 (31.0, 223.6)	49.2 (33.2, 97.2)	23.6 (20.5, 52.0)	144.0 (54.7, 149.0)

BMI, body mass index; D, day; ECOG, Eastern Cooperative Oncology Group; Q3W, every 3 weeks; SOD, sum of diameter.

a HER2-positive: HER2 IHC 3+ or IHC 2+/ISH+. HER2-low: HER2 IHC2+/ISH−, or IHC1+. HER2 0: IHC0.

b ISH results were missing.

**Table 2:** Patient demographics and baseline characteristics for Phase 1b (safety population).

Characteristics	Total (N = 229)	T-Bren D1 Q3W			
		3.8 mg/kg (N = 23)	4.4 mg/kg (N = 151)	5.0 mg/kg (N = 39)	5.6 mg/kg (N = 16)
Age, years, median (Q1, Q3)	55.0 (48.0, 61.0)	53.0 (48.0, 60.0)	55.0 (48.0, 61.0)	54.0 (48.0, 63.0)	61.5 (51.0, 67.0)
Sex, n (%)					
Male	31 (13.5)	0	12 (7.9)	13 (33.3)	6 (37.5)
Female	198 (86.5)	23 (100)	139 (92.1)	26 (66.7)	10 (62.5)
Race, n (%)					
Asian	229 (100)	23 (100)	151 (100)	39 (100)	16 (100)
ECOG performance status, n (%)					
0	34 (14.8)	3 (13.0)	24 (15.9)	7 (17.9)	0
1	195 (85.2)	20 (87.0)	127 (84.1)	32 (82.1)	16 (100)
BMI, kg/m^2^, mean (SD)	23.6 (3.7)	24.7 (4.1)	23.6 (3.4)	22.9 (4.5)	22.9 (3.6)
Prior lines of therapy, median (Q1, Q3)	2.0 (2.0, 4.0)	4.0 (2.0, 6.0)	2.0 (2.0, 4.0)	2.0 (1.0, 4.0)	3.0 (2.0, 3.0)
Prior lines of therapy, n (%)					
1	54 (23.6)	5 (21.7)	32 (21.2)	14 (35.9)	3 (18.8)
2	65 (28.4)	3 (13.0)	49 (32.5)	9 (23.1)	4 (25.0)
≥3	110 (48.0)	15 (65.2)	70 (46.4)	16 (41.0)	9 (56.3)
Cancer types, n (%)					
Breast cancer	181 (79.0)	23 (100)	134 (88.7)	21 (53.8)	3 (18.8)
HER2-positive^a^	69 (30.1)	12 (52.2)	43 (28.5)	12 (30.8)	2 (12.5)
HER2-negative (HER2-low and HER2 0)	110 (48.0)	9 (39.1)	91 (60.3)	9 (23.1)	1 (6.3)
Lung cancer	13 (5.7)	0	10 (6.6)	1 (2.6)	2 (12.5)
Gastric cancer	18 (7.9)	0	2 (1.3)	15 (38.5)	1 (6.3)
Colorectal cancer	17 (7.4)	0	5 (3.3)	2 (5.1)	10 (62.5)
HER2 status, n (%)					
IHC 0	12 (5.2)	0	10 (6.6)	2 (5.1)	0
IHC 1+	45 (19.7)	5 (21.7)	36 (23.8)	4 (10.3)	0
IHC 2+ and ISH−	56 (24.5)	4 (17.4)	46 (30.5)	5 (12.8)	1 (6.3)
IHC 2+ and ISH+	24 (10.5)	3 (13.0)	10 (6.6)	6 (15.4)	5 (31.3)
IHC 2+^b^	3 (1.3)	2 (8.7)	1 (0.7)	0	0
IHC 3+	79 (34.5)	9 (39.1)	41 (27.2)	21 (53.8)	8 (50.0)
HER2 mutation	10 (4.4)	0	7 (4.6)	1 (2.6)	2 (12.5)
Number of metastatic sites, n (%)					
0	1 (0.4)	0	0	0	1 (6.3)
1–2	86 (37.6)	8 (34.8)	54 (35.8)	19 (48.7)	5 (31.3)
≥3	142 (62.0)	15 (65.2)	97 (64.2)	20 (51.3)	10 (62.5)
Baseline lung metastases, n (%)	119 (52.0)	14 (60.9)	81 (53.6)	15 (38.5)	9 (56.3)
Baseline liver metastases, n (%)	115 (50.2)	11 (47.8)	78 (51.7)	18 (46.2)	8 (50.0)
Baseline bone metastases, n (%)	113 (49.3)	12 (52.2)	83 (55.0)	15 (38.5)	3 (18.8)
Baseline brain metastases, n (%)	20 (8.7)	3 (13.0)	16 (10.6)	0	1 (6.3)
Baseline SOD, mm, median (Q1, Q3)	49.9 (31.5, 85.2)	62.2 (37.8, 85.1)	49.2 (28.3, 86.0)	46.6 (25.6, 82.0)	56.8 (41.6, 90.2)

BMI, body mass index; D, day; ECOG, Eastern Cooperative Oncology Group; Q3W, every 3 weeks; SOD, sum of diameter.

a HER2-positive: HER2 IHC 3+ or IHC 2+/ISH+. HER2-low: HER2 IHC2+/ISH−, or IHC1+. HER2 0: IHC0.

b ISH results were missing.

Baseline characteristics for patients with breast cancer and other solid tumours are presented in the appendix (Supplementary Tables S2–S4).

### Safety

Safety summary is presented in Supplementary Table S5. Treatment-related adverse events (TRAEs) are summarised in Tables 3 and 4. The most common TRAEs in dose-escalation (Table 3) and expansion parts (Table 4) were haematological toxicities, including anaemia, leukopenia, neutropenia, and thrombocytopaenia.

**Table 3:** Treatment-related adverse events, serious adverse events, and treatment-related adverse events leading to death for phase 1a (safety population).

	Total (N = 24)	T-Bren D1D8 Q3W	T-Bren D1 Q3W						
		1.0 mg/kg (N = 1)	2.6 mg/kg (N = 3)	3.2 mg/kg (N = 3)	3.8 mg/kg (N = 3)	4.4 mg/kg (N = 3)	5.0 mg/kg (N = 3)	5.6 mg/kg (N = 3)	6.2 mg/kg (N = 5)
**Any treatment-related adverse event, n (%)**	**24 (100)**	**1 (100)**	**3 (100)**	**3 (100)**	**3 (100)**	**3 (100)**	**3 (100)**	**3 (100)**	**5 (100)**
AE in ≥10% of total patients									
Leukopenia	22 (91.7)	1 (100)	3 (100)	1 (33.3)	3 (100)	3 (100)	3 (100)	3 (100)	5 (100)
Anaemia	20 (83.3)	1 (100)	3 (100)	1 (33.3)	2 (66.7)	3 (100)	2 (66.7)	3 (100)	5 (100)
Neutropenia	20 (83.3)	1 (100)	3 (100)	1 (33.3)	3 (100)	2 (66.7)	3 (100)	3 (100)	4 (80.0)
Thrombocytopaenia	14 (58.3)	0	1 (33.3)	1 (33.3)	2 (66.7)	1 (33.3)	2 (66.7)	3 (100)	4 (80.0)
Nausea	13 (54.2)	1 (100)	3 (100)	2 (66.7)	0	2 (66.7)	0	2 (66.7)	3 (60.0)
Asthenia	11 (45.8)	1 (100)	2 (66.7)	0	0	2 (66.7)	2 (66.7)	1 (33.3)	3 (60.0)
Stomatitis	11 (45.8)	1 (100)	1 (33.3)	1 (33.3)	1 (33.3)	2 (66.7)	2 (66.7)	1 (33.3)	2 (40.0)
Vomiting	11 (45.8)	1 (100)	2 (66.7)	1 (33.3)	1 (33.3)	3 (100)	1 (33.3)	0	2 (40.0)
Decreased appetite	9 (37.5)	1 (100)	1 (33.3)	1 (33.3)	1 (33.3)	1 (33.3)	1 (33.3)	1 (33.3)	2 (40.0)
Hypokalaemia	9 (37.5)	0	3 (100)	1 (33.3)	0	2 (66.7)	0	0	3 (60.0)
Lymphocyte count decreased	8 (33.3)	1 (100)	1 (33.3)	1 (33.3)	1 (33.3)	0	0	1 (33.3)	3 (60.0)
Weight decreased	8 (33.3)	1 (100)	0	0	1 (33.3)	1 (33.3)	2 (66.7)	1 (33.3)	2 (40.0)
Alopecia	7 (29.2)	0	0	0	1 (33.3)	2 (66.7)	1 (33.3)	1 (33.3)	2 (40.0)
Aspartate aminotransferase increased	7 (29.2)	0	1 (33.3)	0	2 (66.7)	1 (33.3)	0	2 (66.7)	1 (20.0)
Gamma-glutamyltransferase increased	7 (29.2)	1 (100)	3 (100)	1 (33.3)	0	1 (33.3)	0	1 (33.3)	0
Occult blood positive	7 (29.2)	1 (100)	2 (66.7)	2 (66.7)	1 (33.3)	1 (33.3)	0	0	0
Alanine aminotransferase increased	6 (25.0)	0	1 (33.3)	0	0	1 (33.3)	0	1 (33.3)	3 (60.0)
Blood alkaline phosphatase increased	6 (25.0)	1 (100)	2 (66.7)	0	1 (33.3)	1 (33.3)	0	1 (33.3)	0
Dizziness	6 (25.0)	0	0	0	2 (66.7)	1 (33.3)	1 (33.3)	1 (33.3)	1 (20.0)
Urinary tract infection	6 (25.0)	1 (100)	1 (33.3)	0	1 (33.3)	1 (33.3)	0	0	2 (40.0)
Blood lactate dehydrogenase increased	5 (20.8)	0	2 (66.7)	1 (33.3)	0	1 (33.3)	0	1 (33.3)	0
Electrocardiogram T wave abnormal	5 (20.8)	1 (100)	1 (33.3)	1 (33.3)	1 (33.3)	0	0	1 (33.3)	0
Headache	5 (20.8)	0	1 (33.3)	0	1 (33.3)	1 (33.3)	0	1 (33.3)	1 (20.0)
Hypocalcaemia	5 (20.8)	1 (100)	1 (33.3)	1 (33.3)	0	0	0	0	2 (40.0)
Peripheral sensory neuropathy	5 (20.8)	1 (100)	1 (33.3)	1 (33.3)	0	1 (33.3)	0	0	1 (20.0)
Electrocardiogram QT prolonged	4 (16.7)	0	0	0	2 (66.7)	1 (33.3)	0	1 (33.3)	0
Hypertriglyceridaemia	4 (16.7)	1 (100)	0	1 (33.3)	1 (33.3)	0	0	0	1 (20.0)
Hypoalbuminaemia	4 (16.7)	0	1 (33.3)	0	0	0	1 (33.3)	0	2 (40.0)
N-terminal prohormone brain natriuretic peptide increased	4 (16.7)	1 (100)	1 (33.3)	0	1 (33.3)	0	0	1 (33.3)	0
Bilirubin conjugated increased	3 (12.5)	0	0	1 (33.3)	0	1 (33.3)	0	0	1 (20.0)
Constipation	3 (12.5)	1 (100)	0	0	0	1 (33.3)	0	1 (33.3)	0
Cough	3 (12.5)	0	0	0	0	1 (33.3)	0	2 (66.7)	0
Diarrhoea	3 (12.5)	1 (100)	0	0	0	1 (33.3)	0	0	1 (20.0)
Gastrooesophageal reflux disease	3 (12.5)	0	0	1 (33.3)	1 (33.3)	1 (33.3)	0	0	0
Hyperglycaemia	3 (12.5)	1 (100)	2 (66.7)	0	0	0	0	0	0
Hyperuricaemia	3 (12.5)	0	2 (66.7)	1 (33.3)	0	0	0	0	0
Hyponatraemia	3 (12.5)	0	1 (33.3)	1 (33.3)	0	0	0	0	1 (20.0)
Proteinuria	3 (12.5)	0	1 (33.3)	0	0	1 (33.3)	0	0	1 (20.0)
Pyrexia	3 (12.5)	1 (100)	0	0	0	1 (33.3)	0	1 (33.3)	0
Sinus bradycardia	3 (12.5)	0	1 (33.3)	1 (33.3)	1 (33.3)	0	0	0	0
**Any serious treatment-related adverse event, n (%)**	**10 (41.7)**	**0**	**0**	**1 (33.3)**	**1 (33.3)**	**2 (66.7)**	**1 (33.3)**	**1 (33.3)**	**4 (80.0)**
Anaemia	6 (25.0)	0	0	1 (33.3)	0	1 (33.3)	1 (33.3)	1 (33.3)	2 (40.0)
Neutropenia	2 (8.3)	0	0	0	0	0	0	0	2 (40.0)
Pneumonia	2 (8.3)	0	0	0	0	0	0	1 (33.3)	1 (20.0)
Death	1 (4.2)	0	0	0	0	1 (33.3)	0	0	0
Dizziness	1 (4.2)	0	0	0	1 (33.3)	0	0	0	0
Febrile infection	1 (4.2)	0	0	0	0	0	0	0	1 (20.0)
Febrile neutropenia	1 (4.2)	0	0	0	0	0	0	0	1 (20.0)
Interstitial lung disease	1 (4.2)	0	0	0	0	1 (33.3)	0	0	0
Leukopenia	1 (4.2)	0	0	0	0	0	0	0	1 (20.0)
Myelosuppression	1 (4.2)	0	0	0	0	0	0	0	1 (20.0)
Thrombocytopaenia	1 (4.2)	0	0	0	1 (33.3)	0	0	0	0
**Any treatment-related adverse event leading to death, n (%)**	**2 (8.3)**	**0**	**0**	**0**	**0**	**1 (33.3)**	**0**	**0**	**1 (20.0)**
Death	1 (4.2)	0	0	0	0	1 (33.3)	0	0	0
Pneumonia	1 (4.2)	0	0	0	0	0	0	0	1 (20.0)

**Table 4:** Treatment-related adverse events, serious adverse events, and treatment-related adverse events leading to death for phase 1b (safety population).

	Total (N = 229)		T-Bren D1 Q3W							
			3.8 mg/kg (N = 23)		4.4 mg/kg (N = 151)		5.0 mg/kg (N = 39)		5.6 mg/kg (N = 16)	
	n (%)	95% CI (Upper limit)	n (%)	95% CI (Upper limit)	n (%)	95% CI (Upper limit)	n (%)	95% CI (Upper limit)	n (%)	95% CI (Upper limit)
**Any treatment-related adverse event**	**228 (99.6)**	**100.0**	**23 (100)**	**100.0**	**150 (99.3)**	**100.0**	**39 (100)**	**100.0**	**16 (100)**	**100.0**
AE in ≥10% of total patients										
Anaemia	210 (91.7)	94.5	18 (78.3)	91.0	141 (93.4)	96.4	35 (89.7)	96.4	16 (100)	100.0
Leukopenia	197 (86.0)	89.6	19 (82.6)	93.8	127 (84.1)	88.8	36 (92.3)	97.9	15 (93.8)	99.7
Neutropenia	185 (80.8)	85.0	19 (82.6)	93.8	117 (77.5)	83.0	35 (89.7)	96.4	14 (87.5)	97.7
Thrombocytopaenia	180 (78.6)	83.0	12 (52.2)	70.4	123 (81.5)	86.5	31 (79.5)	89.4	14 (87.5)	97.7
Nausea	142 (62.0)	67.4	18 (78.3)	91.0	91 (60.3)	66.9	24 (61.5)	74.6	9 (56.3)	77.3
Decreased appetite	111 (48.5)	54.1	13 (56.5)	74.2	72 (47.7)	54.7	22 (56.4)	70.0	4 (25.0)	48.4
Aspartate aminotransferase increased	95 (41.5)	47.1	14 (60.9)	77.8	56 (37.1)	44.0	18 (46.2)	60.4	7 (43.8)	66.7
Vomiting	88 (38.4)	44.0	9 (39.1)	58.3	55 (36.4)	43.4	16 (41.0)	55.4	8 (50.0)	72.1
Asthenia	84 (36.7)	42.2	13 (56.5)	74.2	54 (35.8)	42.7	12 (30.8)	45.0	5 (31.3)	54.8
Alopecia	79 (34.5)	40.0	9 (39.1)	58.3	52 (34.4)	41.3	12 (30.8)	45.0	6 (37.5)	60.9
Lymphocyte count decreased	75 (32.8)	38.2	8 (34.8)	54.0	52 (34.4)	41.3	11 (28.2)	42.3	4 (25.0)	48.4
Alanine aminotransferase increased	72 (31.4)	36.9	11 (47.8)	66.5	45 (29.8)	36.5	13 (33.3)	47.7	3 (18.8)	41.7
Stomatitis	66 (28.8)	34.2	7 (30.4)	49.6	43 (28.5)	35.1	12 (30.8)	45.0	4 (25.0)	48.4
Weight decreased	66 (28.8)	34.2	9 (39.1)	58.3	45 (29.8)	36.5	10 (25.6)	39.6	2 (12.5)	34.4
Gamma-glutamyltransferase increased	64 (27.9)	33.2	7 (30.4)	49.6	47 (31.1)	37.9	8 (20.5)	34.0	2 (12.5)	34.4
Hypoalbuminaemia	63 (27.5)	32.8	5 (21.7)	40.4	43 (28.5)	35.1	10 (25.6)	39.6	5 (31.3)	54.8
Blood alkaline phosphatase increased	62 (27.1)	32.3	7 (30.4)	49.6	42 (27.8)	34.4	9 (23.1)	36.8	4 (25.0)	48.4
Constipation	61 (26.6)	31.9	8 (34.8)	54.0	34 (22.5)	28.8	15 (38.5)	52.9	4 (25.0)	48.4
Hypokalaemia	56 (24.5)	29.6	7 (30.4)	49.6	35 (23.2)	29.5	8 (20.5)	34.0	6 (37.5)	60.9
Diarrhoea	55 (24.0)	29.1	5 (21.7)	40.4	37 (24.5)	30.9	7 (17.9)	31.1	6 (37.5)	60.9
Dizziness	29 (12.7)	16.9	3 (13.0)	30.4	20 (13.2)	18.7	4 (10.3)	22.0	2 (12.5)	34.4
Abdominal distension	27 (11.8)	15.9	2 (8.7)	24.9	20 (13.2)	18.7	1 (2.6)	11.6	4 (25.0)	48.4
Occult blood positive	27 (11.8)	15.9	3 (13.0)	30.4	20 (13.2)	18.7	3 (7.7)	18.7	1 (6.3)	26.4
Abdominal pain	25 (10.9)	14.9	4 (17.4)	35.5	17 (11.3)	16.4	3 (7.7)	18.7	1 (6.3)	26.4
Electrocardiogram QT prolonged	25 (10.9)	14.9	4 (17.4)	35.5	14 (9.3)	14.1	7 (17.9)	31.1	0	17.1
Hyponatraemia	25 (10.9)	14.9	3 (13.0)	30.4	18 (11.9)	17.2	2 (5.1)	15.3	2 (12.5)	34.4
**Any serious treatment-related adverse event**	111 (48.5)	54.1	7 (30.4)	49.6	71 (47.0)	54.0	20 (51.3)	65.3	13 (81.3)	94.7
Thrombocytopaenia	55 (24.0)	29.1	1 (4.3)	19.0	35 (23.2)	29.5	11 (28.2)	42.3	8 (50.0)	72.1
Anaemia	46 (20.1)	24.9	1 (4.3)	19.0	31 (20.5)	26.7	8 (20.5)	34.0	6 (37.5)	60.9
Neutropenia	25 (10.9)	14.9	4 (17.4)	35.5	16 (10.6)	15.6	5 (12.8)	25.1	0	17.1
Leukopenia	21 (9.2)	12.9	0	12.2	15 (9.9)	14.9	3 (7.7)	18.7	3 (18.8)	41.7
Febrile neutropenia	12 (5.2)	8.4	0	12.2	8 (5.3)	9.4	1 (2.6)	11.6	3 (18.8)	41.7
Pneumonia	11 (4.8)	7.8	1 (4.3)	19.0	7 (4.6)	8.5	1 (2.6)	11.6	2 (12.5)	34.4
Myelosuppression	5 (2.2)	4.5	0	12.2	4 (2.6)	6.0	1 (2.6)	11.6	0	17.1
Pneumonitis	5 (2.2)	4.5	1 (4.3)	19.0	3 (2.0)	5.1	1 (2.6)	11.6	0	17.1
Vomiting	5 (2.2)	4.5	0	12.2	4 (2.6)	6.0	1 (2.6)	11.6	0	17.1
Gastrointestinal haemorrhage	4 (1.7)	4.0	0	12.2	0	2.0	1 (2.6)	11.6	3 (18.8)	41.7
Interstitial lung disease	4 (1.7)	4.0	2 (8.7)	24.9	1 (0.7)	3.1	1 (2.6)	11.6	0	17.1
Gastrointestinal disorder	3 (1.3)	3.4	0	12.2	2 (1.3)	4.1	1 (2.6)	11.6	0	17.1
Aspartate aminotransferase increased	2 (0.9)	2.7	0	12.2	2 (1.3)	4.1	0	7.4	0	17.1
Asthenia	2 (0.9)	2.7	1 (4.3)	19.0	0	2.0	1 (2.6)	11.6	0	17.1
Chest discomfort	2 (0.9)	2.7	0	12.2	1 (0.7)	3.1	1 (2.6)	11.6	0	17.1
Decreased appetite	2 (0.9)	2.7	0	12.2	2 (1.3)	4.1	0	7.4	0	17.1
Pyrexia	2 (0.9)	2.7	0	12.2	2 (1.3)	4.1	0	7.4	0	17.1
Amylase increased	1 (0.4)	2.1	0	12.2	1 (0.7)	3.1	0	7.4	0	17.1
Bronchopulmonary aspergillosis	1 (0.4)	2.1	0	12.2	0	2.0	0	7.4	1 (6.3)	26.4
Diarrhoea	1 (0.4)	2.1	0	12.2	1 (0.7)	3.1	0	7.4	0	17.1
Dizziness	1 (0.4)	2.1	0	12.2	1 (0.7)	3.1	0	7.4	0	17.1
Gastrointestinal perforation	1 (0.4)	2.1	0	12.2	0	2.0	1 (2.6)	11.6	0	17.1
Haematochezia	1 (0.4)	2.1	0	12.2	0	2.0	0	7.4	1 (6.3)	26.4
Hypokalaemia	1 (0.4)	2.1	0	12.2	0	2.0	1 (2.6)	11.6	0	17.1
Infection	1 (0.4)	2.1	0	12.2	0	2.0	1 (2.6)	11.6	0	17.1
Intestinal obstruction	1 (0.4)	2.1	0	12.2	1 (0.7)	3.1	0	7.4	0	17.1
Intra-abdominal fluid collection	1 (0.4)	2.1	0	12.2	0	2.0	0	7.4	1 (6.3)	26.4
Lymphocyte count decreased	1 (0.4)	2.1	0	12.2	1 (0.7)	3.1	0	7.4	0	17.1
Nausea	1 (0.4)	2.1	0	12.2	1 (0.7)	3.1	0	7.4	0	17.1
Perihepatic abscess	1 (0.4)	2.1	0	12.2	1 (0.7)	3.1	0	7.4	0	17.1
Pulmonary tuberculosis	1 (0.4)	2.1	0	12.2	1 (0.7)	3.1	0	7.4	0	17.1
Transaminases increased	1 (0.4)	2.1	0	12.2	0	2.0	1 (2.6)	11.6	0	17.1
**Any treatment-related adverse event leading to death**	**2 (0.9)**	**2.7**	**1 (4.3)**	**19.0**	**0**	**2.0**	**0**	**7.4**	**1 (6.3)**	**26.4**
Pneumonia	2 (0.9)	2.7	1 (4.3)	19.0	0	2.0	0	7.4	1 (6.3)	26.4

One-sided 95% CI was calculated using Clopper-Pearson method.

Grade 3 or worse TRAEs are summarised in Supplementary Table S6 and sex-disaggregated TRAE data shown in Supplementary Table S8. In dose escalation part, grade ≥3 haematological toxicities occurred in ≥10% of total patients were neutropenia (50%), leukopenia (45.8%), anaemia (41.7%), thrombocytopaenia (16.7%), and lymphocyte count decreased (12.5%). In expansion part, grade ≥3 haematological toxicities occurred in ≥10% of total patients were anaemia (54.6%), neutropenia (48.5%), leukopenia (41.9%), thrombocytopaenia (40.6%), and lymphocyte count decreased (15.3%).

Serious TRAEs occurred in 10 patients (41.7%) in dose-escalation part (Table 3) and 111 patients (48.5%) in expansion part (Table 4). In dose-escalation part, two patients (8.3%) discontinued treatment due to TRAEs: one case of interstitial lung disease (ILD) in 4.4 mg/kg Q3W group and one case of pneumonia in 6.2 mg/kg Q3W group (Supplementary Table S6). In expansion part, 20 patients (8.7%) discontinued treatment due to TRAEs and the most common reason was thrombocytopaenia (n = 9, 3.9%). In dose-escalation part, two patients (8.3%) died due to treatment-related causes: one case of unexplained death in 4.4 mg/kg Q3W group and one case of pneumonia in 6.2 mg/kg Q3W group. In expansion part, two patients (0.9%) died due to pneumonia.

In total, treatment-related pneumonitis of grade 3 or worse was reported in four patients (1.7%) at 3.8 mg/kg (n = 1), 4.4 mg/kg (n = 2), and 5.0 mg/kg (n = 1) dose level in expansion part, including 2 cases of grade 4 and 2 cases of grade 3. Details for management of pneumonitis are presented in Supplementary Material. In dose-escalation part, one patient (4.2%) in 4.4 mg/kg Q3W group experienced grade 3 ILD (Supplementary Table S7). In expansion part, six patients (2.6%) experienced ILD, including two cases of grade 3.

Of the 253 patients enrolled and treated with T-Bren, 22 (8.7%) patients experienced a decrease in LVEF of at least 10% (absolute value) compared with baseline, with a maximum decrease of 18%. Case of cardiac failure was not reported in these patients.

Dose-limiting toxicities were observed in two patients treated at 6.2 mg/kg every 3 weeks, including grade 4 neutropenia with myelosuppression and grade 3 thrombocytopaenia in one patient, and grade 4 febrile neutropenia in another. All DLTs resolved following dose reduction.

### Efficacy

In dose-escalation part, T-Bren achieved an objective response rate (ORR) of 58.3% (95% CI 36.6–77.9) and a confirmed ORR of 54.2% (95% CI 32.8–74.4), with objective responses observed across all dose levels (Table 5). In expansion part, the ORR was 68.0% (95% CI 61.5–74.0) and confirmed ORR was 64.4% (95% CI 57.8–70.7) (Table 6).

**Table 5:** Best overall responses of T-Bren in solid tumours for phase 1a (modified ITT population).

Variables	Total (N = 24)	T-Bren D1D8 Q3W	T-Bren D1 Q3W						
		1.0 mg/kg (N = 1)	2.6 mg/kg (N = 3)	3.2 mg/kg (N = 3)	3.8 mg/kg (N = 3)	4.4 mg/kg (N = 3)	5.0 mg/kg (N = 3)	5.6 mg/kg (N = 3)	6.2 mg/kg (N = 5)
Best overall response, n (%)									
CR	1 (4.2)	0	0	0	0	0	0	1 (33.3)	0
PR	13 (54.2)	1 (100)	2 (66.7)	1 (33.3)	3 (100)	2 (66.7)	1 (33.3)	0	3 (60.0)
cPR	12 (50.0)	1 (100)	2 (66.7)	1 (33.3)	3 (100)	2 (66.7)	1 (33.3)	0	2 (40.0)
SD	7 (29.2)	0	0	0	0	1 (33.3)	2 (66.7)	2 (66.7)	2 (40.0)
PD	3 (12.5)	0	1 (33.3)	2 (66.7)	0	0	0	0	0
ORR, n (% [95% CI])	14 (58.3 [36.6–77.9])	1 (100 [2.5–100])	2 (66.7 [9.4–99.2])	1 (33.3 [0.8–90.6])	3 (100 [29.2–100])	2 (66.7 [9.4–99.2])	1 (33.3 [0.8–90.6])	1 (33.3 [0.8–90.6])	3 (60.0 [14.7–94.7])
ORR confirmed, n (% [95% CI])	13 (54.2 [32.8–74.4])	1 (100 [2.5–100])	2 (66.7 [9.4–99.2])	1 (33.3 [0.8–90.6])	3 (100 [29.2–100])	2 (66.7 [9.4–99.2])	1 (33.3 [0.8–90.6])	1 (33.3 [0.8–90.6])	2 (40.0 [5.3–85.3])
DCR, n (% [95% CI])	21 (87.5 [67.6–97.3])	1 (100 [2.5–100])	2 (66.7 [9.4–99.2])	1 (33.3 [0.8–90.6])	3 (100 [29.2–100])	3 (100 [29.2–100])	3 (100 [29.2–100])	3 (100 [29.2–100])	5 (100 [47.8–100])

**Table 6:** Best overall responses of T-Bren in solid tumours for phase 1b (modified ITT population).

Variables	Total (N = 225)	T-Bren D1 Q3W			
		3.8 mg/kg (N = 23)	4.4 mg/kg (N = 150)	5.0 mg/kg (N = 36)	5.6 mg/kg (N = 16)
Best overall response, n (%)					
CR	8 (3.6)	1 (4.3)	6 (4.0)	1 (2.8)	0
PR	145 (64.4)	14 (60.9)	99 (66.0)	22 (61.1)	10 (62.5)
cPR	137 (60.9)	11 (47.8)	96 (64.0)	21 (58.3)	9 (56.3)
PR ongoing	1 (0.4)	0	1 (0.7)	0	0
SD	56 (24.9)	7 (30.4)	32 (21.3)	12 (33.3)	5 (31.3)
PD	8 (3.6)	0	7 (4.7)	1 (2.8)	0
NE^a^	8 (3.6)	1 (4.3)	6 (4.0)	0	1 (6.3)
ORR, n (% [95% CI]^b^)	153 (68.0 [61.5–74.0])	15 (65.2 [42.7–83.6])	105 (70.0 [62.0–77.2])	23 (63.9 [46.2–79.2])	10 (62.5 [35.4–84.8])
ORR confirmed, n (% [95% CI]^b^)	145 (64.4 [57.8–70.7])	12 (52.2 [30.6–73.2])	102 (68.0 [59.9–75.4])	22 (61.1 [43.5–76.9])	9 (56.3 [29.9–80.2])
DCR, n (% [95% CI]^b^)	209 (92.9 [88.7–95.9])	22 (95.7 [78.1–99.9])	137 (91.3 [85.6–95.3])	35 (97.2 [85.5–99.9])	15 (93.8 [69.8–99.8])

CI, confidence interval; CR, complete response; cPR, confirmed partial response; DCR, disease control rate; NE, non-evaluable; ORR, overall response rate; PD, progressive disease; PR, partial response; SD, stable disease.

a Including patients without post-baseline tumour assessment.

b 95% CI was calculated using Clopper-Pearson method.

**Fig. 2:**
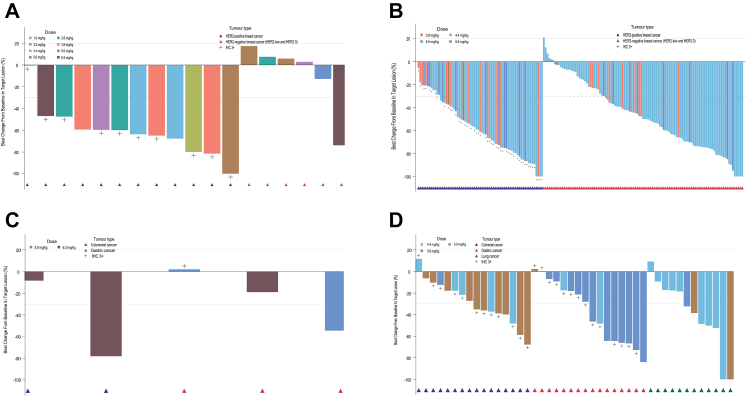
Antitumour activity of T-Bren in solid tumours (modified ITT population). A. Waterfall plot in breast cancer for phase 1a study. B. Waterfall plot in breast cancer for phase 1b study. C. Waterfall plot in gastric cancer and colorectal cancer for phase 1a study. D. Waterfall plot in gastric cancer, lung cancer, and colorectal cancer for phase 1b study. IHC, immunohistochemistry; ITT, intention-to-treat.

Among patients with HER2-positive breast cancer, the ORR was 81.5% (95% CI 71.3–89.2) and the confirmed ORR was 76.5% (95% CI 65.8–85.2), including six patients (7.4%) who achieved a complete response (4.4 mg D1Q3W, ORR, 84.8%; confirmed ORR, 80.4%) (Fig. 2A and B, Table 7). In patients with hormone receptor-positive, HER2-low breast cancer, the ORR was 69.5% (95% CI 58.4–79.2) and the confirmed ORR was 67.1% (95% CI 55.8–77.1) (4.4 mg D1Q3W, both ORR and cORR were 72.9%) (Table 8). In hormone receptor-negative, HER2-low subgroup, both ORR and confirmed ORR were 58.3% (95% CI 36.6–77.9) with three patients (12.5%) achieving a complete response (4.4 mg D1Q3W, both ORR and cORR were 63.6%).

**Table 7:** Best overall response of T-Bren in HER2-positive breast cancer (modified ITT population).

Variables	Total (N = 81)	Phase 1a (N = 12)	Phase 1b (N = 69)	4.4 mg/kg D1Q3W Total (N = 46)	4.4 mg/kg D1Q3W in Phase 1a (N = 3)	4.4 mg/kg D1Q3W in Phase 1b (N = 43)
Best overall response, n (%)						
CR	6 (7.4)	1 (8.3)	5 (7.2)	3 (6.5)	0	3 (7.0)
PR	60 (74.1)	10 (83.3)	50 (72.5)	36 (78.3)	2 (66.7)	34 (79.1)
cPR	56 (69.1)	9 (75.0)	47 (68.1)	34 (73.9)	2 (66.7)	32 (74.4)
SD	13 (16.0)	1 (8.3)	12 (17.4)	5 (10.9)	1 (33.3)	4 (9.3)
NE^a^	2 (2.5)	0	2 (2.9)	2 (4.3)	0	2 (4.7)
ORR, n (% [95% CI]^b^)	66 (81.5 [71.3–89.2])	11 (91.7 [61.5–99.8])	55 (79.7 [68.3–88.4])	39 (84.8 [71.1–93.7])	2 (66.7 [9.4–99.2])	37 (86.0 [72.1–94.7])
ORR confirmed, n (% [95% CI]^b^)	62 (76.5 [65.8–85.2])	10 (83.3 [51.6–97.9])	52 (75.4 [63.5–84.9])	37 (80.4 [66.1–90.6])	2 (66.7 [9.4–99.2])	35 (81.4 [66.6–91.6])
DCR, n (% [95% CI]^b^)	79 (97.5 [91.4–99.7])	12 (100 [73.5–100])	67 (97.1 [89.9–99.6])	44 (95.7 [85.2–99.5])	3 (100 [29.2–100])	41 (95.3 [84.2–99.4])
CBR, n (% [95% CI]^b^)	74 (91.4 [83.0–96.5])	11 (91.7 [61.5–99.8])	63 (91.3 [82.0–96.7])	42 (91.3 [79.2–97.6])	2 (66.7 [9.4–99.2])	40 (93.0 [80.9–98.5])
TTR, months, median (Q1, Q3)	1.4 (1.3, 2.8)	2.2 (1.4, 2.8)	1.4 (1.3, 2.7)	1.4 (1.3, 2.7)	2.0 (1.3, 2.8)	1.4 (1.3, 2.7)

CBR, clinical benefit rate; CI, confidence interval; cPR, confirmed partial response; CR, complete response; cPR, confirmed partial response; D, day; DCR, disease control rate; NE, not evaluable; PD, progressive disease; PR, partial response; Q3W, every 3 weeks; SD, stable disease; TTR, time to response.

a Patients without post-baseline tumour assessment were included.

b 95% CIs of ORR, DCR and CBR were calculated by using the Clopper-Pearson method.

**Table 8:** Best overall response of T-Bren in HER2-low breast cancer (modified ITT population).

Variables	Total	Phase 1a	Phase 1b	4.4 mg/kg D1Q3W Total	4.4 mg/kg D1Q3W in Phase 1a	4.4 mg/kg D1Q3W in Phase 1b
**HR-positive/HER2-low**						
**Number of patients**	**82**	**5**	**77**	**59**	**0**	**59**
Best overall response, n (%)						
PR	57 (69.5)	1 (20.0)	56 (72.7)	43 (72.9)		43 (72.9)
cPR	55 (67.1)	1 (20.0)	54 (70.1)	43 (72.9)		43 (72.9)
SD	19 (23.2)	2 (40.0)	17 (22.1)	13 (22.0)		13 (22.0)
PD	5 (6.1)	2 (40.0)	3 (3.9)	2 (3.4)		2 (3.4)
NE^a^	1 (1.2)	0	1 (1.3)	1 (1.7)		1 (1.7)
ORR, n (% [95% CI]^b^)	57 (69.5 [58.4–79.2])	1 (20.0 [0.5–71.6])	56 (72.7 [61.4–82.3])	43 (72.9 [59.7–83.6])		43 (72.9 [59.7–83.6])
ORR confirmed, n (% [95% CI]^b^)	55 (67.1 [55.8–77.1])	1 (20.0 [0.5–71.6])	54 (70.1 [58.6–80.0])	43 (72.9 [59.7–83.6])		43 (72.9 [59.7–83.6])
DCR, n (% [95% CI]^b^)	76 (92.7 [84.8–97.3])	3 (60.0 [14.7–94.7])	73 (94.8 [87.2–98.6])	56 (94.9 [85.9–98.9])		56 (94.9 [85.9–98.9])
CBR, n (% [95% CI]^b^)	68 (82.9 [73.0–90.3])	2 (40.0 [5.3–85.3])	66 (85.7 [75.9–92.6])	51 (86.4 [75.0–94.0])		51 (86.4 [75.0–94.0])
TTR, months, median (Q1, Q3)	1.6 (1.4, 4.0)	3.0 (3.0, 3.0)	1.6 (1.4, 4.0)	1.6 (1.4, 3.0)		1.6 (1.4, 3.0)
**HR-negative/HER2-low**						
**Number of patients**	**24**	**1**	**23**	**22**	**0**	**22**
Best overall response, n (%)						
CR	3 (12.5)	0	3 (13.0)	3 (13.6)		3 (13.6)
PR	11 (45.8)	0	11 (47.8)	11 (50.0)		11 (50.0)
cPR	11 (45.8)	0	11 (47.8)	11 (50.0)		11 (50.0)
SD	7 (29.2)	1 (100)	6 (26.1)	5 (22.7)		5 (22.7)
PD	1 (4.2)	0	1 (4.3)	1 (4.5)		1 (4.5)
NE	2 (8.3)	0	2 (8.7)	2 (9.1)		2 (9.1)
ORR, n (% [95% CI]^b^)	14 (58.3 [36.6–77.9])	0 (0 [0–97.5])	14 (60.9 [38.5–80.3])	14 (63.6 [40.7–82.8])		14 (63.6 [40.7–82.8])
ORR confirmed, n (% [95% CI]^b^)	14 (58.3 [36.6–77.9])	0 (0 [0–97.5])	14 (60.9 [38.5–80.3])	14 (63.6 [40.7–82.8])		14 (63.6 [40.7–82.8])
DCR, n (% [95% CI]^b^)	21 (87.5 [67.6–97.3])	1 (100 [2.5–100])	20 (87.0 [66.4–97.2])	19 (86.4 [65.1–97.1])		19 (86.4 [65.1–97.1])
CBR, n (% [95% CI]^b^)	19 (79.2 [57.8–92.9])	0 (0 [0–97.5])	19 (82.6 [61.2–95.0])	18 (81.8 [59.7–94.8])		18 (81.8 [59.7–94.8])
TTR, months, median (Q1, Q3)	1.5 (1.4, 2.6)		1.5 (1.4, 2.6)	1.5 (1.4, 2.6)		1.5 (1.4, 2.6)

CBR, clinical benefit rate; CI, confidence interval; CR, complete response; cPR, confirmed partial response; D, day; DCR, disease control rate; HR, hormone receptor; NE, not evaluable; NR, not reached; PD, progressive disease; PR, partial response; Q3W, every 3 weeks; SD, stable disease; TTR, time to response.

Patients with HER2 IHC 0 were not included here.

a Patients without post-baseline tumor assessment were included.

b 95% CIs of ORR, DCR and CBR were calculated by using the Clopper-Pearson method.

Median duration of response was 19.4 months (95% CI 13.7-not reached) in HER2-positive subgroup, 13.7 months (95% CI 7.1–15.5) in hormone receptor-positive, HER2-negative subgroup (HER2-low and HER2 0), and 6.5 months (95% CI 4.2–7.5) in hormone receptor-negative, HER2-negative subgroup (Supplementary Figures S1 and S2).

**Fig. 3:**
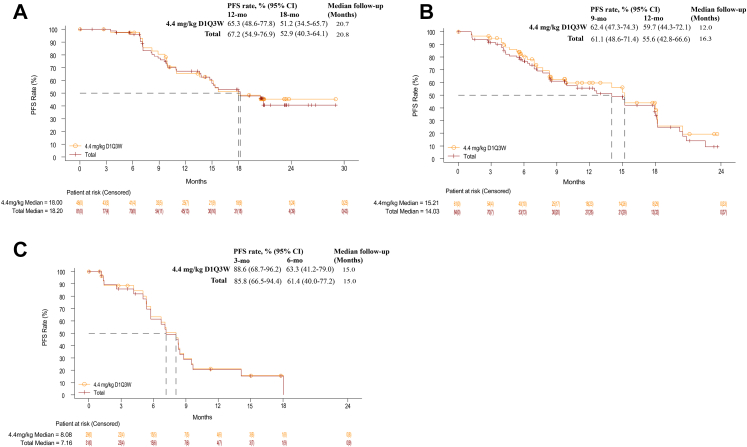
PFS results in breast cancer (modified ITT population). A. Kaplan–Meier plot of PFS in HER2-positive breast cancer (n = 81). B. Kaplan–Meier plot of PFS in HR-positive/HER2-negative (HER2-low and HER2 0) breast cancer (n = 84). C. Kaplan–Meier plot of PFS in HR-negative/HER2-negative (HER2-low and HER2 0) breast cancer (n = 31). HR, hormone receptor; ITT, intention-to-treat; PFS, progression-free survival.

At a median follow-up of 20.8 months, 38 patients (46.9%) with HER2-positive breast cancer had experienced PD or death. Median PFS in this subgroup was 18.2 months (95% CI 13.8-not reached), with 12-month and 18-month PFS rates of 67.2% (95% CI 54.9–76.9) and 52·9% (40.3–64.1), respectively (Fig. 3A). Median PFS was 14.0 months (95% CI 8.5–17.9) in hormone receptor–positive, HER2-negative breast cancer (Fig. 3B) and 7.2 months (5.4–8.8) in hormone receptor–negative, HER2-negative breast cancer (Fig. 3C).

At the time of this analysis, most patients across breast cancer subgroups remained alive. Median OS had not been reached in HER2-positive and hormone receptor-negative, HER2-negative subgroups. In HER2-positive subgroup, 18 patients (22.2%) had died, with an 18-month overall survival rate of 81.4% (95% CI 70.6–88.6). In hormone receptor-positive, HER2-negative subgroup, 23 patients (27.4%) had died, with an 18-month overall survival rate of 74.7% (95% CI 61.5–83.9). Median OS was 27.3 months (95% CI 21.5–NR). In hormone receptor-negative, HER2-negative subgroup, nine patients (29.0%) had died, with an 18-month overall survival rate of 66.1% (95% CI 42.8–81.8).

In patients with other solid tumours, ORR was 50.0% (9/18) in colorectal cancer, 47.4% (9/19) in gastric cancer, and 53.8% (7/13) in lung cancer (Fig. 2C and D, and Supplementary Table S9). Swimmer plot for patients with colorectal cancer, gastric cancer, and lung cancer is presented in Supplementary Figure S3.

Waterfall plots of T-Bren in breast cancer, gastric cancer, and colorectal cancer are presented in Supplementary Figures S4–S6. Swimmer plots of T-Bren in breast cancer, gastric cancer, and colorectal cancer are presented in Supplementary Figures S7–S9. Objective response rates of T-Bren in breast cancer, colorectal cancer, gastric cancer, and lung cancer by HER2 IHC score are presented in Supplementary Figure S10.

### Pharmacokinetics

Pharmacokinetic analyses included 44 patients (cutoff date, 15 July 2025). Plasma exposures of T-Bren and its payload increased with escalating doses, showing approximately linear kinetics across the 2.6–6.2 mg/kg range (Supplementary Table S10, Supplementary Figure S11). The median half-life (t_1_/_2_) of T-Bren ranged from 64 to 85 h, while that of the payload ranged from 107 to 164 h. Across dose groups, higher doses were associated with proportionally higher C_max_ and AUC_0-504_, with no apparent accumulation beyond linear expectations, although limited sample size precludes definitive conclusions.

## Discussion

In this phase 1 study, T-Bren demonstrated a manageable safety profile and encouraging antitumour activity in patients with advanced breast cancer and other solid tumours. In the dose-escalation phase, two patients receiving 6.2 mg/kg Q3W experienced dose-limiting toxicities, establishing the maximum tolerated dose (MTD) at 5.6 mg/kg Q3W. Dose expansion at 3.8, 4.4, 5.0, and 5.6 mg/kg Q3W further evaluated antitumour efficacy, and 4.4 mg/kg Q3W was identified as the recommended phase 2 dose (RP2D) based on an integrated assessment of safety and efficacy.

The safety profile of T-Bren was characterised predominantly by haematologic adverse events, with grade ≥3 anaemia, leukopenia, neutropenia, and thrombocytopaenia observed in up to 54.6% of patients treated at expansion doses (3.8, 4.4, 5.0, and 5.6 mg/kg Q3W). Incidence of treatment-related interstitial lung disease (ILD) was low (2.6% at expansion doses). With appropriate dose modifications or delays, most patients were able to continue treatment, and only 8.7% of patients treated at expansion doses discontinued due to TRAEs.

In this phase 1 study, T-Bren demonstrated encouraging preliminary antitumour activity across all dose levels and a range of tumour types. In the dose-expansion cohort receiving 4.4 mg/kg every three weeks, T-Bren achieved ORR of 84.8%, 72.9%, and 63.6% in patients with HER2-positive, HR-positive/HER2-low, and HR-negative/HER2-low breast cancer, respectively. Corresponding median progression-free survival (PFS) was 18.0 months in HER2-positive, 15.2 months in HR-positive/HER2-negative (including HER2-low and HER2 0), and 8.1 months in HR-negative/HER2-negative breast cancer. Among patients with other solid tumours, include colon, gastric, lung and rectal cancer, 53.7% had HER2 IHC3+ tumours and 44.4% had received prior anti-HER2 therapy. T-Bren demonstrated an ORR of 49% in these patients. These findings collectively support the antitumour activity of T-Bren in HER2-expressing advanced breast cancer and other solid tumours.

Compared with T-Dxd, T-Bren is composed of a topoisomerase I inhibitor Ed-04 with enhanced potency and cleavable peptide linker with improved stability. In cancer cell lines, Ed-04 was more potent than Dxd in inhibiting cancer cell proliferation. In mouse xenograft tumour with heterogeneous HER2 expression levels, T-Bren showed bystander effects comparable to T-Dxd and stronger than T-DM1.^19^

In clinical studies, T-Bren showed higher incidence of grade ≥3 AEs than T-Dxd, which were predominantly haematological toxicities (anaemia, neutropenia, thrombocytopaenia) and could be managed with supportive cares. Rate of treatment discontinuation due to TRAEs was lower with T-Bren, compared with that of T-Dxd in Asian patients in DESTINY-Breast03 (19.7%).^20^ In addition, T-Bren showed lower incidence of ILD compared with results of DESTINY-Breast03 and DESTINY-Breast04 in Asian patients (up to 14.3% with T-Dxd in Asian patients).^20^^,^^21^

In the DESTINY-Breast 01 and 02 studies,^22^^,^^23^ T-Dxd achieved ORRs of 60.9% and 69.7%, with median PFS of 16.4 and 17.8 months in HER2-positive advanced breast cancer.^22^^,^^23^ In our study, heavily pretreated HER2-positive patients (median prior lines of therapy, 3) treated with T-Bren exhibited higher ORR (84.8%) and longer PFS (18.0 months), suggesting a favourable efficacy profile even in later-line settings. The clinical benefit of early-use T-Bren for treating HER2-positive breast cancer is worthwhile to investigate in future clinical studies. For HER2-low breast cancer, T-Bren showed ORRs of 72.9% in HR-positive/HER2-low and 63.6% in HR-negative/HER2-low subgroups, with complete response rates of 13.6% in HR-negative/HER2-low disease. These results compare favourably with DESTINY-Breast 04 study, in which T-Dxd achieved an ORR of 52.3% (52.6% in HR-positive cohort and 50% in HR-negative cohort) and median PFS of 9.9 months (10.1 months in HR-positive cohort and 8.5 months in HR-negative cohort),^24^ highlighting T-Bren's potential to provide meaningful clinical benefit in heavily pretreated HER2-low breast cancer.

Based on these safety and efficacy results, four phase 2 studies (NCT06445400, NCT06031584, NCT06131450, NCT06114511) have been initiated and are ongoing evaluating T-Bren combination therapy or single-agent therapy in HER2-positive breast cancer, HER2-expressing gynaecological malignancies and urinary tumours, and HER2-mutant non-small cell lung cancer (NSCLC). In addition, three phase 3 studies (NCT06316531, NCT06957886, NCT06830889) on T-Bren in advanced HER2-positive and HER2-low breast cancer, and adjuvant treatment of HER2-positive breast cancer, and one phase 2/3 study (NCT06891833) evaluating T-Bren with or without pertuzumab in the neoadjuvant treatment of HER2-positive breast cancer have been initiated and are ongoing.

Limitations of this study include its single-arm, open-label, phase 1 design and restriction to a Chinese patient population, which precludes direct comparison with other therapies. Nevertheless, HER2-stratified analyses provide valuable insights into efficacy across HER2-positive and HER2-low subgroups, supporting the design of subsequent clinical development plans. Secondly, duration of follow-up is short and overall survival data are immature. Thus, the long-term OS benefit of T-Bren in advanced breast cancer and other solid tumours requires further evaluation with extended follow-up. Thirdly, sample size is small, which may limit the assessment of safety.

In conclusion, T-Bren demonstrated a manageable safety profile and encouraging antitumour activity in patients with HER2-positive and HER2-low breast cancer, as well as other solid tumours. The 4.4 mg/kg every three weeks regimen was established as the recommended phase 2 dose (RP2D) in breast cancer, while the optimal RP2D for other solid tumours remains under investigation. These findings support further phase 2 studies of T-Bren combination therapy or single-agent therapy in HER2-positive breast cancer, HER2-expressing gynaecological malignancies and urinary tumours, and HER2-mutant NSCLC, and phase 3 studies in advanced HER2-positive and HER2-low breast cancer, and neoadjuvant and adjuvant treatment of HER2-positive breast cancer.

## Contributors

Conceptualisation: Herui Yao, Hong Zong, Xiuping Lai, Rongbo Lin, Qing Wen, Meili Sun, Erwei Song, Sa Xiao.

Data curation: Herui Yao, Ruihua Zhao, Hong Zong, Xiuping Lai, Rongbo Lin, Qing Wen, Meili Sun, Ying Wang, Suiwen Ye, Jun Jia, Ling Guan, Yongqiang Zhang, Xian Wang, Yahua Zhong, Jianying Huang, Mei Li, Junlan Guo, Fuguo Tian, Yuan Yuan, Erwei Song.

Formal analysis: Herui Yao, Ruihua Zhao, Hong Zong, Xiuping Lai, Rongbo Lin, Qing Wen, Meili Sun, Erwei Song, Sa Xiao, Hai Zhu, Yi Zhu.

Investigation: Herui Yao, Ruihua Zhao, Hong Zong, Xiuping Lai, Rongbo Lin, Qing Wen, Meili Sun, Ying Wang, Suiwen Ye, Jun Jia, Ling Guan, Yongqiang Zhang, Xian Wang, Yahua Zhong, Jianying Huang, Mei Li, Junlan Guo, Fuguo Tian, Yuan Yuan, Erwei Song.

Project administration: Herui Yao, Erwei Song.

Supervision: Herui Yao, Erwei Song.

Validation: All authors.

Writing – original draft: All authors.

Writing – review & editing: All authors.

Herui Yao, Erwei Song, and Xiuping Lai have accessed and verified the underlying data. All authors read and approved the final version of the manuscript.

## Data sharing statement

Study data are available from the authors Herui Yao (yaoherui@mail.sysu.edu.cn) and Erwei Song (songew@mail.sysu.edu.cn) upon reasonable request. A proposal for data sharing needs to be submitted and approved by the study sponsor Baili-Bio (Chengdu) Pharmaceutical Co., Ltd to ensure protection of intellectual property.

## Declaration of interests

Sa Xiao is full-time employee of Baili-Bio (Chengdu) Pharmaceutical Co., Ltd and owns stock of Sichuan Biokin Pharmaceutical Co., Ltd. Hai Zhu is full-time employee of SystImmune Inc and Sichuan Biokin Pharmaceutical Co., Ltd, and owns stock option of SystImmune Inc. Yi Zhu is the president of Sichuan Biokin Pharmaceutical Co., Ltd and Systimmune Inc and owns stock of Sichuan Biokin Pharmaceutical Co., Ltd and stock option of SystImmune Inc. Other authors have no conflicts of interests to disclose.

## Appendix A Supplementary data

Supplementary Material

Clinical Study Protocol
