# Supplementary material for: A multi-centre, phase 1a/1b dose escalation and expansion study of the HER2-directed antibody–drug conjugate T-Bren (BL-M07D1) in advanced breast cancer and other solid tumours
Source: eBioMedicine. 2026 Jun 25;129:106351. doi: 10.1016/j.ebiom.2026.106351 (PMC13320388; doi:10.1016/j.ebiom.2026.106351)

**Supplementary Information**

**Rationale for planned dose levels**

Based on the ICH guideline Nonclinical Evaluation for Anticancer Pharmaceuticals S9, the initial dose was calculated as 1.1 mg/kg QW using safety data from the most relevant or sensitive animal species and 1/6 of highest non-severely toxic dose (HNSTD) in non-rodents. The minimum effective dose was 12 mg/kg QW in mouse subcutaneous xenograft model and the human equivalent dose (HED) was 1.0 mg/kg QW. In addition, the half-life of T-Bren was 37.8 hours at a dose of 3 mg/kg in cynomolgus monkeys (equivalent to 1 mg/kg in humans). Thus, T-Bren administration on days 1 and 8 in a 3-week cycle (D1D8 Q3W) could give patients sufficient recovery from the hematological toxicities induced by the payload. Taken together, the starting dose of 1.0 mg/kg D1D8 Q3W was chosen.

The half-life of T-Bren was 37.8 hours at a dose of 3 mg/kg in cynomolgus monkeys (equivalent to 1 mg/kg in humans), indicating that T-Bren was cleared quickly at a dose of 1.0 mg/kg. Therefore, accelerated titration was applied to the starting dose of 1.0 mg/kg to reduce the number of patients exposed to ineffective or low effect doses.

The initial planned dose levels were 1.0 mg/kg, 3.0, 4.0, 5.0, and 6.0 mg/kg D1D8 Q3W. Starting from 3.0 mg/kg, dose was gradually escalated through three dose increments of 33%, 25%, and 20% up to 6.0 mg/kg for exploring tolerable doses and identifying the optimal therapeutic window. Thereafter, based on the starting dose, the effective dose of the approved HER2-directed ADC T-Dxd, and acquired tolerability data during dose escalation, dose levels of 1.0 mg/kg D1D8 Q3W, 2.6, 3.2, 3.8, 4.4, 5.0, 5.6, 6.2, 6.8, and 7.4 mg/kg D1 Q3W were planned with full consideration of the patient’s benefit-risk ratio. During dose-escalation phase, two out of five patients at 6.2 mg/kg D1 Q3W dose level experienced three DLT events. According to i3+3 rules, no additional patient was enrolled and dose escalation was stopped at 6.2 mg/kg D1 Q3W dose level.

**Missing data handling**

Data used for safety and efficacy analyses were complete. Missing AE terms were analysed as “Uncoded”. Missing causal relationship was analysed as “related”. Missing death/last known alive date was imputed. For date of death, if partially missing, the maximum value among the first day of the year or month, or the last known survival date+1 day was used for imputation. If completely missing, the date was imputed as the last known survival date+1 day. For last known survival date, if both month and day were missing, January 1 of the corresponding year was used for imputation; if only the day was missing, the first day of the corresponding month was used for imputation.

**Database and data handling procedures**

An Electronic Data Capture (EDC) system was used in this study. The study data were entered into electronic case report form (CRF) by the investigator or authorized staff at the study site. The investigator used the electronic signature record to prove that a record had been verified. If the data in eCRF needed to be revised, all changes and reasons for changes were recorded in the audit trail in the EDC system.

**Major protocol amendments**

The original protocol is version 1.0 dated 6 May 2022. In protocol version 1.1 dated 17 August 2022, planned dose levels were changed to 1.0 mg/kg D1D8 Q3W, 2.6, 3.2, 3.8, 4.4, 5.0, 5.6, and 6.2 mg/kg D1 Q3W, aiming to explore the effective doses more precisely. In protocol version 1.2 dated 28 April 2023, dose levels of 6.8 and 7.4 mg/kg D1 Q3W were added based on acquired data during dose escalation. In addition, criteria of dose interruption, dose reduction, and treatment discontinuation for management of neutropenia were elaborated. In protocol version 1.3 dated 1 November 2023, dose reduction strategy was changed from percentage-based adjustment to gradient-based reduction. In protocol version 2.0 dated 7 June 2024, inclusion criteria were revised to include patients with HER2 IHC 0 breast cancer and other solid tumours in the study.

**i3+3 design**

During dose-escalation part, an accelerated titration combined with interval 3+3 (i3+3) design was used. At each dose level, two to four patients (one patient at starting dose level for accelerated titration) were to complete the dose-limiting toxicity (DLT) assessment before moving to the next dose level. If DLT or two episodes of grade≥2 TRAEs (except infusion-related reactions) were observed in the accelerated titration dose group, an additional two to three patients were to be added to the current dose level. If no DLT was observed in the accelerated titration dose group and the episode of grade≥2 TRAEs was less than two, dose escalation was to start from the subsequent dose group. The i3+3 design uses a Bayesian statistical framework and Beta Bernoulli model to pre-calculate dose-increasing decision rules. The parameters of the i3 +3 design in this study will adopt a target DLT rate of 28% and an equivalent interval of 23% ~ 33% (i.e., target DLT rate ±5%) (detailed dose increase/decrease rule is showed below. Dose escalation was to be stopped if maximum sample size was met or a total of ≥six patients were treated at the same dose level and the dose was determined as MTD.

**i3 +3 Detailed Dose Increase/Decrease Rule**


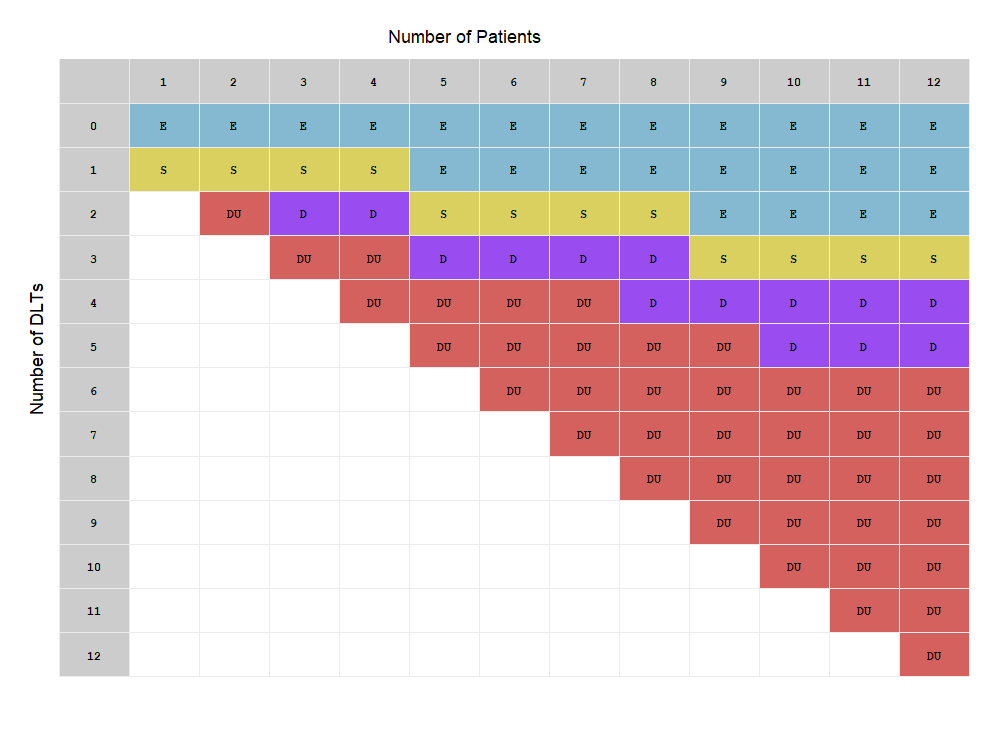


Note 1: Target DLT rates pT=0.28, ε1=0.05, ε2=0.05, equivalent range 0.23~ 0.33.

Note 2: E Dose increase; S Maintain current dose; D Reduce to a previous dose; DU Lower to previous dose and cancel all doses above. Dose increases will only be triggered if the number of subjects in the group is ≥3, otherwise the current dose will be maintained.

**Pneumonia and ILD**

Based on clinical guidelines, the key distinctions between pneumonia and interstitial lung disease (ILD) are as follows:

First, the etiology differs: Pneumonia is primarily infectious (bacterial, viral, fungal, etc.) with identifiable pathogens. In contrast, ILD is often associated with autoimmune disorders, drug toxicity, or environmental exposure, typically showing no evidence of infection.

Second, clinical presentations vary: Pneumonia presents acutely with fever, productive cough, and systemic symptoms (e.g., chills). ILD usually has an insidious onset, characterized by chronic dry cough, exertional dyspnea, and potentially digital clubbing, generally without fever.

Third, radiographic features are distinct: Pneumonia typically shows segmental/lobar infiltrates or consolidation. ILD manifests as diffuse reticular opacities or honeycombing, often accompanied by traction bronchiectasis and reduced lung volume.

Consequently, treatment strategies differ: Pneumonia is treated with anti-infectives (antibiotics/antivirals) and is usually curable. ILD management requires addressing the underlying cause (e.g., drug cessation), followed by corticosteroids, immunosuppressants, or anti-fibrotic agents (Nintedanib/Pirfenidone) to delay progression.

For diagnosis of drug-induced ILD, any other cause of ILD need to be excluded, e.g. infections, cardiopathy, radiotherapy, progression of an underlying ILD or lung condition. Temporal relationship between the onset of symptoms and drug exposure needs to be defined.

**References:**

Conte P, Ascierto P A, Patelli G, et al. Drug-induced interstitial lung disease during cancer therapies: expert opinion on diagnosis and treatment[J]. ESMO open, 2022, 7(2): 100404.

Swain S M, Nishino M, Lancaster L H, et al. Multidisciplinary Clinical Guidance on Trastuzumab Deruxtecan (T-DXd)–Related Interstitial Lung Disease—Focus on Proactive Monitoring, Diagnosis, and Management[J]. Cancer Treatment Reviews, 2022: 102378.

**Guidelines for management of** **pneumonitis/ILD are as follows.**

Grade 1: Close monitoring of symptoms, signs and hematological examinations. If deterioration occurs, refer to management for grade 2–4 events.

Grade 2: Initial prednisolone 0.5 mg/kg/d–1.0 mg/kg/d or equivalent agents for 2–4 weeks. Taper gradually after symptoms and signs resolve; total treatment duration at least 6 weeks.

Grade 3: Initial prednisolone 1.0 mg/kg/d–2.0 mg/kg/d or equivalent agents for 2–4 weeks. Taper gradually after symptoms and signs resolve; total treatment duration at least 8 weeks.

Grade 4: Methylprednisolone 500 mg/d–1000 mg/d as pulse therapy for 3 days, followed by prednisolone 1 mg/kg/d–2 mg/kg/d for 2–4 weeks. Taper gradually after symptoms and signs resolve; total treatment duration at least 8–10 weeks.

Empirical antibiotic therapy (select sensitive antimicrobial agents as needed or based on microbiological results). Oxygen therapy: It is recommended to refer to the oxygen therapy indications for chronic obstructive pulmonary disease. Long-term oxygen therapy for >15 h/day is indicated in ILD patients with resting hypoxemia [arterial partial pressure of oxygen (PaO₂)≤55 mmHg (1 mmHg = 0.133 kPa) or arterial oxygen saturation (SaO₂)≤88%]. Mechanical assisted ventilation is used in case of respiratory failure.

**Reference:**

Chinese Society of Lung Cancer, Chinese Anti-Cancer Association. EGFR-TKI ADR Management Chinese Expert Consensus. Chin J Lung Cancer, February 2019, Vol.22, No.2

**Study Design**


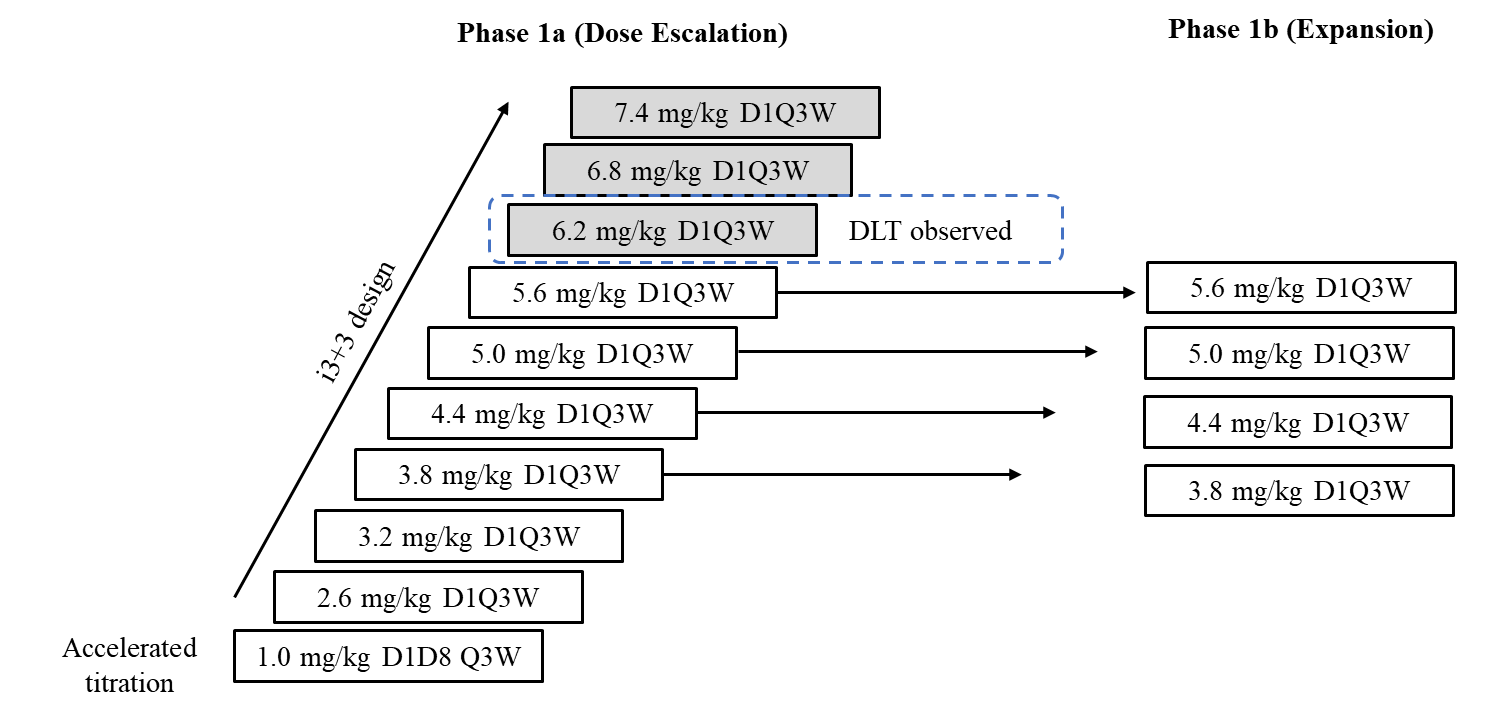


**List of Ethics Committees**

| **Name of Ethics Committee** | **Reference Number** |
| --- | --- |
| Ethics Committee of Sun Yat-sen Memorial Hospital, Sun Yat-sen University | SYSYW-2022-092-01 |
| Ethics Committee of Zhongnan Hospital of Wuhan University | 药伦[2022052] |
| Ethics Committee of Central Hospital Affiliated to Shandong First Medical University | 济中心伦理临审2022-193-01号 |
| Ethics Committee of West China Hospital, Sichuan University | 2022年临床试验(西药)审(301)号 |
| Ethics Committee of Beijing Hospital, National Center of Gerontology, National Health Commission, Institute of Geriatric Medicine, Chinese Academy of Medical Sciences | 2022BJYYEC-331-01 |
| Ethics Committee of Clinical Oncology School of Fujian Medical University, Fujian Cancer Hospital | 2022-145-01 |
| Ethics Committee of Sir Run Run Shaw Hospital, School of Medicine, Zhejiang University | 邵逸夫医院伦审2022药第1062号 |
| Ethics Committee of the First Affiliated Hospital of Zhengzhou University | L2022-Y269-001 |
| Ethics Committee of the Tenth Affiliated Hospital, Southern Medical University (Dongguan People's Hospital) | DRYA2022-049-A1 |
| Ethics Committee of Shanxi Cancer Hospital | YW2022113 |
| Ethics Committee of Xuzhou Central Hospital, Southeast University | XZXY-LY-20220908-082 |
| Ethics Committee of Anyang Tumor Hospital | 2024-01-014-H01 |

Supplementary Table 1 Patient Enrollment by Study Sites

| **Study Site Number** | **Name and Location of Study Site** | **Name of Principal Investigators** | **Number of Patients Enrolled** |
| --- | --- | --- | --- |
| 01 | Sun Yat-sen Memorial Hospital, Sun Yat-sen University, Guangzhou, China | Herui Yao/Erwei Song | 43 |
| 02 | Zhongnan Hospital of Wuhan University, Wuhan, China | Yahua Zhong/Jianying Huang | 8 |
| 03 | Central Hospital Affiliated to Shandong First Medical University, Jinan, China | Qing Wen/Meili Sun | 28 |
| 04 | West China Hospital, Sichuan University, Chengdu, China | Mei Li | 7 |
| 05 | Beijing Hospital, National Center of Gerontology, National Health Commission, Institute of Geriatric Medicine, Chinese Academy of Medical Sciences, Beijing, China | Yongqiang Zhang | 14 |
| 06 | Clinical Oncology School of Fujian Medical University, Fujian Cancer Hospital, Fuzhou, China | Rongbo Lin | 41 |
| 07 | Sir Run Run Shaw Hospital, School of Medicine, Zhejiang University, Hangzhou, China | Xian Wang | 13 |
| 08 | The First Affiliated Hospital of Zhengzhou University, Zhengzhou, China | Hong Zong | 69 |
| 09 | The Tenth Affiliated Hospital, Southern Medical University (Dongguan People's Hospital), Dongguan, China | Jun Jia/Ling Guan | 18 |
| 10 | Shanxi Cancer Hospital, Taiyuan, China | Fuguo Tian | 3 |
| 11 | Xuzhou Central Hospital, Southeast University, Xuzhou, China | Yuan Yuan | 3 |
| 12 | Anyang Tumor Hospital, Anyang, China | Junlan Guo | 6 |

Supplementary Table 2 Patient Demographics and Baseline Characteristics of Patients with HER2-Positive Breast Cancer (Safety Population)

| **Characteristics** | **Total**  **(N=81)** | **Phase 1a**  **(N = 12)** | **Phase 1b**  **(N = 69)** | **4.4 mg/kg D1 Q3W Total**  **(N=46)** | **4.4 mg/kg D1 Q3W Phase 1a**  **(N=3)** | **4.4 mg/kg D1 Q3W Phase 1b**  **(N=43)** |
| --- | --- | --- | --- | --- | --- | --- |
| Age, years, median (Q1, Q3) | 54.0 (45.0, 61.0) | 54.5 (43.5, 56.5) | 54.0 (47.0, 61.0) | 55.0 (45.0, 61.0) | 56.0 (44.0, 67.0) | 55.0 (45.0, 61.0) |
| Sex, n (%) |  |  |  |  |  |  |
| Male | 1 ( 1.2) | 0 | 1 ( 1.4) | 1 ( 2.2) | 0 | 1 ( 2.3) |
| Female | 80 (98.8) | 12 ( 100) | 68 (98.6) | 45 (97.8) | 3 ( 100) | 42 (97.7) |
| Race, n (%) |  |  |  |  |  |  |
| Asian | 81 ( 100) | 12 ( 100) | 69 ( 100) | 46 ( 100) | 3 ( 100) | 43 ( 100) |
| ECOG performance status, n (%) |  |  |  |  |  |  |
| 0 | 16 (19.8) | 3 (25.0) | 13 (18.8) | 8 (17.4) | 1 (33.3) | 7 (16.3) |
| 1 | 65 (80.2) | 9 (75.0) | 56 (81.2) | 38 (82.6) | 2 (66.7) | 36 (83.7) |
| BMI, kg/m^2^, mean (SD) | 24.0 (3.2) | 23.3 (3.1) | 24.1 (3.2) | 23.8 (2.8) | 24.8 (1.6) | 23.7 (2.9) |
| Prior lines of therapy, median (Q1, Q3) | 3.0 (2.0, 5.0) | 3.0 (2.0, 6.0) | 3.0 (1.0, 4.0) | 3.0 (1.0, 4.0) | 3.0 (1.0, 6.0) | 3.0 (1.0, 4.0) |
| Prior lines of therapy, n (%) |  |  |  |  |  |  |
| 1 | 19 (23.5) | 1 ( 8.3) | 18 (26.1) | 12 (26.1) | 1 (33.3) | 11 (25.6) |
| 2 | 15 (18.5) | 4 (33.3) | 11 (15.9) | 8 (17.4) | 0 | 8 (18.6) |
| ≥3 | 47 (58.0) | 7 (58.3) | 40 (58.0) | 26 (56.5) | 2 (66.7) | 24 (55.8) |
| Previous chemotherapy, n (%) | 81 ( 100) | 12 ( 100) | 69 ( 100) | 46 ( 100) | 3 ( 100) | 43 ( 100) |
| Previous anti-HER2 therapy, n (%) | 79 (97.5) | 12 ( 100) | 67 (97.1) | 44 (95.7) | 3 ( 100) | 41 (95.3) |
| Previous CDK4/6 inhibitors, n (%) | 7 ( 8.6) | 0 | 7 (10.1) | 4 ( 8.7) | 0 | 4 ( 9.3) |
| Previous endocrine therapy, n (%) | 35 (43.2) | 5 (41.7) | 30 (43.5) | 19 (41.3) | 1 (33.3) | 18 (41.9) |
| HER2 status, n (%) |  |  |  |  |  |  |
| IHC 2+ and ISH+ | 19 (23.5) | 2 (16.7) | 17 (24.6) | 10 (21.7) | 1 (33.3) | 9 (20.9) |
| IHC 3+ | 62 (76.5) | 10 (83.3) | 52 (75.4) | 36 (78.3) | 2 (66.7) | 34 (79.1) |
| Hormone receptor status, n (%) |  |  |  |  |  |  |
| Positive | 51 (63.0) | 9 (75.0) | 42 (60.9) | 27 (58.7) | 1 (33.3) | 26 (60.5) |
| Negative | 29 (35.8) | 3 (25.0) | 26 (37.7) | 18 (39.1) | 2 (66.7) | 16 (37.2) |
| ER-negative/PR missing | 1 ( 1.2) | 0 | 1 ( 1.4) | 1 ( 2.2) | 0 | 1 ( 2.3) |
| Number of metastatic sites, n (%) |  |  |  |  |  |  |
| 1-2 | 38 (46.9) | 5 (41.7) | 33 (47.8) | 21 (45.7) | 2 (66.7) | 19 (44.2) |
| ≥3 | 43 (53.1) | 7 (58.3) | 36 (52.2) | 25 (54.3) | 1 (33.3) | 24 (55.8) |
| Baseline lung metastases, n (%) | 47 (58.0) | 6 (50.0) | 41 (59.4) | 28 (60.9) | 2 (66.7) | 26 (60.5) |
| Baseline liver metastases, n (%) | 27 (33.3) | 4 (33.3) | 23 (33.3) | 16 (34.8) | 0 | 16 (37.2) |
| Baseline bone metastases, n (%) | 36 (44.4) | 6 (50.0) | 30 (43.5) | 24 (52.2) | 2 (66.7) | 22 (51.2) |
| Baseline brain metastases, n (%) | 9 (11.1) | 1 ( 8.3) | 8 (11.6) | 8 (17.4) | 1 (33.3) | 7 (16.3) |
| Baseline SOD, mm, median (Q1, Q3) | 46.8 (27.5, 88.8) | 34.5 (29.5, 68.4) | 49.7 (26.3, 88.8) | 57.9 (31.0, 98.7) | 96.7 (31.0, 223.6) | 56.7 (28.3, 98.7) |

BMI, body mass index; CDK, cyclin-dependent kinases; ECOG, Eastern Cooperative Oncology Group; ER, estrogen receptor; PR, progesterone receptor; SOD, sum of diameter

Note: HER2-positive: HER2 IHC 3+ or IHC 2+/ISH+.

Supplementary Table 3 Patient Demographics and Baseline Characteristics of Patients with HER2-Negative Breast Cancer (Safety Population)

| **Characteristics** | **Total**  **(N=116)** | **Phase 1a**  **(N = 6)** | **Phase 1b**  **(N = 110)** | **4.4 mg/kg D1 Q3W Total**  **(N=91)** | **4.4 mg/kg D1 Q3W Phase 1a**  **(N=0)** | **4.4 mg/kg D1 Q3W Phase 1b**  **(N=91)** |
| --- | --- | --- | --- | --- | --- | --- |
| Age, years, median (Q1, Q3) | 54.0 (47.0, 60.0) | 51.0 (42.0, 55.0) | 54.0 (47.0, 60.0) | 55.0 (48.0, 61.0) |  | 55.0 (48.0, 61.0) |
| Sex, n (%) |  |  |  |  |  |  |
| Male | 2 ( 1.7) | 0 | 2 ( 1.8) | 1 ( 1.1) |  | 1 ( 1.1) |
| Female | 114 (98.3) | 6 ( 100) | 108 (98.2) | 90 (98.9) |  | 90 (98.9) |
| Race, n (%) |  |  |  |  |  |  |
| Asian | 116 ( 100) | 6 ( 100) | 110 ( 100) | 91 ( 100) |  | 91 ( 100) |
| ECOG performance status, n (%) |  |  |  |  |  |  |
| 0 | 21 (18.1) | 2 (33.3) | 19 (17.3) | 17 (18.7) |  | 17 (18.7) |
| 1 | 95 (81.9) | 4 (66.7) | 91 (82.7) | 74 (81.3) |  | 74 (81.3) |
| BMI, kg/m^2^, mean (SD) | 23.7 (3.8) | 23.7 (4.1) | 23.7 (3.8) | 23.7 (3.6) |  | 23.7 (3.6) |
| Prior lines of therapy, median (Q1, Q3) | 2.0 (2.0, 4.0) | 2.5 (2.0, 5.0) | 2.0 (2.0, 4.0) | 2.0 (2.0, 3.0) |  | 2.0 (2.0, 3.0) |
| Prior lines of therapy, n (%) |  |  |  |  |  |  |
| 1 | 19 (16.4) | 0 | 19 (17.3) | 16 (17.6) |  | 16 (17.6) |
| 2 | 42 (36.2) | 3 (50.0) | 39 (35.5) | 35 (38.5) |  | 35 (38.5) |
| ≥3 | 55 (47.4) | 3 (50.0) | 52 (47.3) | 40 (44.0) |  | 40 (44.0) |
| Previous chemotherapy, n (%) | 109 (94.0) | 6 ( 100) | 103 (93.6) | 84 (92.3) |  | 84 (92.3) |
| Previous anti-HER2 therapy, n (%) | 15 (12.9) | 2 (33.3) | 13 (11.8) | 10 (11.0) |  | 10 (11.0) |
| Previous CDK4/6 inhibitors, n (%) | 69 (59.5) | 6 ( 100) | 63 (57.3) | 53 (58.2) |  | 53 (58.2) |
| Previous endocrine therapy, n (%) | 87 (75.0) | 6 ( 100) | 81 (73.6) | 65 (71.4) |  | 65 (71.4) |
| HER2 status, n (%) |  |  |  |  |  |  |
| IHC 0 | 10 ( 8.6) | 0 | 10 ( 9.1) | 10 (11.0) |  | 10 (11.0) |
| IHC 1+ | 47 (40.5) | 3 (50.0) | 44 (40.0) | 35 (38.5) |  | 35 (38.5) |
| IHC 2+ and ISH- | 59 (50.9) | 3 (50.0) | 56 (50.9) | 46 (50.5) |  | 46 (50.5) |
| Hormone receptor status, n (%) |  |  |  |  |  |  |
| Positive | 85 (73.3) | 5 (83.3) | 80 (72.7) | 62 (68.1) |  | 62 (68.1) |
| Negative | 31 (26.7) | 1 (16.7) | 30 (27.3) | 29 (31.9) |  | 29 (31.9) |
| Number of metastatic sites, n (%) |  |  |  |  |  |  |
| 1-2 | 34 (29.3) | 2 (33.3) | 32 (29.1) | 30 (33.0) |  | 30 (33.0) |
| ≥3 | 82 (70.7) | 4 (66.7) | 78 (70.9) | 61 (67.0) |  | 61 (67.0) |
| Baseline lung metastases, n (%) | 58 (50.0) | 1 (16.7) | 57 (51.8) | 44 (48.4) |  | 44 (48.4) |
| Baseline liver metastases, n (%) | 69 (59.5) | 5 (83.3) | 64 (58.2) | 53 (58.2) |  | 53 (58.2) |
| Baseline bone metastases, n (%) | 74 (63.8) | 4 (66.7) | 70 (63.6) | 53 (58.2) |  | 53 (58.2) |
| Baseline brain metastases, n (%) | 10 ( 8.6) | 0 | 10 ( 9.1) | 8 ( 8.8) |  | 8 ( 8.8) |
| Baseline SOD, mm, median (Q1, Q3) | 50.0 (31.9, 82.1) | 46.0 (38.3, 97.2) | 50.0 (31.8, 79.9) | 48.9 (28.2, 77.2) |  | 48.9 (28.2, 77.2) |

BMI, body mass index; CDK, cyclin-dependent kinases; ECOG, Eastern Cooperative Oncology Group; HR, hormone receptor; SOD, sum of diameter

Note: HER2-negative: HER2 IHC2+/ISH-, IHC1+, or IHC0.

Supplementary Table 4 Patient Demographics and Baseline Characteristics of Patients with Solid Tumours other than Breast Cancer (Safety Population)

| **Characteristics** | **Total^[1]^** | | | **Colorectal Cancer** | | | | **Gastric Cancer** | | | | **Lung Cancer^[2]^** |  |
| --- | --- | --- | --- | --- | --- | --- | --- | --- | --- | --- | --- | --- | --- |
|  | **Total**  **(N=54)** | **Phase 1a**  **(N=6)** | **Phase 1b**  **(N=48)** | **Total**  **(N=19)** | **Phase 1a**  **(N=2)** | **Phase 1b**  **(N=17)** | **Total**  **(N=21)** | | **Phase 1a**  **(N=3)** | **Phase 1b**  **(N=18)** | **Total**  **(N=13)** | | |
| Age, years, median (Q1, Q3) | 55.5 (51.0, 64.0) | 56.5 (47.0, 62.0) | 55.5 (52.0, 64.0) | 54.0 (49.0, 63.0) | 56.5 (53.0, 60.0) | 54.0 (49.0, 63.0) | 56.0 (51.0, 64.0) | | 47.0 (33.0, 67.0) | 57.0 (53.0, 64.0) | 60.0 (53.0, 64.0) | | |
| Sex, n (%) |  |  |  |  |  |  |  | |  |  |  | | |
| Male | 30 (55.6) | 2 (33.3) | 28 (58.3) | 11 (57.9) | 0 | 11 (64.7) | 14 (66.7) | | 2 (66.7) | 12 (66.7) | 5 (38.5) | | |
| Female | 24 (44.4) | 4 (66.7) | 20 (41.7) | 8 (42.1) | 2 ( 100) | 6 (35.3) | 7 (33.3) | | 1 (33.3) | 6 (33.3) | 8 (61.5) | | |
| Race, n (%) |  |  |  |  |  |  |  | |  |  |  | | |
| Asian | 54 ( 100) | 6 ( 100) | 48 (100) | 19 (100) | 2 ( 100) | 17 (100) | 21 (100) | | 3 ( 100) | 18 (100) | 13 (100) | | |
| ECOG performance status, n (%) |  |  |  |  |  |  |  | |  |  |  | | |
| 0 | 4 ( 7.4) | 2 (33.3) | 2 ( 4.2) | 1 (5.3) | 1 (50.0) | 0 | 3 (14.3) | | 1 (33.3) | 2 (11.1) | 0 | | |
| 1 | 50 (92.6) | 4 (66.7) | 46 (95.8) | 18 (94.7) | 1 (50.0) | 17 ( 100) | 18 (85.7) | | 2 (66.7) | 16 (88.9) | 13 ( 100) | | |
| BMI, kg/m^2^, mean (SD) | 22.4 (3.9) | 21.2 (2.8) | 22.6 (4.0) | 23.9 (3.5) | 24.0 (1.8) | 23.9 (3.7) | 21.2 (4.5) | | 20.0 (2.3) | 21.4 (4.8) | 22.4 (2.8) | | |
| Prior lines of therapy, median (Q1, Q3) | 2.0 (1.0, 3.0) | 2.0 (2.0, 3.0) | 2.0 (1.0, 3.0) | 3.0 (2.0, 3.0) | 2.5 (2.0, 3.0) | 3.0 (2.0, 3.0) | 2.0 (1.0, 2.0) | | 2.0 (2.0, 4.0) | 1.0 (1.0, 2.0) | 2.0 (1.0, 2.0) | | |
| Prior lines of therapy, n (%) |  |  |  |  |  |  |  | |  |  |  | | |
| 1 | 17 (31.5) | 0 | 17 (35.4) | 3 (15.8) | 0 | 3 (17.6) | 10 (47.6) | | 0 | 10 (55.6) | 4 (30.8) | | |
| 2 | 19 (35.2) | 4 (66.7) | 15 (31.3) | 5 (26.3) | 1 (50.0) | 4 (23.5) | 6 (28.6) | | 2 (66.7) | 4 (22.2) | 7 (53.8) | | |
| ≥3 | 18 (33.3) | 2 (33.3) | 16 (33.3) | 11 (57.9) | 1 (50.0) | 10 (58.8) | 5 (23.8) | | 1 (33.3) | 4 (22.2) | 2 (15.4) | | |
| Previous chemotherapy, n (%) | 52 (96.3) | 6 ( 100) | 46 (95.8) | 18 (94.7) | 2 ( 100) | 16 (94.1) | 21 (100) | | 3 ( 100) | 18 ( 100) | 12 (92.3) | | |
| Previous anti-HER2 therapy, n (%) | 24 (44.4) | 3 (50.0) | 21 (43.8) | 7 (36.8) | 1 (50.0) | 6 (35.3) | 13 (61.9) | | 1 (33.3) | 12 (66.7) | 3 (23.1) | | |
| HER2 status, n (%) |  |  |  |  |  |  |  | |  |  |  | | |
| IHC 0 | 2 ( 3.7) | 0 | 2 ( 4.2) | 0 | 0 | 0 | 2 (9.5) | | 0 | 2 (11.1) | 0 | | |
| IHC 1+ | 2 ( 3.7) | 1 (16.7) | 1 ( 2.1) | 0 | 0 | 0 | 1 (4.8) | | 1 (33.3) | 0 | 1 (7.7) | | |
| IHC 2+ and ISH+ | 7 (13.0) | 0 | 7 (14.6) | 4 (21.1) | 0 | 4 (23.5) | 2 (9.5) | | 0 | 2 (11.1) | 1 (7.7) | | |
| IHC 2+ and ISH missing | 4 (7.4) | 3 (50.0) | 1 ( 2.1) | 2 (10.5) | 2 ( 100) | 0 | 1 (4.8) | | 1 (33.3) | 0 | 1 (7.7) | | |
| IHC 3+ | 29 (53.7) | 2 (33.3) | 27 (56.3) | 13 (68.4) | 0 | 13 (76.5) | 15 (71.4) | | 1 (33.3) | 14 (77.8) | 0 | | |
| HER2 mutation | 10 (18.5) | 0 | 10 (20.8) | 0 | 0 | 0 | 0 | | 0 | 0 | 10 (76.9) | | |
| Number of metastatic sites, n (%) |  |  |  |  |  |  |  | |  |  |  | | |
| 0 | 1 ( 1.9) | 0 | 1 ( 2.1) | 1 (5.3) | 0 | 1 ( 5.9) | 0 | | 0 | 0 | 0 | | |
| 1-2 | 22 (40.7) | 2 (33.3) | 20 (41.7) | 7 (36.8) | 0 | 7 (41.2) | 12 (57.1) | | 2 (66.7) | 10 (55.6) | 3 (23.1) | | |
| ≥3 | 31 (57.4) | 4 (66.7) | 27 (56.3) | 11 (57.9) | 2 ( 100) | 9 (52.9) | 9 (42.9) | | 1 (33.3) | 8 (44.4) | 10 (76.9) | | |
| Baseline lung metastases, n (%) | 23 (42.6) | 3 (50.0) | 20 (41.7) | 10 (52.6) | 1 (50.0) | 9 (52.9) | 3 (14.3) | | 2 (66.7) | 1 ( 5.6) | 10 (76.9) | | |
| Baseline liver metastases, n (%) | 28 (51.9) | 1 (16.7) | 27 (56.3) | 12 (63.2) | 0 | 12 (70.6) | 11 (52.4) | | 0 | 11 (61.1) | 4 (30.8) | | |
| Baseline bone metastases, n (%) | 13 (24.1) | 1 (16.7) | 12 (25.0) | 4 (21.1) | 0 | 4 (23.5) | 2 (9.5) | | 1 (33.3) | 1 ( 5.6) | 7 (53.8) | | |
| Baseline brain metastases, n (%) | 2 ( 3.7) | 0 | 2 ( 4.2) | 0 | 0 | 0 | 0 | | 0 | 0 | 2 (15.4) | | |
| Baseline SOD, mm, median (Q1, Q3) | 50.2 (34.8, 86.4) | 63.5 (49.2, 144.0) | 46.3 (33.4, 86.1) | 58.4 (41.0, 163.2) | 109.0 (54.7, 163.2) | 58.4 (41.0, 140.7) | 46.6 (34.8, 65.0) | | 49.2 (33.2, 144.0) | 46.3 (34.8, 65.0) | 37.0 (27.0, 82.0) | | |

Note：

1. One patient had the diagnosis of adenocarcinoma and primary tumor site was not recorded.
2. All patients in the lung cancer group were from phase 1b.

**Supplementary Table 5 Safety Summary (Safety Population)**

|  | | **T-Bren D1D8 Q3W** | **T-Bren D1 Q3W** | | | | | | |
| --- | --- | --- | --- | --- | --- | --- | --- | --- | --- |
|  | **Total** | **1.0 mg/kg** | **2.6 mg/kg** | **3.2 mg/kg** | **3.8 mg/kg** | **4.4 mg/kg** | **5.0 mg/kg** | **5.6 mg/kg** | **6.2 mg/kg** |
| **Phase 1a** |  |  |  |  |  |  |  |  |  |
| **Number of Patients** | **24** | **1** | **3** | **3** | **3** | **3** | **3** | **3** | **5** |
| TEAEs | 24 ( 100) | 1 ( 100) | 3 ( 100) | 3 ( 100) | 3 ( 100) | 3 ( 100) | 3 ( 100) | 3 ( 100) | 5 ( 100) |
| Treatment-related | 24 ( 100) | 1 ( 100) | 3 ( 100) | 3 ( 100) | 3 ( 100) | 3 ( 100) | 3 ( 100) | 3 ( 100) | 5 ( 100) |
| Grade≥3 TEAEs | 21 (87.5) | 1 ( 100) | 3 ( 100) | 2 (66.7) | 3 ( 100) | 3 ( 100) | 2 (66.7) | 3 ( 100) | 4 (80.0) |
| Treatment-related | 18 (75.0) | 1 ( 100) | 2 (66.7) | 1 (33.3) | 2 (66.7) | 3 ( 100) | 2 (66.7) | 3 ( 100) | 4 (80.0) |
| Serious TEAEs | 14 (58.3) | 0 | 0 | 2 (66.7) | 3 ( 100) | 3 ( 100) | 1 (33.3) | 1 (33.3) | 4 (80.0) |
| Treatment-related | 10 (41.7) | 0 | 0 | 1 (33.3) | 1 (33.3) | 2 (66.7) | 1 (33.3) | 1 (33.3) | 4 (80.0) |
| TEAEs leading to dose reductions | 12 (50.0) | 0 | 1 (33.3) | 1 (33.3) | 2 (66.7) | 2 (66.7) | 1 (33.3) | 2 (66.7) | 3 (60.0) |
| Treatment-related | 12 (50.0) | 0 | 1 (33.3) | 1 (33.3) | 2 (66.7) | 2 (66.7) | 1 (33.3) | 2 (66.7) | 3 (60.0) |
| TEAEs leading to dose delay | 18 (75.0) | 0 | 2 (66.7) | 2 (66.7) | 3 ( 100) | 3 ( 100) | 1 (33.3) | 3 ( 100) | 4 (80.0) |
| Treatment-related | 11 (45.8) | 0 | 1 (33.3) | 0 | 0 | 2 (66.7) | 1 (33.3) | 3 ( 100) | 4 (80.0) |
| TEAEs leading to treatment discontinuation | 2 ( 8.3) | 0 | 0 | 0 | 0 | 1 (33.3) | 0 | 0 | 1 (20.0) |
| Treatment-related | 2 ( 8.3) | 0 | 0 | 0 | 0 | 1 (33.3) | 0 | 0 | 1 (20.0) |
| TEAEs leading to death | 2 ( 8.3) | 0 | 0 | 0 | 0 | 1 (33.3) | 0 | 0 | 1 (20.0) |
| Treatment-related | 2 ( 8.3) | 0 | 0 | 0 | 0 | 1 (33.3) | 0 | 0 | 1 (20.0) |
| **Phase 1b** |  |  |  |  |  |  |  |  |  |
| **Number of Patients** | **229** | **/** | **/** | **/** | **23** | **151** | **39** | **16** | **/** |
| TEAEs | 228 (99.6) |  |  |  | 23 ( 100) | 150 (99.3) | 39 ( 100) | 16 ( 100) |  |
| Treatment-related | 228 (99.6) |  |  |  | 23 ( 100) | 150 (99.3) | 39 ( 100) | 16 ( 100) |  |
| Grade≥3 TEAEs | 187 (81.7) |  |  |  | 17 (73.9) | 123 (81.5) | 32 (82.1) | 15 (93.8) |  |
| Treatment-related | 184 (80.3) |  |  |  | 16 (69.6) | 121 (80.1) | 32 (82.1) | 15 (93.8) |  |
| Serious TEAEs | 134 (58.5) |  |  |  | 11 (47.8) | 87 (57.6) | 22 (56.4) | 14 (87.5) |  |
| Treatment-related | 111 (48.5) |  |  |  | 7 (30.4) | 71 (47.0) | 20 (51.3) | 13 (81.3) |  |
| TEAEs leading to dose reductions | 116 (50.7) |  |  |  | 6 (26.1) | 78 (51.7) | 22 (56.4) | 10 (62.5) |  |
| Treatment-related | 116 (50.7) |  |  |  | 6 (26.1) | 78 (51.7) | 22 (56.4) | 10 (62.5) |  |
| TEAEs leading to dose delay | 134 (58.5) |  |  |  | 12 (52.2) | 90 (59.6) | 22 (56.4) | 10 (62.5) |  |
| Treatment-related | 122 (53.3) |  |  |  | 10 (43.5) | 83 (55.0) | 20 (51.3) | 9 (56.3) |  |
| TEAEs leading to treatment discontinuation | 24 (10.5) |  |  |  | 3 (13.0) | 13 ( 8.6) | 2 ( 5.1) | 6 (37.5) |  |
| Treatment-related | 20 ( 8.7) |  |  |  | 3 (13.0) | 10 ( 6.6) | 2 ( 5.1) | 5 (31.3) |  |
| TEAEs leading to death | 2 ( 0.9) |  |  |  | 1 ( 4.3) | 0 | 0 | 1 ( 6.3) |  |
| Treatment-related | 2 ( 0.9) |  |  |  | 1 ( 4.3) | 0 | 0 | 1 ( 6.3) |  |

TEAE, treatment-emergent adverse event
Note: Data presented as n (%).

**Supplementary Table 6 Grade≥3 Treatment-Related Adverse Events and Treatment-Related Adverse Events Leading to Treatment Discontinuations (Safety Population)**

|  | | **T-Bren D1D8 Q3W** | **T-Bren D1 Q3W** | | | | | | |
| --- | --- | --- | --- | --- | --- | --- | --- | --- | --- |
|  | **Total** | **1.0 mg/kg** | **2.6 mg/kg** | **3.2 mg/kg** | **3.8 mg/kg** | **4.4 mg/kg** | **5.0 mg/kg** | **5.6 mg/kg** | **6.2 mg/kg** |
| **Phase 1a** |  |  |  |  |  |  |  |  |  |
| **Number of Patients** | **24** | **1** | **3** | **3** | **3** | **3** | **3** | **3** | **5** |
| Any grade≥3 treatment-related adverse event, n (%) | 18 (75.0) | 1 ( 100) | 2 (66.7) | 1 (33.3) | 2 (66.7) | 3 ( 100) | 2 (66.7) | 3 ( 100) | 4 (80.0) |
| Neutropenia | 12 (50.0) | 1 ( 100) | 1 (33.3) | 1 (33.3) | 2 (66.7) | 1 (33.3) | 2 (66.7) | 1 (33.3) | 3 (60.0) |
| Leukopenia | 11 (45.8) | 0 | 1 (33.3) | 1 (33.3) | 1 (33.3) | 1 (33.3) | 2 (66.7) | 2 (66.7) | 3 (60.0) |
| Anaemia | 10 (41.7) | 0 | 0 | 1 (33.3) | 0 | 3 ( 100) | 1 (33.3) | 1 (33.3) | 4 (80.0) |
| Thrombocytopenia | 4 (16.7) | 0 | 0 | 0 | 0 | 0 | 0 | 2 (66.7) | 2 (40.0) |
| Lymphocyte count decreased | 3 (12.5) | 0 | 1 (33.3) | 1 (33.3) | 0 | 0 | 0 | 0 | 1 (20.0) |
| Pneumonia | 2 (8.3) | 0 | 0 | 0 | 0 | 0 | 0 | 1 (33.3) | 1 (20.0) |
| Death | 1 (4.2) | 0 | 0 | 0 | 0 | 1 (33.3) | 0 | 0 | 0 |
| Febrile infection | 1 (4.2) | 0 | 0 | 0 | 0 | 0 | 0 | 0 | 1 (20.0) |
| Febrile neutropenia | 1 (4.2) | 0 | 0 | 0 | 0 | 0 | 0 | 0 | 1 (20.0) |
| Gamma-glutamyltransferase increased | 1 (4.2) | 0 | 0 | 0 | 0 | 1 (33.3) | 0 | 0 | 0 |
| Hyponatraemia | 1 (4.2) | 0 | 1 (33.3) | 0 | 0 | 0 | 0 | 0 | 0 |
| Interstitial lung disease | 1 (4.2) | 0 | 0 | 0 | 0 | 1 (33.3) | 0 | 0 | 0 |
| Myelosuppression | 1 (4.2) | 0 | 0 | 0 | 0 | 0 | 0 | 0 | 1 (20.0) |
| Stomatitis | 1 (4.2) | 0 | 0 | 0 | 0 | 1 (33.3) | 0 | 0 | 0 |
| Vomiting | 1 (4.2) | 0 | 0 | 0 | 1 (33.3) | 0 | 0 | 0 | 0 |
| Any treatment-related adverse event leading to treatment discontinuation, n (%) | 2 (8.3) | 0 | 0 | 0 | 0 | 1 (33.3) | 0 | 0 | 1 (20.0) |
| Interstitial lung disease | 1 (4.2) | 0 | 0 | 0 | 0 | 1 (33.3) | 0 | 0 | 0 |
| Pneumonia | 1 (4.2) | 0 | 0 | 0 | 0 | 0 | 0 | 0 | 1 (20.0) |
| **Phase 1b** |  |  |  |  |  |  |  |  |  |
| **Number of Patients** | **229** | **/** | **/** | **/** | **23** | **151** | **39** | **16** | **/** |
| Any grade≥3 treatment-related adverse event, n (%) | 184 (80.3) |  |  |  | 16 (69.6) | 121 (80.1) | 32 (82.1) | 15 (93.8) |  |
| Anaemia | 125 (54.6) |  |  |  | 8 (34.8) | 82 (54.3) | 22 (56.4) | 13 (81.3) |  |
| Neutropenia | 111 (48.5) |  |  |  | 8 (34.8) | 70 (46.4) | 25 (64.1) | 8 (50.0) |  |
| Leukopenia | 96 (41.9) |  |  |  | 6 (26.1) | 63 (41.7) | 16 (41.0) | 11 (68.8) |  |
| Thrombocytopenia | 93 (40.6) |  |  |  | 4 (17.4) | 61 (40.4) | 17 (43.6) | 11 (68.8) |  |
| Lymphocyte count decreased | 35 (15.3) |  |  |  | 5 (21.7) | 25 (16.6) | 2 (5.1) | 3 (18.8) |  |
| Febrile neutropenia | 13 (5.7) |  |  |  | 0 | 8 (5.3) | 1 (2.6) | 4 (25.0) |  |
| Hypokalaemia | 12 (5.2) |  |  |  | 1 (4.3) | 6 (4.0) | 2 (5.1) | 3 (18.8) |  |
| Pneumonia | 8 (3.5) |  |  |  | 1 (4.3) | 4 (2.6) | 1 (2.6) | 2 (12.5) |  |
| Asthenia | 7 (3.1) |  |  |  | 1 (4.3) | 5 (3.3) | 1 (2.6) | 0 |  |
| Vomiting | 6 (2.6) |  |  |  | 0 | 5 (3.3) | 1 (2.6) | 0 |  |
| Diarrhoea | 5 (2.2) |  |  |  | 0 | 3 (2.0) | 0 | 2 (12.5) |  |
| Gamma-glutamyltransferase increased | 5 (2.2) |  |  |  | 2 (8.7) | 3 (2.0) | 0 | 0 |  |
| Myelosuppression | 5 (2.2) |  |  |  | 0 | 4 (2.6) | 1 (2.6) | 0 |  |
| Nausea | 4 (1.7) |  |  |  | 0 | 3 (2.0) | 1 (2.6) | 0 |  |
| Pneumonitis | 4 (1.7) |  |  |  | 1 (4.3) | 2 (1.3) | 1 (2.6) | 0 |  |
| Electrocardiogram QT prolonged | 3 (1.3) |  |  |  | 0 | 2 (1.3) | 1 (2.6) | 0 |  |
| Stomatitis | 3 (1.3) |  |  |  | 0 | 1 (0.7) | 2 (5.1) | 0 |  |
| Urinary tract infection | 3 (1.3) |  |  |  | 1 (4.3) | 1 (0.7) | 0 | 1 (6.3) |  |
| Decreased appetite | 2 (0.9) |  |  |  | 0 | 2 (1.3) | 0 | 0 |  |
| Hypocalcaemia | 2 (0.9) |  |  |  | 0 | 1 (0.7) | 0 | 1 (6.3) |  |
| Hyponatraemia | 2 (0.9) |  |  |  | 0 | 1 (0.7) | 1 (2.6) | 0 |  |
| Interstitial lung disease | 2 (0.9) |  |  |  | 1 (4.3) | 1 (0.7) | 0 | 0 |  |
| Weight decreased | 2 (0.9) |  |  |  | 0 | 2 (1.3) | 0 | 0 |  |
| Amylase increased | 1 (0.4) |  |  |  | 0 | 1 (0.7) | 0 | 0 |  |
| Aspartate aminotransferase increased | 1 (0.4) |  |  |  | 0 | 1 (0.7) | 0 | 0 |  |
| Blood alkaline phosphatase increased | 1 (0.4) |  |  |  | 0 | 1 (0.7) | 0 | 0 |  |
| Blood creatine phosphokinase MB increased | 1 (0.4) |  |  |  | 0 | 1 (0.7) | 0 | 0 |  |
| Bronchopulmonary aspergillosis | 1 (0.4) |  |  |  | 0 | 0 | 0 | 1 (6.3) |  |
| Fatigue | 1 (0.4) |  |  |  | 0 | 1 (0.7) | 0 | 0 |  |
| Gastrointestinal disorder | 1 (0.4) |  |  |  | 0 | 1 (0.7) | 0 | 0 |  |
| Gastrointestinal haemorrhage | 1 (0.4) |  |  |  | 0 | 0 | 0 | 1 (6.3) |  |
| Gastrointestinal perforation | 1 (0.4) |  |  |  | 0 | 0 | 1 (2.6) | 0 |  |
| Hypoaesthesia | 1 (0.4) |  |  |  | 1 (4.3) | 0 | 0 | 0 |  |
| Infection | 1 (0.4) |  |  |  | 0 | 0 | 1 (2.6) | 0 |  |
| Intestinal obstruction | 1 (0.4) |  |  |  | 0 | 1 (0.7) | 0 | 0 |  |
| Intra-abdominal fluid collection | 1 (0.4) |  |  |  | 0 | 0 | 0 | 1 (6.3) |  |
| Neuralgia | 1 (0.4) |  |  |  | 1 (4.3) | 0 | 0 | 0 |  |
| Neutrophil percentage decreased | 1 (0.4) |  |  |  | 0 | 0 | 0 | 1 (6.3) |  |
| Perihepatic abscess | 1 (0.4) |  |  |  | 0 | 1 (0.7) | 0 | 0 |  |
| Transaminases increased | 1 (0.4) |  |  |  | 0 | 0 | 1 (2.6) | 0 |  |
| Upper respiratory tract infection | 1 (0.4) |  |  |  | 0 | 0 | 1 (2.6) | 0 |  |
| Any treatment-related adverse event leading to treatment discontinuation, n (%) | 20 (8.7) |  |  |  | 3 (13.0) | 10 (6.6) | 2 (5.1) | 5 (31.3) |  |
| Thrombocytopenia | 9 (3.9) |  |  |  | 0 | 4 (2.6) | 2 (5.1) | 3 (18.8) |  |
| Interstitial lung disease | 5 (2.2) |  |  |  | 2 (8.7) | 2 (1.3) | 0 | 1 (6.3) |  |
| Anaemia | 4 (1.7) |  |  |  | 0 | 2 (1.3) | 1 (2.6) | 1 (6.3) |  |
| Leukopenia | 2 (0.9) |  |  |  | 0 | 1 (0.7) | 1 (2.6) | 0 |  |
| Neutropenia | 2 (0.9) |  |  |  | 0 | 1 (0.7) | 1 (2.6) | 0 |  |
| Pneumonitis | 2 (0.9) |  |  |  | 0 | 2 (1.3) | 0 | 0 |  |
| Asthenia | 1 (0.4) |  |  |  | 0 | 1 (0.7) | 0 | 0 |  |
| Bronchopulmonary aspergillosis | 1 (0.4) |  |  |  | 0 | 0 | 0 | 1 (6.3) |  |
| Febrile neutropenia | 1 (0.4) |  |  |  | 0 | 1 (0.7) | 0 | 0 |  |
| Pneumonia | 1 (0.4) |  |  |  | 1 (4.3) | 0 | 0 | 0 |  |

**Supplementary Table 7 Treatment-Related Interstitial Lung Disease (Safety Population)**

|  | | **T-Bren D1D8 Q3W** | **T-Bren D1 Q3W** | | | | | | |
| --- | --- | --- | --- | --- | --- | --- | --- | --- | --- |
|  | **Total** | **1.0 mg/kg** | **2.6 mg/kg** | **3.2 mg/kg** | **3.8 mg/kg** | **4.4 mg/kg** | **5.0 mg/kg** | **5.6 mg/kg** | **6.2 mg/kg** |
| **Phase 1a** |  |  |  |  |  |  |  |  |  |
| **Number of Patients** | **24** | **1** | **3** | **3** | **3** | **3** | **3** | **3** | **5** |
| Any grade | 1 ( 4.2) | 0 | 0 | 0 | 0 | 1 (33.3) | 0 | 0 | 0 |
| Grade 3 | 1 ( 4.2) | 0 | 0 | 0 | 0 | 1 (33.3) | 0 | 0 | 0 |
| **Phase 1b** |  |  |  |  |  |  |  |  |  |
| **Number of Patients** | **229** | **/** | **/** | **/** | **23** | **151** | **39** | **16** | **/** |
| Any grade | 6 ( 2.6) |  |  |  | 2 ( 8.7) | 2 ( 1.3) | 1 ( 2.6) | 1 ( 6.3) |  |
| Grade 1 | 2 ( 0.9) |  |  |  | 0 | 1 ( 0.7) | 1 ( 2.6) | 0 |  |
| Grade 2 | 2 ( 0.9) |  |  |  | 1 ( 4.3) | 0 | 0 | 1 ( 6.3) |  |
| Grade 3 | 2 ( 0.9) |  |  |  | 1 ( 4.3) | 1 ( 0.7) | 0 | 0 |  |

**Supplementary Table 8 Treatment-Related Adverse Events (Sex-Disaggregated Data) (Safety Population)**

| **Preferred Terms** | **Male** | **Female** |
| --- | --- | --- |
| **Phase 1a** |  |  |
| **Number of patients** | **2** | **22** |
| Any treatment-related adverse event, n (%) | 2 ( 100) | 22 ( 100) |
| Treatment-related adverse event in >20% of any group, n (%) |  |  |
| Leukopenia | 2 ( 100) | 20 (90.9) |
| Anaemia | 1 (50.0) | 19 (86.4) |
| Neutropenia | 2 ( 100) | 18 (81.8) |
| Thrombocytopenia | 2 ( 100) | 12 (54.5) |
| Nausea | 0 | 13 (59.1) |
| Asthenia | 1 (50.0) | 10 (45.5) |
| Stomatitis | 1 (50.0) | 10 (45.5) |
| Vomiting | 1 (50.0) | 10 (45.5) |
| Decreased appetite | 1 (50.0) | 8 (36.4) |
| Hypokalaemia | 0 | 9 (40.9) |
| Lymphocyte count decreased | 1 (50.0) | 7 (31.8) |
| Weight decreased | 1 (50.0) | 7 (31.8) |
| Alopecia | 0 | 7 (31.8) |
| Aspartate aminotransferase increased | 0 | 7 (31.8) |
| Gamma-glutamyltransferase increased | 0 | 7 (31.8) |
| Occult blood positive | 0 | 7 (31.8) |
| Alanine aminotransferase increased | 0 | 6 (27.3) |
| Blood alkaline phosphatase increased | 0 | 6 (27.3) |
| Dizziness | 0 | 6 (27.3) |
| Urinary tract infection | 0 | 6 (27.3) |
| Blood lactate dehydrogenase increased | 0 | 5 (22.7) |
| Electrocardiogram T wave abnormal | 0 | 5 (22.7) |
| Headache | 0 | 5 (22.7) |
| Hypocalcaemia | 0 | 5 (22.7) |
| Peripheral sensory neuropathy | 0 | 5 (22.7) |
| Hypoalbuminaemia | 1 (50.0) | 3 (13.6) |
| Pneumonia | 1 (50.0) | 1 (4.5) |
| Blood pressure decreased | 1 (50.0) | 0 |
| Face oedema | 1 (50.0) | 0 |
| Monocyte count decreased | 1 (50.0) | 0 |
| Monocyte percentage decreased | 1 (50.0) | 0 |
| Prothrombin level decreased | 1 (50.0) | 0 |
| **Phase 1b** |  |  |
| **Number of patients** | **31** | **198** |
| Any treatment-related adverse event, n (%) | 31 ( 100) | 197 (99.5) |
| Treatment-related adverse event in >10% of any group, n (%) |  |  |
| Anaemia | 28 (90.3) | 182 (91.9) |
| Leukopenia | 25 (80.6) | 172 (86.9) |
| Neutropenia | 24 (77.4) | 161 (81.3) |
| Thrombocytopenia | 26 (83.9) | 154 (77.8) |
| Nausea | 16 (51.6) | 126 (63.6) |
| Decreased appetite | 17 (54.8) | 94 (47.5) |
| Aspartate aminotransferase increased | 12 (38.7) | 83 (41.9) |
| Vomiting | 8 (25.8) | 80 (40.4) |
| Asthenia | 13 (41.9) | 71 (35.9) |
| Alopecia | 9 (29.0) | 70 (35.4) |
| Lymphocyte count decreased | 11 (35.5) | 64 (32.3) |
| Alanine aminotransferase increased | 9 (29.0) | 63 (31.8) |
| Stomatitis | 5 (16.1) | 61 (30.8) |
| Weight decreased | 10 (32.3) | 56 (28.3) |
| Gamma-glutamyltransferase increased | 7 (22.6) | 57 (28.8) |
| Hypoalbuminaemia | 12 (38.7) | 51 (25.8) |
| Blood alkaline phosphatase increased | 8 (25.8) | 54 (27.3) |
| Constipation | 6 (19.4) | 55 (27.8) |
| Hypokalaemia | 10 (32.3) | 46 (23.2) |
| Diarrhoea | 9 (29.0) | 46 (23.2) |
| Dizziness | 3 (9.7) | 26 (13.1) |
| Abdominal distension | 4 (12.9) | 23 (11.6) |
| Occult blood positive | 2 (6.5) | 25 (12.6) |
| Abdominal pain | 5 (16.1) | 20 (10.1) |
| Electrocardiogram QT prolonged | 3 (9.7) | 22 (11.1) |
| Hyponatraemia | 4 (12.9) | 21 (10.6) |
| Urinary tract infection | 1 (3.2) | 21 (10.6) |
| Hypoproteinaemia | 4 (12.9) | 16 (8.1) |
| Hypocalcaemia | 5 (16.1) | 10 (5.1) |
| Pneumonia | 4 (12.9) | 11 (5.6) |
| Febrile neutropenia | 4 (12.9) | 9 (4.5) |

Supplementary Figure 1 Swimmer Plot for Patients with HER2-Positive Breast Cancer (Modified ITT Population)

**A. Swimmer Plot for Phase 1a. B. Swimmer Plot for Phase 1b**

**
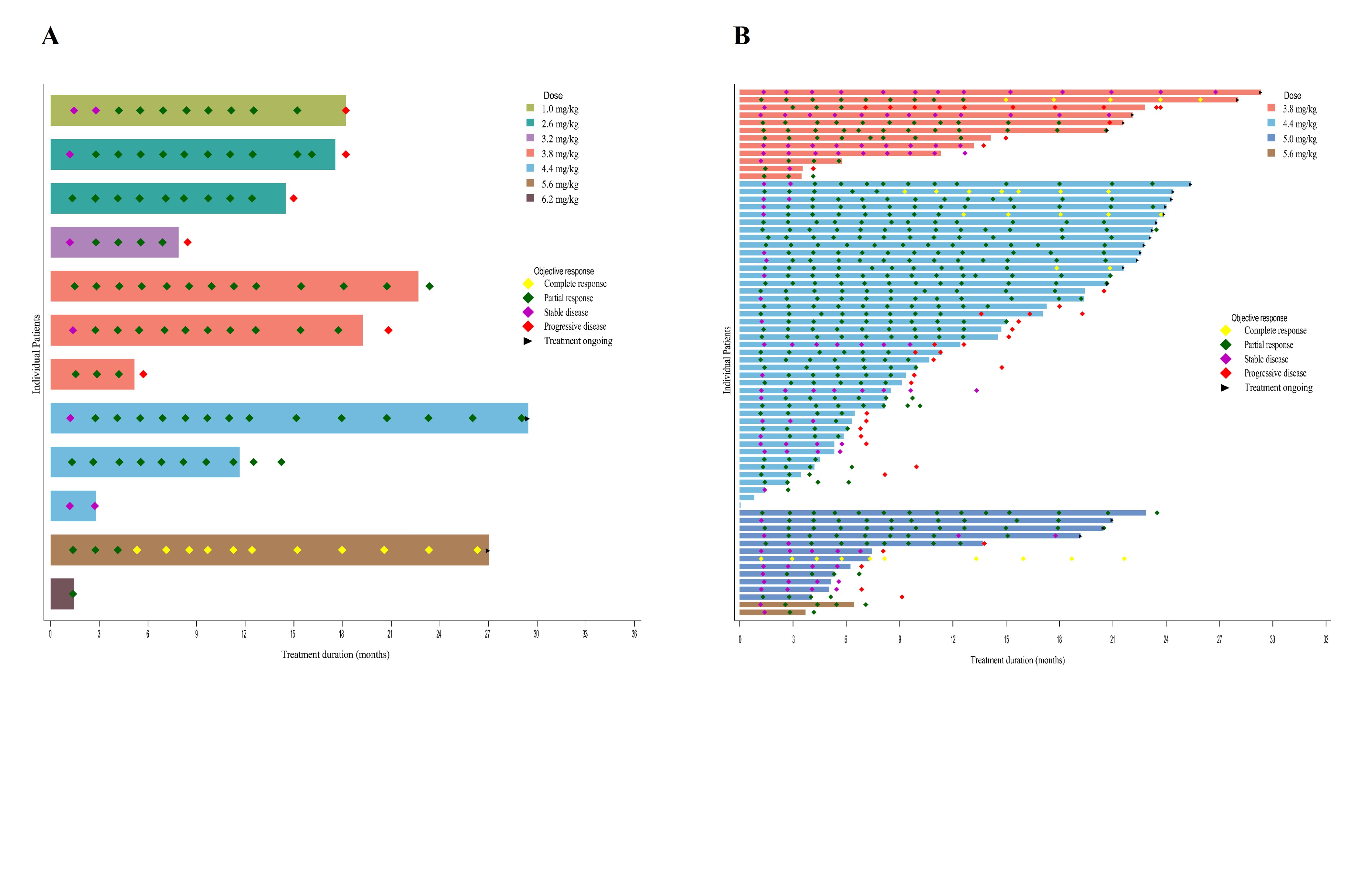
**

**Supplementary Figure 2 Swimmer Plot for Patients with HER2-Negative Breast Cancer (Modified ITT Population)**

**
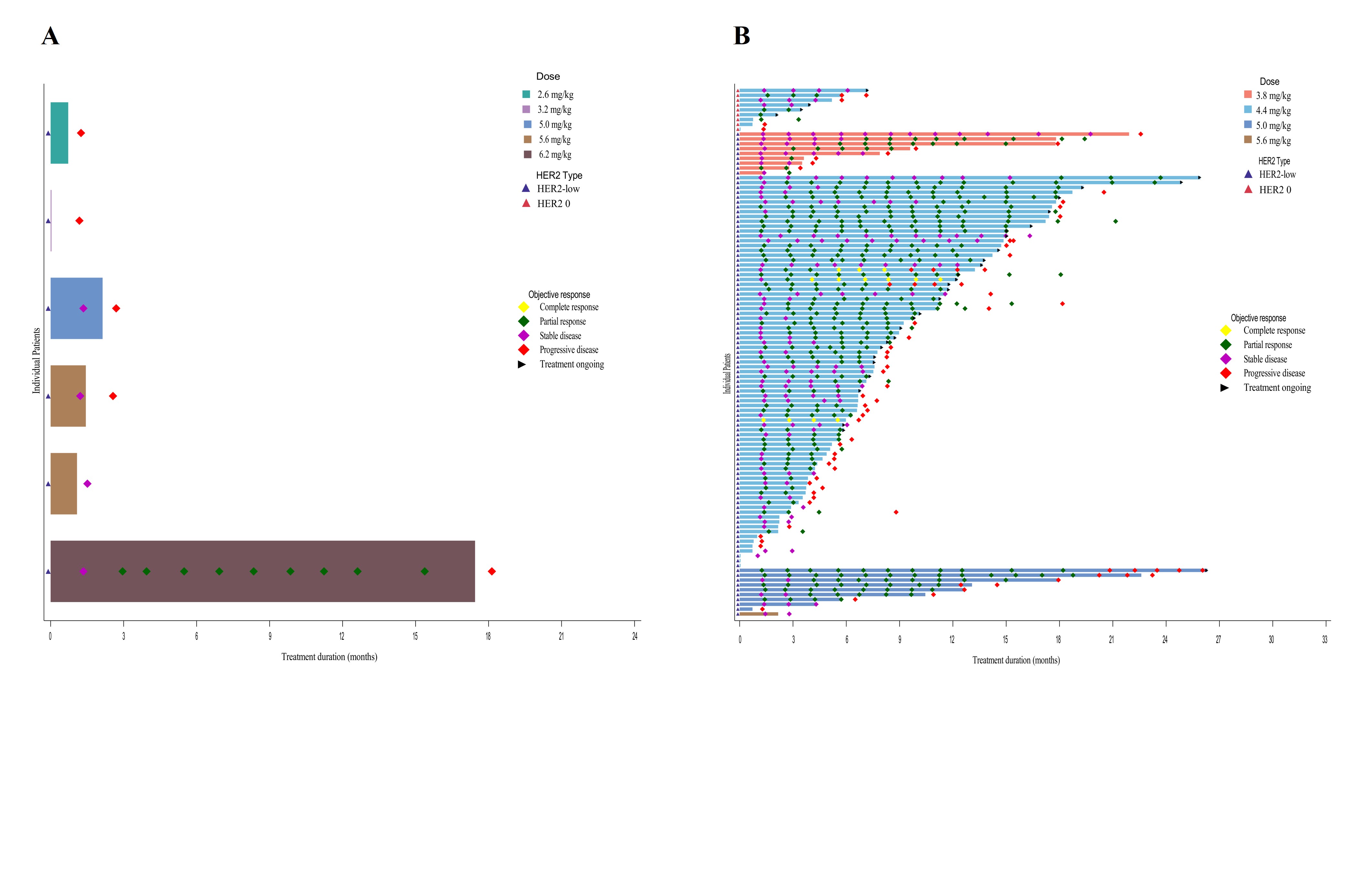
A.****Swimmer Plot for Phase 1a. B.** **Swimmer Plot for Phase 1b**

**Supplementary Figure 3 Swimmer Plot for Patients with Colorectal Cancer, Gastric Cancer, and Lung Cancer (Modified ITT Population)**

**
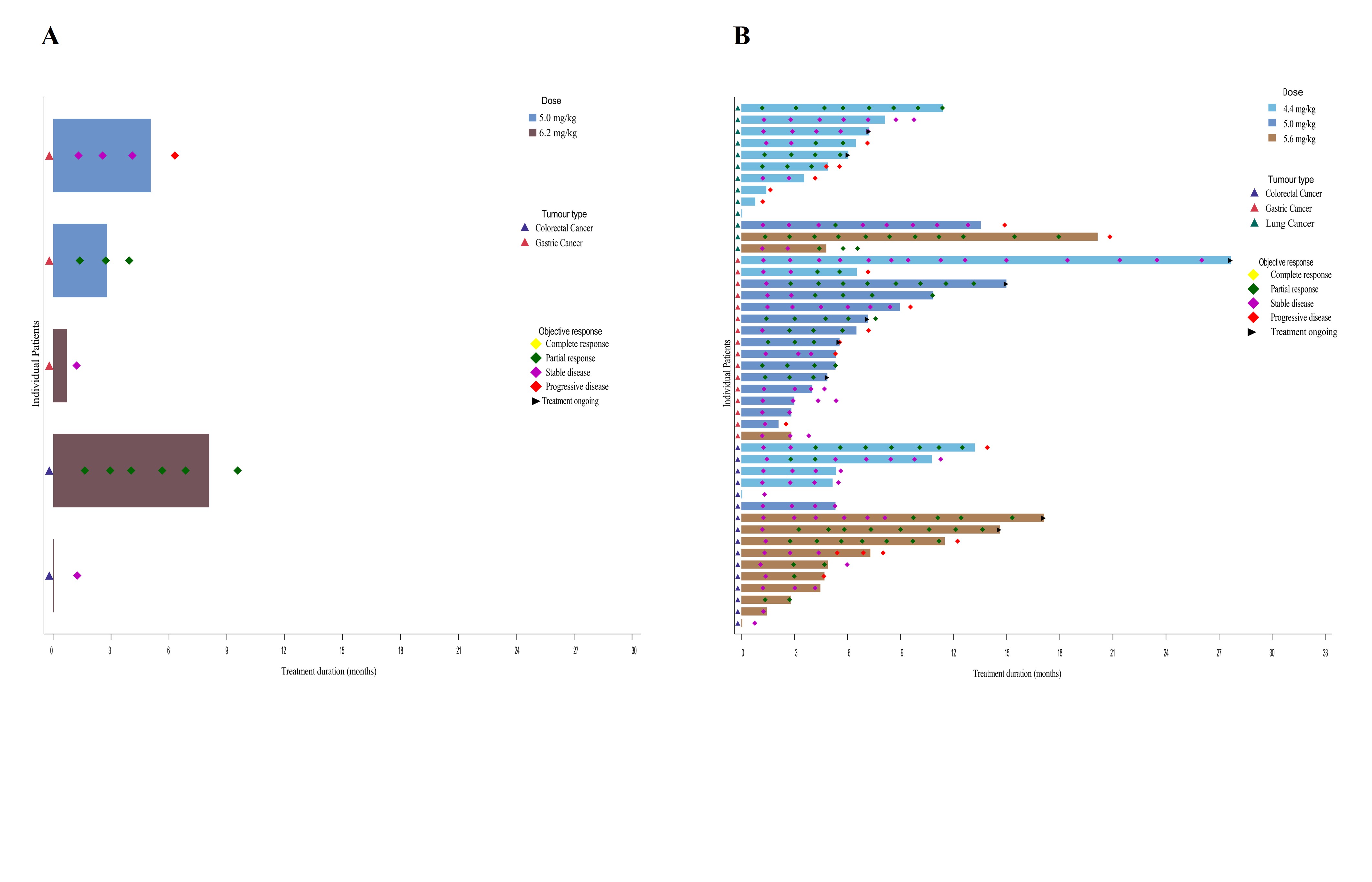
A.Swimmer Plot for Phase 1a. B.** **Swimmer Plot for Phase 1b**

**Supplementary Table 9 Best Overall Response of T-Bren in Solid Tumours other than Breast Cancer (Modified ITT Population)**

| **Variables** |  | **Total^[1]^** |  |  | **Colorectal Cancer** |  |  | **Gastric Cancer** |  | **Lung Cancer^[2]^** |
| --- | --- | --- | --- | --- | --- | --- | --- | --- | --- | --- |
|  | **Total**  **(N = 51)** | **Phase 1a**  **(N=6)** | **Phase 1b**  **(N=45)** | **Total**  **(N = 18)** | **Phase 1a**  **(N=2)** | **Phase 1b**  **(N=16)** | **Total**  **(N = 19)** | **Phase 1a**  **(N=3)** | **Phase 1b**  **(N=16)** | **Total**  **(N = 13)** |
| Best overall response, n (%) |  |  |  |  |  |  |  |  |  |  |
| PR | 25 (49.0) | 2 (33.3) | 23 (51.1) | 9 (50.0) | 1 (50.0) | 8 (50.0) | 9 (47.4) | 1 (33.3) | 8 (50.0) | 7 (53.8) |
| cPR | 23 (45.1) | 2 (33.3) | 21 (46.7) | 8 (44.4) | 1 (50.0) | 7 (43.8) | 9 (47.4) | 1 (33.3) | 8 (50.0) | 6 (46.2) |
| SD | 21 (41.2) | 3 (50.0) | 18 (40.0) | 8 (44.4) | 1 (50.0) | 7 (43.8) | 10 (52.6) | 2 (66.7) | 8 (50.0) | 3 (23.1) |
| PD | 3 (5.9) | 1 (16.7) | 2 ( 4.4) | 0 | 0 | 0 | 0 | 0 | 0 | 2 (15.4) |
| NE^[3]^ | 2 (3.9) | 0 | 2 ( 4.4) | 1 (5.6) | 0 | 1 ( 6.3) | 0 | 0 | 0 | 1 (7.7) |
| ORR, n (% [95%CI]) | 25 (49.0 [34.8-63.4]) | 2 (33.3 [4.3-77.7]) | 23 (51.1 [35.8-66.3]) | 9 (50.0 [26.0-74.0]) | 1 (50.0 [1.3-98.7]) | 8 (50.0 [24.7-75.3]) | 9 (47.4 [24.4-71.1]) | 1 (33.3 [0.8-90.6]) | 8 (50.0 [24.7-75.3]) | 7 (53.8 [25.1-80.8]) |
| ORR confirmed, n (% [95%CI]) | 23 (45.1 [31.1-59.7]) | 2 (33.3 [4.3-77.7]) | 21 (46.7 [31.7-62.1]) | 8 (44.4 [21.5-69.2]) | 1 (50.0 [1.3-98.7]) | 7 (43.8 [19.8-70.1]) | 9 (47.4 [24.4-71.1]) | 1 (33.3 [0.8-90.6]) | 8 (50.0 [24.7-75.3]) | 6 (46.2 [19.2-74.9]) |
| DCR, n (% [95%CI]) | 46 (90.2 [78.6-96.7]) | 5 (83.3 [35.9-99.6]) | 41 (91.1 [78.8-97.5]) | 17 (94.4 [72.7-99.9]) | 2 (100 [15.8-100]) | 15 (93.8 [69.8-99.8]) | 19 (100 [82.4-100]) | 3 (100 [29.2-100]) | 16 (100 [79.4-100]) | 10 (76.9 [46.2-95.0]) |
| CBR, n (% [95%CI]) | 36 (70.6 [56.2-82.5]) | 3 (50.0[11.8-88.2]) | 33 (73.3 [58.1-85.4]) | 14 (77.8 [52.4-93.6]) | 2 (100 [15.8-100]) | 12 (75.0 [47.6-92.7]) | 13 (68.4 [43.4-87.4]) | 1 (33.3 [0.8-90.6]) | 12 (75.0 [47.6-92.7]) | 9 (69.2 [38.6-90.9]) |

CBR, clinical benefit rate; CI, confidence interval; CR, complete response; cPR, confirmed partial response; D, day; DCR, disease control rate; HR, hormone receptor; NE, not evaluable; NR, not reached; PD, progressive disease; PR, partial response; Q3W, every 3 weeks; SD, stable disease Note:

Note:

1. One patient with the diagnosis of adenocarcinoma had a best overall response of progressive disease.

2. All patients in the lung cancer group were from phase 1b.

3. Patients without post-baseline tumor assessment were included.

4. 95% CIs of ORR, DCR and CBR were calculated by using the Clopper-Pearson method.

Supplementary Figure 4 Antitumour Activity of T-Bren in Breast Cancer by HER2 Status (Modified ITT Population)

**
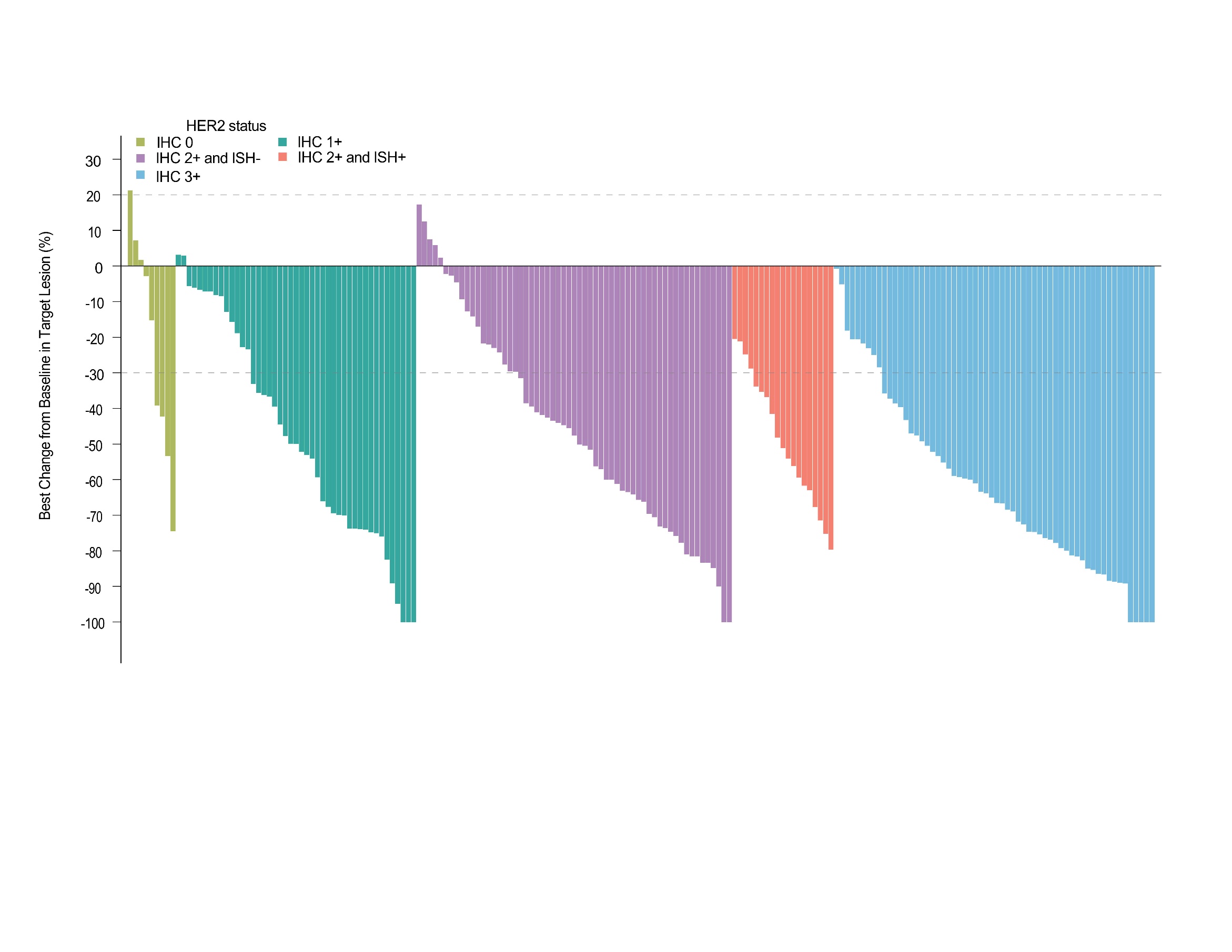
**

Supplementary Figure 5 Antitumour Activity of T-Bren in Gastric Cancer by HER2 Status (Modified ITT Population)

**
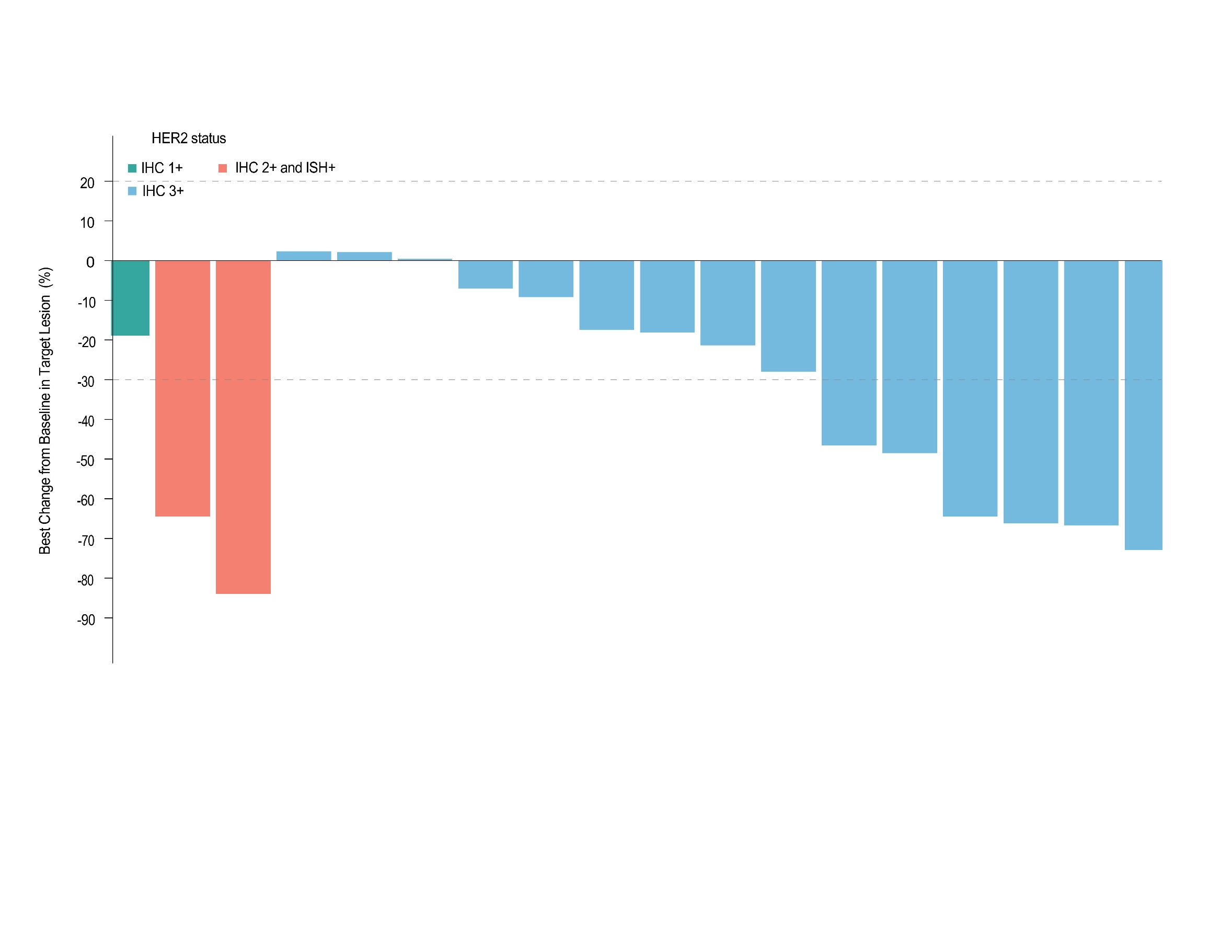
**

Supplementary Figure 6 Antitumour Activity of T-Bren in Colorectal Cancer by HER2 Status (Modified ITT Population)

**
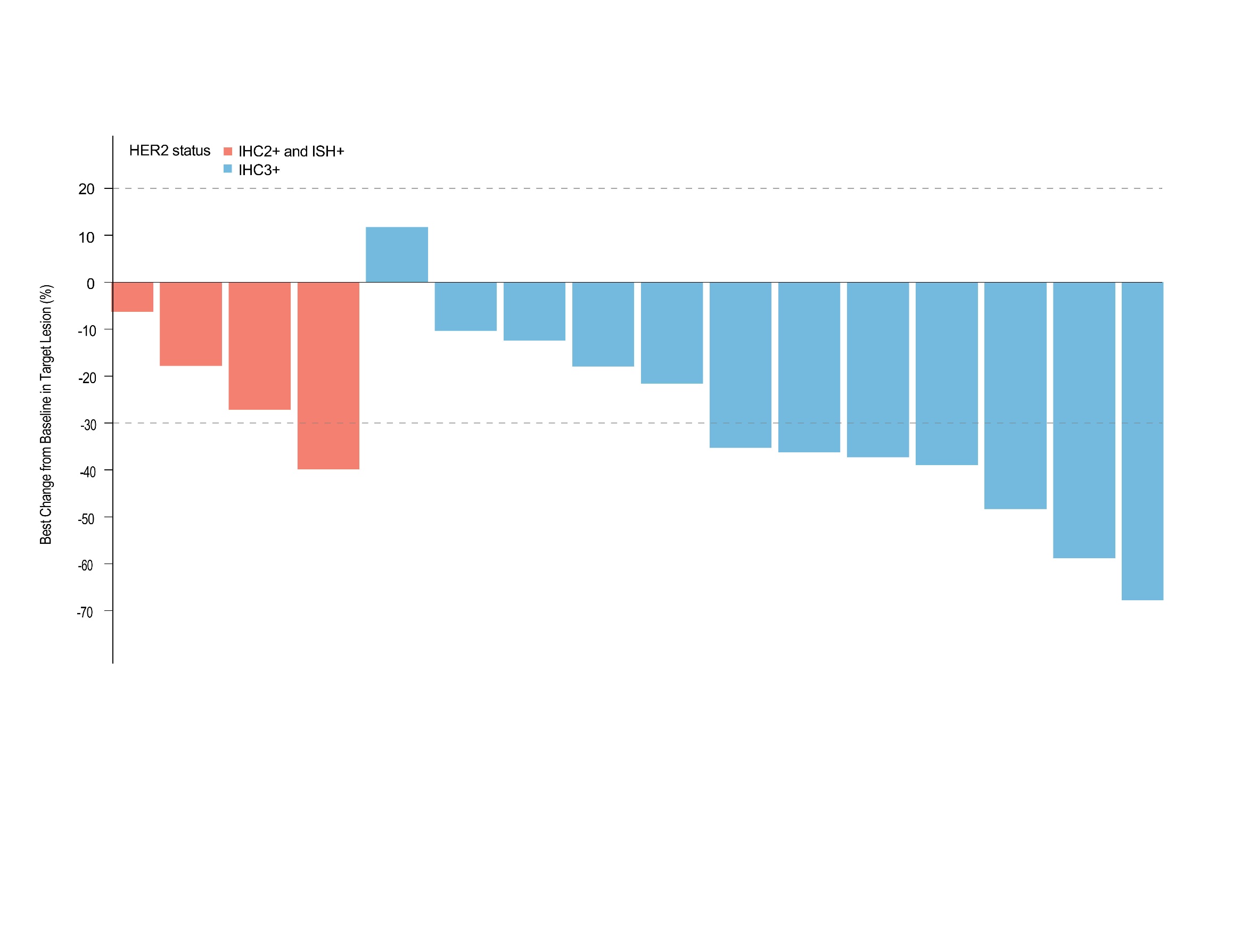
**

Supplementary Figure 7 Swimmer Plot in Breast Cancer by HER2 Status (Modified ITT Population)

**
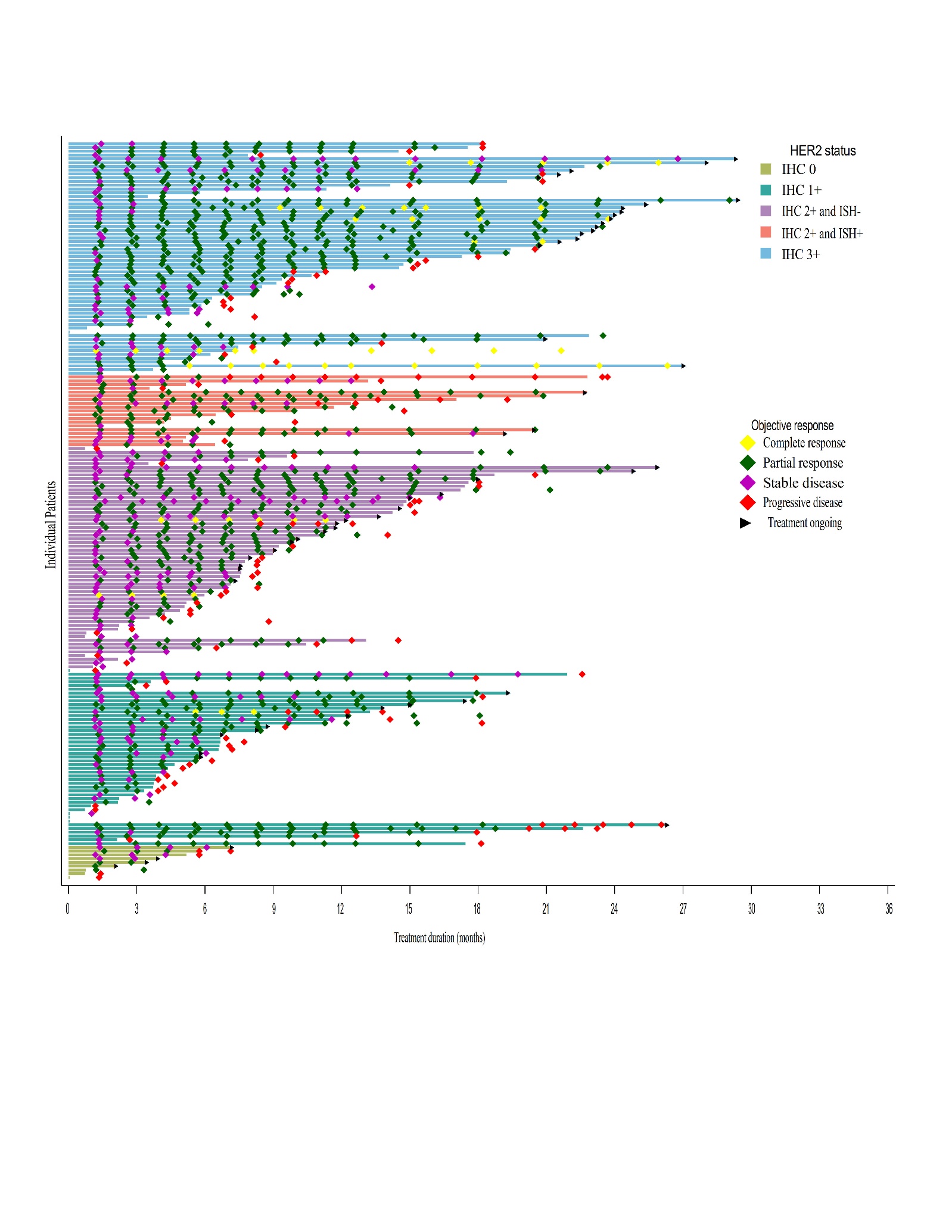
**

Supplementary Figure 8 Swimmer Plot in Gastric Cancer by HER2 Status (Modified ITT Population)

**
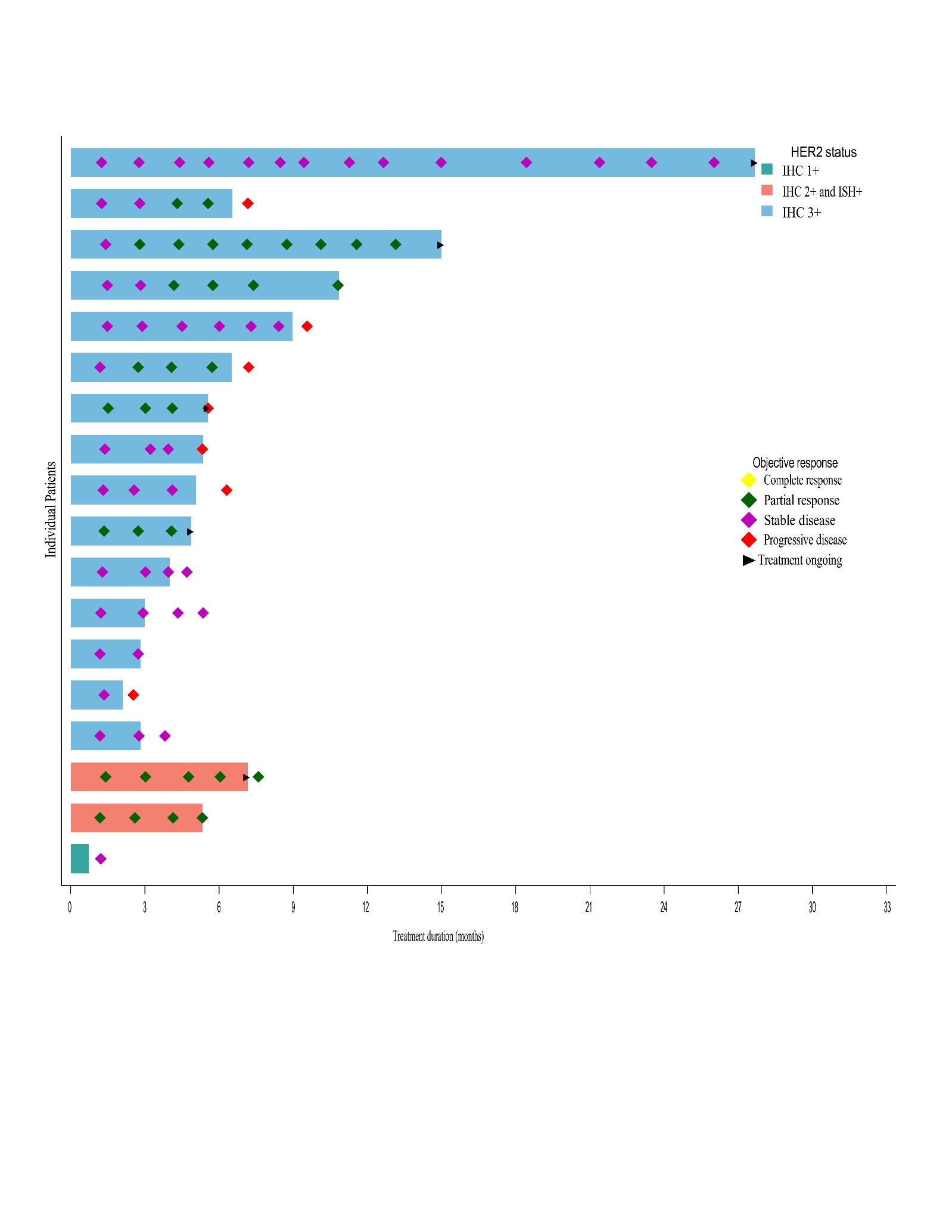
**

Supplementary Figure 9 Swimmer Plot in Colorectal Cancer by HER2 Status (Modified ITT Population)

**
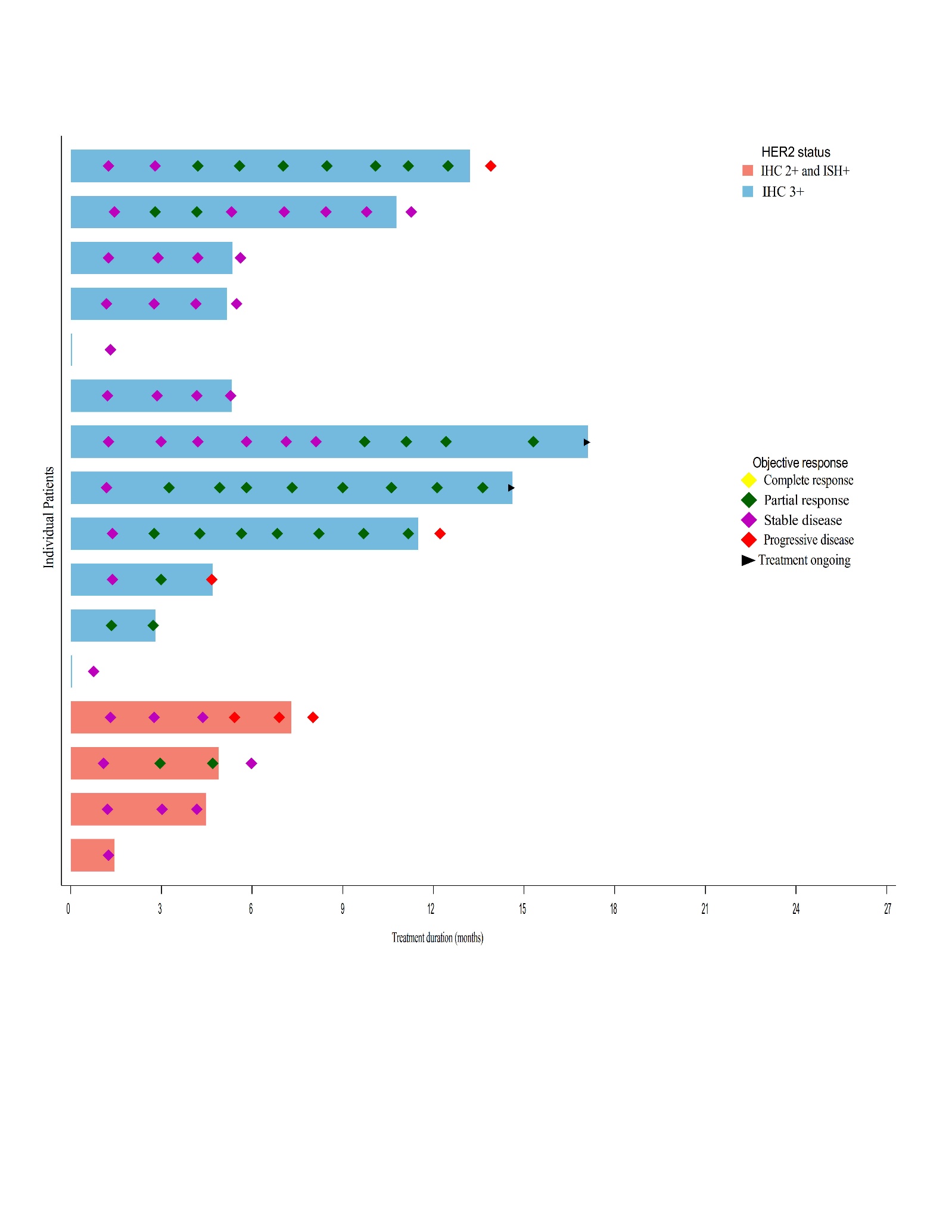
**

Supplementary Figure 10 Objective Response Rates of T-Bren in Breast Cancer, Colorectal Cancer, Gastric Cancer, and Lung Cancer by HER2 IHC Score (Modified ITT Population)


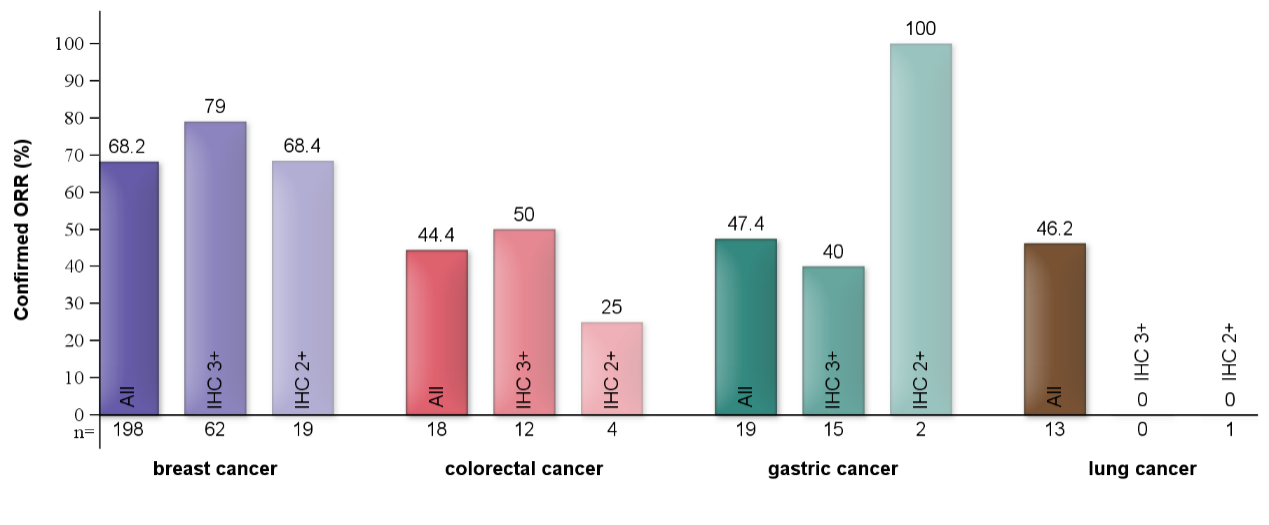


**Supplementary Table 10 Pharmacokinetic Properties of T-Bren and Payload after Single Dose of T-Bren (Dose 1)**

| **PK_Parameter** | **1.0 mg/kg** | **2.6 mg/kg** | **3.2 mg/kg** | **3.8 mg/kg** | **4.4 mg/kg** | **5.0 mg/kg** | **5.6 mg/kg** | **6.2 mg/kg** |
| --- | --- | --- | --- | --- | --- | --- | --- | --- |
| **T-Bren** |  |  |  |  |  |  |  |  |
| n | 1 | 3 | 3 | 7 | 17 | 5 | 3 | 5 |
| C_max_ (ug/mL) | 14.17 (NA) | 45.83 (14.25) | 71.77 (21.11) | 79.89 (19.00) | 104.91 (17.67) | 95.39 (23.24) | 154.37 (19.18) | 117.11 (22.40) |
| AUC_0-504_ (h•μg/mL) | 1043 (NA) | 3903.08 (22.63) | 6013.05 (6.75) | 6633.91 (32.84) | 8065.12 (22.78) | 8415.14 (15.99) | 12991.35 (17.82) | 9716.58 (21.56) |
| T_1/2_ (h) | 54.31 (NA) | 67.66 (23.99) | 71.68 (11.71) | 67.43 (25.05) | 63.56 (15.41) | 75.15 (14.36) | 85.14 (6.38) | 63.75 (22.42) |
| CL (mL/h) | 0.055 (NA) | 0.04 (13.34) | 0.03 (10.77) | 0.03 (32.57) | 0.03 (30.37) | 0.03 (16.47) | 0.03 (13.12) | 0.03 (20.73) |
| V_z_ (mL) | 4.31 (NA) | 3.40 (21.06) | 3.38 (9.49) | 3.29 (22.28) | 2.92 (27.28) | 3.52 (15.45) | 3.33 (9.19) | 3.15 (25.14) |
| T_max_ (h) | 2 (2-2) | 6 (4-8) | 4 (2-6) | 4 (2-8) | 2 (2-8) | 2 (2-8) | 2 (2-8) | 2 (2-8) |
| **Released payload** |  |  |  |  |  |  |  |  |
| n | 1 | 3 | 3 | 7 | 17 | 5 | 2 | 5 |
| C_max_ (ug/mL) | 0.002 (NA) | 0.002 (27.02) | 0.004 (46.92) | 0.005 (64.58) | 0.007 (43.67) | 0.006 (52.64) | 0.008 (20.45) | 0.009 (101.15) |
| AUC_0-504_ (h•μg/mL) | 0.14 (NA) | 0.29 (25.52) | 0.63 (40.07) | 0.45 (50.17) | 0.73 (47.55) | 0.72 (10.84) | 0.77 (4.45) | 0.85 (46.13) |
| T_1/2_ (h) | 97.54 (NA) | 116.99 (36.63) | 156.52 (67.52) | 108.73 (29.52) | 106.93 (26.73) | 163.64 (41.20) | 158.91 (2.61) | 110.53(21.25) |
| CL (mL/h) | 402.48 (NA) | 464.16 (15.14) | 310.98 (28.47) | 497.58 (35.80) | 351.91 (42.02) | 382.30 (6.48) | 470.41 (4.34) | 391.19 (47.02) |
| V_z_ (mL) | 56634.76 (NA) | 78341.00 (38.52) | 70223.68 (59.99) | 78054.97 (48.05) | 54286.46 (40.98) | 90252.73 (40.48) | 107844.52 (6.95) | 62376.69 (69.83) |
| T_max_ (h) | 6 (6-6) | 6 (4-6) | 6 (6-26) | 6 (4-8) | 6 (4-8) | 6 (4-8) | 6 (6-6) | 8 (4-26) |

Note:

Data are geometric mean (%CV) or median (range).

Abbreviations: AUC_0-504_, area under the concentration versus time curve from time zero extrapolated to 504 hours; CL, total body clearance; Cmax, maximum concentration; t1/2, half-life; Tmax, time of observed Cmax; Vz, volume of distribution; NA: not available.

Supplementary Figure 11 Mean (+SD) Serum Concentration versus Time Curve of T-Bren and the Released Payload after Single Dose of T-Bren


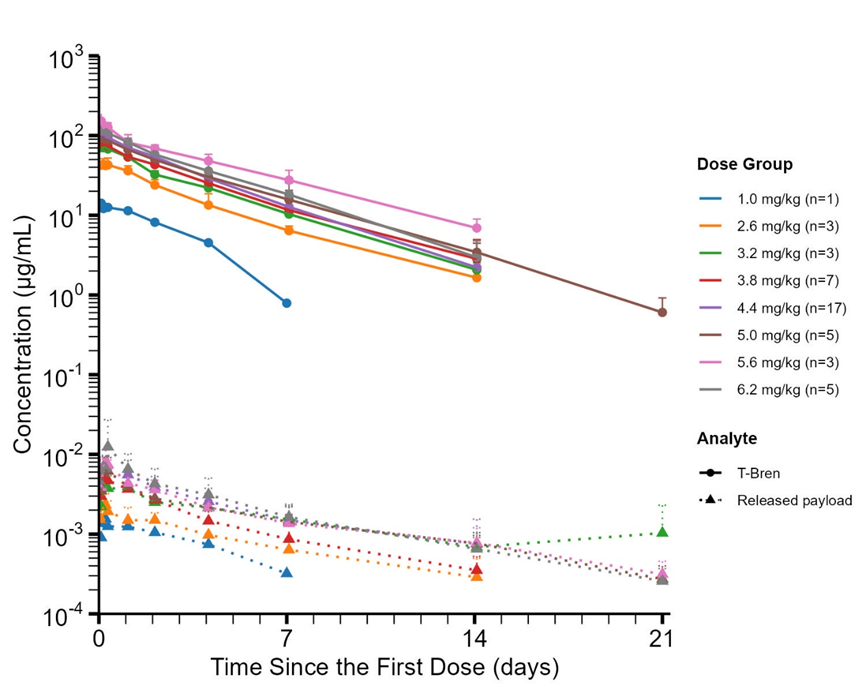

Supplement: Supplementary Material [file mmc1.docx]
