# Supplementary material for: A multi-centre, phase 1a/1b dose escalation and expansion study of the HER2-directed antibody–drug conjugate T-Bren (BL-M07D1) in advanced breast cancer and other solid tumours
Source: eBioMedicine. 2026 Jun 25;129:106351. doi: 10.1016/j.ebiom.2026.106351 (PMC13320388; doi:10.1016/j.ebiom.2026.106351)
Supplement: Clinical Study Protocol [file mmc2.pdf]

A Phase I Clinical Study Evaluating the Safety, Tolerability, Pharmacokinetic Characteristics, and Preliminary Efficacy of BL-M07D1 for Injection in Patients with Locally Advanced or Metastatic HER2-Positive/Negative Breast Cancer and Other Solid Tumors

Clinical study protocol

Leading Site of Clinical Trial: Sun Yat-sen University Sun Yat-sen Memorial Hospital

Leading Investigators: Professor Song, Erwei; Professor Yao, Herui

Study Sponsor: Baili-Bio (Chengdu) Pharmaceutical;

Sichuan Baili Pharmaceutical Co., Ltd.

Protocol version: 2.0

Version date: 7 June 2024

Protocol No.: BL-M07D1-101

CONFIDENTIALITY STATEMENT

Information contained in this document is proprietary to Baili-Bio (Chengdu) Pharmaceutical/Sichuan Baili Pharmaceutical Co., Ltd. The information is provided to you in confidence which is requested under an agreed upon and signed Confidentiality and Disclosure Agreement. Do not give this document or any copy of it or reveal any proprietary information contained in it to any third party (other than those in your organization who are assisting you in this work and are bound by the Confidentiality and Disclosure Agreement) without the prior written permission of an authorized representative of Baili-Bio (Chengdu) Pharmaceutical/Sichuan Baili Pharmaceutical Co., Ltd.

Protocol Signature Page (Sponsor)

I have carefully read and understood the entire content of this study protocol. I will abide by the provisions of the Declaration of Helsinki, China's current Good Clinical Practice for Drug Clinical Trials, and all applicable regulations, assume the sponsor's responsibilities at this research center, and conduct this study in accordance with this protocol.

Name of Sponsor's Responsible Person:

Title of Sponsor's Responsible Person:

Sponsor's Address: No. 139 Baili Road, Chengdu Hi-Tech Industrial Development Zone (Cross-Strait), Wenjiang District, Chengdu

Sponsor's Telephone:028-82631518

Signature of Sponsor's Responsible Person:

Date：

Protocol Signature Page (Investigator)

I have carefully read and understood the entire content of this study protocol. I will keep this protocol confidential, abide by the provisions of the Declaration of Helsinki, China's current Good Clinical Practice for Drug Clinical Trials, and all applicable regulations, assume the investigator's responsibilities at this research center, and conduct this study in accordance with this protocol.

Name of Research Center:

Address of Research Center:

Telephone of Research Center:

Name of Principal Investigator:

Professional Title of Principal Investigator:

Position of Principal Investigator:

Signature of Principal Investigator:

Date:

Protocol Signature Page (Data Management Statistical Unit)

I have carefully read and understood the entire content of this study protocol. I will abide by the provisions of the *Declaration of Helsinki*, China's current *Good Clinical Practice for Drug Clinical Trials*, and all applicable regulations, assume the responsibility for data management and analyses in this study, and conduct the study in accordance with this protocol. I will provide copies of this protocol to all personnel in our company involved in data statistics and report writing for this trial, and discuss the protocol and related materials with them to ensure that they fully understand the protocol and the procedures for conducting data statistics and report writing for this trial.

Name of the Data Management and Statistical Unit:

Name of the Responsible Person of the Data Management and Statistical Unit (in block letters):

Signature of the Responsible Person of the Data Management and Statistical Unit:

Date:

TABLE OF CONTENTS

PROTOCOL SIGNATURE PAGE (SPONSOR) ... 2

PROTOCOL SIGNATURE PAGE (INVESTIGATOR) ... 3

PROTOCOL SIGNATURE PAGE (DATA MANAGEMENT STATISTICAL UNIT) ... 4

PROTOCOL SYNOPSIS ... 9

TABLE1. PHASE I RESEARCH SCHEDULE ... 33

LIST OF ABBREVIATIONS ... 41

1. BACKGROUND ... 43

1.1. INTRODUCTION OF INVESTIGATIONAL DRUG ... 44

1.1.1. Drug name ... 44

1.1.2. Chemical Structure and Physicochemical Properties ... 45

1.2. SUMMARY OF NONCLINICAL RESEARCH ... 45

1.2.1. Safety Research Results of Pre-clinical study and risk warnings ... 45

1.2.2. Properties of pre-clinical pharmacodynamics ... 47

1.2.3. Properties of pre-clinical pharmacokinetics ... 49

2. OVERALL STUDY PLAN ... 49

2.1. STUDY PLAN OF CLINICAL TRIAL ... 49

2.1.1. Phase I Clinical trial ... 50

2.1.2. Phase II Clinical trial ... 51

2.1.3. Phase III Clinical trial ... 52

2.2. EVALUATION METHODS OF DRUG SAFETY ... 53

3. STUDY CONTENTS ... 55

3.1. STUDY OBJECTIVES ... 55

3.1.1. Dose Escalation (Ia) ... 55

3.1.2. Enrollment Expansion (Ib) ... 56

3.2. STUDY ENDPOINTS ... 57

3.2.1. Dose Escalation (Ia) ... 57

3.2.2. Enrollment Expansion (Ib) ... 57

3.3. STUDY STAGE ... 58

3.4. STUDY DESIGN ... 58

3.4.1. Overall Design of Study ... 58

3.4.2. Selection Basis of Control Group ... 64

3.4.3. Dose Escalation Method ... 64

3.4.4. Administration regimen and definition of DLT and MTD ... 67

3.4.5. Supplementary Subject ... 77

4. TARGET SUBJECT ... 77

4.1. INCLUSION CRITERIA 78
4.2. EXCLUSION CRITERIA 79
4.3. STUDY DURATION 82
4.4. EARLY TERMINATION OR SUSPENSION ENROLLMENT OF STUDY 82
4.5. PATIENT WITHDRAWAL FROM TREATMENT CRITERIA 83
4.6. DEFINITION OF PATIENT COMPLETION OF THE STUDY 83

5. INVESTIGATIONAL PRODUCT 84

5.1. INFORMATION ON INVESTIGATIONAL PRODUCT 84
5.2. PREPARATION OF INVESTIGATIONAL PRODUCT 85
5.2.1. Preparation of BL-M07D1 for Injection 85
5.2.2. Recommended of Infusion Rate 86
5.2.3. Precautions 86
5.3. SUPPLY, PACKAGING, LABELLING AND STORAGE OF INVESTIGATIONAL PRODUCT 87
5.4. RECEPTION, DISTRIBUTION AND CUSTODY OF THE INVESTIGATIONAL DRUG 87
5.5. RANDOMNESS AND BLINDNESS 88
5.6. CONCOMITANT MEDICATIONS BEFORE AND DURING THE STUDY 88
5.6.1. Drugs not to be Used during the Study 88
5.6.2. Medications Allowed to be Used During the Study 88

6. STUDY PROCEDURES 103

6.1 PHASE IA STUDY PROCEDURES 103
6.1.1 Visiting 1: Screening (Day-28 ~ Day-1) 103
6.1.2 Visiting 2 (Cycle 1 Day 1,administration) 104
6.1.3 Visiting 3,4 (Cycle 1 Day 2,3) 105
6.1.4 Visiting 5-8 (Cycle 1 Day 8, 15 and 22) 105
6.1.6 Subsequent Cycles 107
6.1.7 End of treatment visit 108
6.1.8 Safety Follow-up 109
6.1.9 Follow-up Visit 109
6.2 PHASE IB STUDY PROCEDURES 110

7 SAFETY AND TOLERANCE ASSESSMENTS 110

7.1 VITAL SIGNS AND BODY WEIGHT 110
7.2 PHYSICAL EXAMINATION 111
7.3 LABORATORY EXAMINATION 111
7.4 ECG 112
7.5 ECHOCARDIOGRAM 112

8 PHARMACOKINETIC STUDY 112

8.1 PHASE IA PHARMACOKINETIC STUDY 112
8.2 PHASE IB PHARMACOKINETIC STUDY 116
8.3 COLLECTION, PROCESSING AND STORAGE OF BIOLOGICAL SAMPLES 116
8.4 COLD CHAIN SYSTEM FOR BIOLOGICAL SAMPLE TRANSPORT 117

9 IMMUNOGENICITY EVALUATION 117

10 EFFICACY EVALUATION 117

10.1 TUMOR EVALUATION METHOD AND TIME 117
10.2 EFFICACY EVALUATION CRITERIA 118

11. ADVERSE EVENTS 119

11.1. THE DEFINITION AND REPORT OF ADVERSE EVENT 119
11.2. ADVERSE EVENT RECORD AND FOLLOW-UP 121
11.3. CRITERIA FOR ADVERSE EVENT SEVERITY EVALUATION 121
11.4. DETERMINATION OF CAUSALITY BETWEEN ADVERSE EVENTS AND THE INVESTIGATIONAL DRUG 122
11.5. SERIOUS ADVERSE EVENT 124
11.5.1. Definition of Serious Adverse Event 124
11.5.2. Reporting and follow-up of serious adverse events 126
11.5.3. Pregnancy 127

12. STATISTICAL ANALYSIS 128

12.1. DETERMINATION OF SAMPLE SIZE 128
12.2. ANALYSIS SET 128
12.2.1. Definition of analysis set 128
12.2.2. Determination of analysis set 129
12.3. STATISTICAL METHODS 130
12.3.1. General Statistical Considerations 130
12.3.2. Demographic Analysis 131
12.3.3. Dose-Limiting Toxicity Evaluation 131
12.3.4. Safety Analysis 131
12.3.5. Pharmacokinetic Analysis 132
12.3.6. Efficacy Analysis 132

13. DEAL WITH ABNORMAL SITUATION DURING STUDY 133

13.1. RISK ASSESSMENT AND RISK MANAGEMENT PLAN 133
13.1.1. Risks associated with study drug 134
13.1.2. Overall plan for safety management 135

14. PROTOCOL APPROVAL AND AMENDMENT 140

15. DATA MANAGEMENT 141

15.1. REQUIREMENTS FOR THE INVESTIGATOR TO FILL IN DATA 141
15.2. DATA MONITORING 142
15.3. DATABASE ESTABLISHMENT AND DATA ENTRY 142
15.4. PROTECTION OF CONFIDENTIAL DATA 143
15.5. DATA LOCK AND HANDOVER 143

16. QUALITY ASSURANCE 144

16.1. QUALITY ASSURANCE OF CLINICAL STUDY PROCESS 144
16.1.1. Monitoring *144*
16.1.2. Data Verification *144*
16.1.3. Audit and Inspection *145*
16.1.4. Personnel Training *145*
16.2. QUALITY ASSURANCE OF SAMPLE TESTING PROCESS 145
17. ETHICAL CRITERION AND INFORMED CONSENT FORM 145
17.1. ETHICAL CRITERION 145
17.2. INFORMED CONSENT FORM 146
18. RESEARCH REPORTS 146
18.1. TOLERABILITY AND PRELIMINARY EFFICACY EVALUATION REPORT 146
18.2. PHARMACOKINETIC STUDY REPORT 147
19. DATA RETENTION 147
20. LIABILITY AND INSURANCE 148
REFERENCE 148

Protocol Synopsis

**Protocol Number:**	BL-M07D1-101
**Study Title:**	A Phase I Clinical Study Evaluating the Safety, Tolerability, Pharmacokinetic Characteristics, and Preliminary Efficacy of BL-M07D1 for Injection in Patients with Locally Advanced or Metastatic HER2-Positive/Negative Breast Cancer and Other Solid Tumors
**Protocol Version and Date:**	Version 2.0/7 June 2024
**Sponsor:**	Baili-Bio (Chengdu) Pharmaceutical Co., Ltd.<br>Sichuan Baili Pharmaceutical Co., Ltd.
**Investigational Product:**	BL-M07D1 for injection (BL-M07D1)
**Study Sites and Planned Sample Size:**	The clinical study has two phases:<br>**Dose escalation (Phase Ia)**: The study is designed by accelerated titration combined with i3+3, recruiting about 28 patients with HER2-positive/low-expression breast cancer and other solid tumors (to be determined based on study progress); the study is planned to be conducted at 1 to 10 study centers.<br>**Enrollment Expansion (Phase Ib):** It is divided into two parts: the dose extension cohort and the indications extension cohort. One or more doses will be selected for dose extension cohort to further assess the safety and efficacy of BL-M07D1 at different doses. Multiple tumor types will be selected for indications extension cohort to further assess the safety and efficacy of BL-M07D1 at different indications. The actual enrollment for each indication will be adjusted based on efficacy and safety and is planned to be carried out at 10 to 20 centers.

**Target Population:**	Patients with locally advanced or metastatic HER2-positive/negative breast cancer and other solid tumors who have failed standard treatment or have no access to standard treatment.
**Study Objectives:**	**1. Dose escalation (Phase Ia)**<br>**1)Primary Objective**<br>• Observing the safety and tolerability of BL-M07D1 in patients with locally advanced or metastatic HER2-positive/low-expression breast cancer and other solid tumors to determine the maximum tolerance dose (MTD) and dose-limiting toxicity (DLT) of BL-M07D1.<br>**2)Secondary Objectives**<br>• To assess the pharmacokinetic (PK) profile of BL-M07D1 in patients with locally advanced or metastatic HER2-positive/low-expression breast cancer and other solid tumors.<br>• To assess the incidence of immunogenicity of BL-M07D1 in patients with locally advanced or metastatic HER2-positive/low-expression breast cancer and other solid tumors.<br>**3)Exploratory Objective:**<br>• Detecting HER2 protein expression in tumor tissues to explore the correlation with efficacy outcomes of BL-M07D1.<br>**2. Enrollment Expansion (Ib)**<br>**1)Primary Objective**<br>• Further observing the safety and tolerability of BL-M07D1 at the recommended phase Ia dose to determine the recommended phase II dose (RP2D).<br>**2)Secondary Objectives**<br>• To evaluate the preliminary efficacy of BL-M07D1 in patients with locally advanced or metastatic HER2-positive/negative breast cancer and other solid tumors.

	• To further assess the PK of BL-M07D1 in patients with locally advanced or metastatic HER2-positive/negative breast cancer and other solid tumors.<br>• To assess the incidence of immunogenicity of BL-M07D1 in patients with locally advanced or metastatic HER2-positive/negative breast cancer and other solid tumors.<br>**3)Exploratory Objective:**<br>• According to the results of phase Ia, biomarkers will be optimized to further study the correlation between selected biomarkers and initial efficacy.
**Study Endpoints:**	**1. Dose escalation (Ia)**<br>**1)Primary Endpoint**<br>• Dose-limiting toxicity (DLT), maximum tolerance dose (MTD).<br>**2)Secondary Endpoints**<br>• Type, frequency and severity of treatment-emergent adverse event (TEAE) during the treatment of BL-M07D1；<br>• Pharmacokinetic (PK) parameters: C_{max}, T_{max}, $T_{1/2}$, AUC_{0-t}, CL, C_{trough} etc.<br>• Immunogenicity: incidence of anti-BL-M07D1 antibody；<br>**3)Exploratory Endpoints**<br>• HER2 protein expression in tumor tissues as biomarkers for predicting BL-M07D1 efficacy.<br>• Neutralizing activity of anti-BL-M07D1 antibody.<br>**2. Enrollment expansion (Ib)**<br>**1)Primary Endpoint**<br>• Recommended phase II dose (RP2D)<br>**2)Secondary Endpoints**<br>• Type, frequency and severity of treatment-emergent adverse event (TEAE) during the treatment of BL-M07D1；

	• Objective response rate (ORR), disease control rate (DCR), duration of response (DOR); • Pharmacokinetic (PK) parameters: C_{max}, T_{max}, $T_{1/2}$, $AUC_{0\text{-}t}$, CL, C_{trough}, etc; • Immunogenicity: incidence of anti-BL-M07D1 antibody. **3)Exploratory Endpoints** • Progression-free survival (PFS), overall survival (OS); • HER2 protein expression in tumor tissues as biomarkers for predicting BL-M07D1 efficacy. • Neutralizing activity of anti-BL-M07D1 antibody.
**Study Design:**	This study is an open-label, multicenter, dose-escalation, and expansion non-randomized Phase I clinical trial. It aims to evaluate the safety, tolerability, pharmacokinetic (PK) profile, and preliminary efficacy of BL-M07D1 for injection in patients with locally advanced or metastatic HER2-positive/negative breast cancer and other solid tumors. The study is divided into two phases: the dose-escalation phase (Phase Ia) and the enrollment expansion phase (Phase Ib). **1. Dose escalation (Ia)** **The original dose-escalation plan:** In this phase, accelerated titration and i3+3 combined design were used to recruit about 28 patients with locally advanced or metastatic HER2-positive/low-expression breast cancer and other solid tumors (determined according to the progress of the study), and it is planned to be carried out in 1 to 10 research centers. The initial preset dose groups of BL-M07D1 were 1.0 mg/kg, 3.0 mg/kg, 4.0 mg/kg, 5.0 mg/kg, and 6.0 mg/kg. Starting from 3.0 mg/kg, the default increment for dose escalation is 1.0 mg/kg. The dose escalation increment will be adjusted as follows (see the Dose Escalation Adjustment Table for details):

	① Beginning from 3.0 mg/kg, if a dose-limiting toxicity (DLT) occurs for the first time, the adjusted increment for subsequent dose escalations will be modified to 0.5 mg/kg. If the first DLT occurs at 6.0 mg/kg, no adjustment will be made.<br>② If multiple DLTs occur at the same dose, or if DLTs occur at subsequent different doses, the default dose escalation increment will no longer be adjusted. However, the increment may be adjusted or intermediate doses may be added after consultation with the investigator(s).<br>Dose adjustment table during dose escalation:

The dose group in which the first DLT occurred	Dose groups
3.0 mg/kg	1.0, 3.0, 3.5, 4.0, 4.5, 5.0, 5.5, 6.0 mg/kg
4.0 mg/kg	1.0, 3.0, 4.0, 4.5, 5.0, 5.5, 6.0 mg/kg
5.0 mg/kg	1.0, 3.0, 4.0, 5.0, 5.5, 6.0 mg/kg
6.0 mg/kg	1.0, 3.0, 4.0, 5.0, 6.0 mg/kg

In this study, administration is scheduled on Day 1 (D1) and Day 8 (D8) of each 3-week cycle, with the first cycle serving as the DLT observation period. During dose escalation under this administration schedule, for dose groups that have completed the DLT observation and demonstrated favorable safety profile, further exploration may proceed at twice the dose or a dose mutually determined by consultation with investigators, following a once-every-3-week (Q3W) schedule in 3-week cycles. For example, after completing the exploration of 3.0 mg/kg administered on D1 and D8 of each 3-week cycle (with a total Q3W dose of 6.0 mg/kg), exploration of 6.0 mg/kg administered on D1 of each Q3W cycle may be initiated. The number of subjects enrolled for exploring administration frequency will not be counted in the sample size for dose escalation.

	If dose reduction is required in accordance with escalation rules or due to other safety considerations, adjustments will be made as follows, or alternative doses may be selected upon discussion and agreement between the sponsor and investigators:<br>① If the interval between the intolerable dose (DLT uncleared) and the highest tolerated dose that has completed escalation (DLT cleared) is 2.0 mg/kg, the dose will be reduced to [highest tolerated dose that completed escalation + 1.5 mg/kg]. For example, if 1.0 mg/kg is tolerated but 3.0 mg/kg is intolerable, the dose will be reduced to 2.5 mg/kg.<br>② If the interval between the intolerable dose (DLT uncleared) and the highest tolerated dose that completed escalation (DLT cleared) is 1.5 mg/kg, the dose will be reduced to [highest tolerated dose that completed escalation + 1.0 mg/kg]. For example, if 1.0 mg/kg is tolerated but 2.5 mg/kg is intolerable, the dose will be reduced to 2.0 mg/kg.<br>③ If the interval between the intolerable dose (DLT uncleared) and the highest tolerated dose that completed escalation (DLT cleared) is 1.0 mg/kg, the dose will be reduced to [highest dose that completed escalation + 0.5 mg/kg]. For example, if 3.0 mg/kg is tolerated but 4.0 mg/kg is intolerable, the dose will be reduced to 3.5 mg/kg.<br><br>**The current dose-escalation plan:**<br>Considering the comprehensive balance between subject safety and benefits, after completing the dose escalation of the 1.0 mg/kg dose group (i.e., Cohort A) — administered on Day 1 (D1) and Day 8 (D8) with a once-every-3-week (Q3W) schedule — the subsequent escalation doses will be reduced, and the escalation protocol will be adjusted to Cohort B. Cohort B will use the following doses: 2.6 mg/kg, 3.2 mg/kg, 3.8 mg/kg, 4.4 mg/kg, 5.0 mg/kg, 5.6 mg/kg, 6.2 mg/kg, 6.8 mg/kg, and 7.4 mg/kg, administered on D1 of each 3-week cycle.

If dose adjustment is required in accordance with escalation rules or due to other safety considerations, the dose may be reduced to [highest dose that has completed escalation + 0.3 mg/kg]. For example, if 3.8 mg/kg is tolerated but 4.4 mg/kg is not, the dose will be reduced to 4.1 mg/kg. Alternatively, after discussion between investigators and the sponsor, other appropriate doses or administration schedules may be selected for the ongoing dose escalation study.

Dose Escalation Cohort Design

Based on the starting dose setting of this study drug, the effective dose of the marketed competitor drug DS-8201, and the tolerability of patients in the previous dose escalation phase, the dose escalation cohorts of this study drug are designed with full consideration of the patient's benefit-risk ratio, as follows:

Cohort A: D1, D8, Q3W<br>Dose (mg/kg)	Cohort B: D1, Q3W<br>Dose (mg/kg)
1.0	-
-	2.6
-	3.2
-	3.8
-	4.4
-	5.0
-	5.6
-	6.2
-	6.8
-	7.4

Before each administration of BL-M07D1, a complete blood count (CBC) test is required. Neutrophils and platelets must meet the following criteria before each subsequent administration (for cohort B, only D1 dosing):

	Neutrophils≥	Platelets≥

D 0 or 1	1.5×10^9 /L	90×10^9 / L
D 7 or 8	1.5×10^9 / L	75×10^9 / L

In subsequent cycles, patients must meet the following criteria before each dose administration:

	Neutrophils≥	Platelets≥
D 0 or 1[1]	1.5×10^9 /L	75×10^9 / L
D 7 or 8[1]	1.0×10^9 / L	75×10^9 / L

Note1: In the Phase Ib expansion period, except for the first dose administration in Cycle 1, this standard shall apply to each subsequent dose administration.

Dose escalation is performed by a combination of accelerated titration[1] and i3 +3[2] in a cohort of 2 to 4 subjects. Accelerated titration design will be used for the first dose group (only 1 subject will be enrolled):

a. If DLT or two grade≥2 drug-related AE (except infusion related reactions) are observed in the accelerated titration dose group, an additional 2 to 3 subjects will be added to the current dose level, and the subsequent study design will automatically be changed to an i3 +3 design of 2 to 4 subjects;
b. If no DLT is observed in the accelerated titration dose groups and the number of grade≥2 drug-related AE is less than two, the i3+3 design of 2 to 4 subjects will be used for dose escalation starting from the second dose group.
c. If safety events are observed in the accelerated titration dose group, the investigator evaluates that it is necessary to add subjects and continue to observe drug toxicity at the current dose level.

The i3+3 design uses a Bayesian statistical framework and Beta-Bernoulli model to pre-calculate dose-increasing decision rules. The parameters of the i3 +3 design in this study will adopt a target DLT

rate of 28% and an equivalent interval of 23% ~ 33% (i.e., target DLT rate ±5%).

During the dose-escalation phase, dosing will be spaced at least 24 hours between enrolled subjects to allow for an initial assessment of tolerability and toxicity.

Stop dose escalation if any of the following conditions are met:

• Maximum sample size (approximately 55 cases in total);

• A sample size of ≥ 6 subjects treated at the same dose and the dose is determined to be MTD.

Note: Estimates of the maximum sample size are based on the number of subjects who are assumed to have climbed to the highest dose group and terminated the climb after full exposure to toxicity.

Based on the i3+3 design, the detailed rules of dose increase/decrease are shown in Table 1 and Table 2 below.

Table 1 i3 +3 dose increase/decrease rule (according to 3-subject cohort)

Total number of patients treated at current dose	3	6	9	12
Increase dose if number of DLT ≤	0	1	2	2
Dose reduction if number of DLT ≥	2	3	4	4
Terminate administration if number of DLT ≥	3	4	5	6

Note:

1. Termination of dosing represents the withdrawal of the current dose and all higher doses from the study for the purpose of protecting the subject. When termination occurs, the dose is automatically lowered to the next dose. If termination occurs at the lowest dose, the entire trial is stopped and MTD cannot be confirmed.

2. If no dose increase, dose decrease, or dose termination occurs, maintain the dose.

3. If the current dose is the minimum dose but the dose reduction is indicated in Table 1, the test is stopped and MTD cannot be confirmed. If the current dose is the maximum dose, but the table indicates an increase in the dose, subsequent patients will be maintained at the current maximum dose until the discontinuation condition is met.

Table 2 i3 +3 detailed dose increase/decrease rule

Number of Patients

Number of DLTs	1	2	3	4	5	6	7	8	9	10	11	12
0	E	E	E	E	E	E	E	E	E	E	E	E
1	S	S	S	S	E	E	E	E	E	E	E	E
2		DU	D	D	S	S	S	S	E	E	E	E
3			DU	DU	D	D	D	D	S	S	S	S
4				DU	DU	DU	DU	D	D	D	D	D
5					DU	DU	DU	DU	DU	D	D	D
6						DU	DU	DU	DU	DU	DU	DU
7							DU	DU	DU	DU	DU	DU
8								DU	DU	DU	DU	DU
9									DU	DU	DU	DU
10										DU	DU	DU
11											DU	DU
12												DU

Note 1: Target DLT rates pT=0.28, ε1=0.05, ε2=0.05, equivalent range 0.23~ 0.33.

Note 2: E Dose increase; S Maintain current dose; D Reduce to a previous dose; DU Lower to previous dose and cancel all doses above. Dose increases will only be triggered if the number of subjects in the group is ≥3, otherwise the current dose will be maintained.

After the dose escalation stopped in the dose increasing stage, Isotonic Regression will be implemented by PAVA (Pool Adjacent Violators Algorithm) to estimate the probability of DLT occurrence in each dose group [4], Then, MTD will be selected as the highest dose

	whose DLT rate estimate is closest to the target DLT rate, but does not exceed the upper limit of the equivalent interval of DLT rate.<br>Within the expected effective dose range, the investigator may adjust the magnitude of dose escalation, increase the intermediate dose and the rate of dose escalation, or perform a dose reduction climb, after communication with the sponsor medical personnel.<br>If during the first cycle of treatment, subjects drop out of the study for reasons other than DLT or voluntarily withdrew from the study, ① if the subject has completed the predicted total dose for DLT and completed at least 21 days of DLT observation, the subject is considered evaluable; ② If the subject has not completed all administration during DLT period, or has not completed at least 21 days of DLT observation, the subject will be considered as unevaluable. After considering the subject's willingness and the investigator's comprehensive assessment of the subject's risks and benefits in the study, a decision may be made to either continue treatment or withdraw from the study. Subjects who develop DLT during cycle 1 and drop out of the study are considered evaluable subjects. Supplementary rules for non-evaluable subjects are as follows:<br>① If the number of evaluable subjects and DLT in the current dose is determined as " Dose increase" or " Reduce to a previous dose" or " Lower to previous dose and cancel all doses above " according to the " Dose increase/decrease Rule ", the subject does not need to be supplemented at the current dose.<br>② If, in accordance with the "Dose Escalation/De-escalation Rules Table", the number of evaluable subjects and the number of DLT occurrences at the current dose are determined to "maintain the current dose", it is necessary to supplement this subject at the current dose or continue enrolling 2-4 additional subjects at this dose level.

	**2. Enrollment expansion (Ib)**<br>The Phase Ib trial is divided into two parts: ① dose extension cohort ② indication extension cohort.<br>① Dose extension cohort: according to the Ia stage for safety, pharmacokinetic and preliminary efficacy data, one or multiple doses (including preliminary RP2D dose levels and other concerned, or similar exposure levels of different frequencies of dosing) are selected for cohort extension to further evaluate the safety and efficacy of BL-M07D1 at different doses.<br>② Indication extension cohort: According to the safety, pharmacokinetics and preliminary efficacy data obtained in Phase Ia, multiple tumor types are selected to further evaluate the safety and efficacy of BL-M07D1 in different indications. The actual enrollment for each indication will be adjusted based on efficacy and safety and is planned to be conducted at 10 to 20 centers.
**Inclusion Criteria:**	1. Signs the informed consent voluntarily and follow the protocol requirements;<br>2. Either Sex;<br>3. Age: ≥18 years and ≤75 years (phase Ia);<br>Age: ≥18 years (phase Ib);<br>4. Has a life expectancy of ≥ 3 months;<br>5. Patients with unresectable locally advanced or metastatic HER2-positive/negative breast cancer and other solid tumors, confirmed by histopathology and/or cytology, who have failed standard treatment, have no access to standard treatment regimens, or are not eligible for standard treatment at the current stage;<br>• HER2-positive: IHC 3+, or IHC 2+ with ISH-positive;

	• HER2-negative: IHC 2+ with ISH-negative, IHC 1+, or IHC 0;<br>6. Subjects must agree to provide archived tumor tissue specimens (within 2 years) or fresh tissue samples from the primary or metastatic lesion. These samples will be used to detect HER2 protein expression in tumor tissue and explore the correlation between this expression and the efficacy indicators of BL-M07D1. If a subject is unable to provide tumor tissue samples, they may still be enrolled after investigator assessment, provided they meet other inclusion/exclusion criteria. However, subjects with HER2 IHC 0 must provide such samples.<br>7. Has at least one measurable lesion based on RECIST V1.1;<br>8. Has an Eastern Cooperative Oncology Group performance status (ECOG PS) 0-1;<br>9. Toxicity of previous antitumor therapy has returned to grade ≤1 as defined by NCI-CTCAE V5.0 (except for asymptomatic laboratory abnormalities at the discretion of investigator, such as elevated ALP, hyperuricemia, and elevated blood glucose; except for toxicity that the investigator determined to have no safety risk, such as alopecia, hyperpigmentation, grade 2 peripheral neurotoxicity etc.);<br>10. Has not serious cardiac dysfunction, left ventricular ejection fraction ≥50%;<br>11. Within 14 days prior to the first administration of the study drug, subjects must not have received blood transfusions, not have used colony-stimulating factors, any cell growth factors, or other injectables, and not have used albumin. On this premise, their organ function must meet the following requirements and standards:<br>a) Marrow Function: Absolute neutrophil count (ANC) $\geq 1.5\times10^9$/L, Platelet count $\geq 90\times10^9$/L, Hemoglobin (Hb) ≥90 g/L;

	b) Hepatic function: Total bilirubin (TBIL≤1.5 ULN), AST and ALT (without liver metastasis) ≤2.5 ULN, AST and ALT (with liver metastasis) ≤5.0 ULN; Albumin≥30 g/L；<br>c) Renal function: Creatinine (Cr)≤1.5 ULN, or creatinine clearance (Ccr) ≥ 50 mL/min (According to the Cockcroft and Gault).<br>12. Coagulation function: international normalized ratio (INR)≤1.5×ULN, and activated partial thromboplastin time (APTT)≤1.5ULN;<br>13. Urinary protein ≤2+ or ≤1000mg/24h;<br>14. For premenopausal women with childbearing potential, a pregnancy test must be taken within 7 days prior to the start of treatment. Serum or urine pregnancy must be negative and must be non-lactating. Adequate barrier contraceptive measures should be taken during the treatment and 6 months after the end of treatment for all participants (regardless of male or female).
**Exclusion Criteria:**	Patients screened for any of the following conditions are excluded from the study:<br>1. Chemotherapy, biological therapy, immunotherapy, radical radiotherapy, major surgery (defined by the investigator), targeted therapy (including small molecule inhibitor of tyrosine kinase), and other anti-tumor therapy within 4 weeks or 5 half-lives (whichever is shorter) prior to the first administration; mitomycin and nitrosoureas treatment within 6 weeks prior to the first administration; oral fluorouracil-like drugs such as S-1, capecitabine, or palliative radiotherapy within 2 weeks prior to the first administration; For traditional Chinese medicines (TCMs) with anti-tumor indications, within 2 weeks prior to the first dose administration.<br>2. Having received prior treatment with ADC drugs conjugated with camptothecin derivatives (topoisomerase I inhibitors) as toxins (Phase Ib only);

	3. Participants with history of severe heart disease, such as: symptomatic congestive heart failure (CHF) ≥ grade 2 (CTCAE 5.0), New York Heart Association (NYHA) ≥ grade 2 heart failure, history of transmural myocardial infarction, unstable angina pectoris etc;<br>4. Participants with prolonged QT interval (male QTc> 450 msec or female QTc> 470 msec), complete left bundle branch block, III grade atrioventricular block;<br>5. Active autoimmune diseases and inflammatory diseases, such as: systemic lupus erythematosus, psoriasis requiring systemic treatment, rheumatoid arthritis, inflammatory bowel disease and Hashimoto's thyroiditis, etc., except for type I diabetes, hypothyroidism that can be controlled only by alternative treatment, and skin diseases that do not require systemic treatment (such as vitiligo, psoriasis);<br>6. Other malignant tumors were diagnosed within 5 years prior to the first administration with the following exceptions: basal cell carcinoma of the skin, squamous cell carcinoma of the skin and/or carcinoma in situ after radical resection;<br>7. Unstable thrombotic events such as deep vein thrombosis, arterial thrombosis, and pulmonary embolism requiring therapeutic intervention within 6 months prior to screening; Thrombus formation associated with infusion set is excluded;<br>8. Patients with massive serous cavity effusion, or symptomatic serous cavity effusion, or poorly controlled serous cavity effusion (defined as requiring 2 or more puncture drainages within 1 month);<br>9. Participants with poorly controlled hypertension by two kinds of antihypertensive drugs (systolic blood pressure>150 mmHg or diastolic blood pressure>100 mmHg);

	10. Patients with pulmonary diseases defined as grade ≥3 per CTCAE v5.0, radiation pneumonitis of grade ≥2, or current or a history of interstitial lung disease (ILD);<br>11. Having active symptoms of central nervous system (CNS) metastasis. However, patients with stable brain parenchyma metastasis may be enrolled, as deemed by the investigator. The definition of "stable" must meet all four of the following criteria:<br>a. No seizure activity for more than 12 weeks, with or without the use of antiepileptic drugs;<br>b. No requirement for glucocorticoid use;<br>c. Imaging stability confirmed by two consecutive MRI scans (with an interval of at least 4 weeks between scans);<br>d. Asymptomatic status for more than 1 month after treatment;<br>12. Participants who have a history of allergies to recombinant humanized antibodies or human-mouse chimeric antibodies or any of the components of BL-M07D1;<br>13. Participants have a history of autologous or allogeneic stem cell transplantation (Allo-HSCT);<br>14. Having received prior anthracycline treatment with a cumulative doxorubicin-equivalent dose exceeding 360 mg/m^2;<br>15. Human immunodeficiency virus antibody (HIVAb) positive, active tuberculosis, active hepatitis B virus infection (HBV-DNA copy number> lower limit of detection) or active hepatitis C virus infection (HCV antibody positive and HCV-RNA > the lower limit of detection);<br>16. Participants with active infections requiring systemic treatment, such as severe pneumonia, bacteremia, sepsis, etc;<br>17. Having participated in another clinical trial within 4 weeks prior to the first administration of the study drug (calculated from the date of the last dose). For clinical trials involving marketed drugs or drugs with a known half-life, refer to exclusion criterion 1;

	18. Women who are pregnant or breastfeeding;<br>19. Other conditions that the investigator believes that it is not suitable for participating in this clinical trial.
**Dosage Regimen:**	**Phase Ia**: BL-M07D1 will be administered intravenously. Cohort A: BL-M07D1 is administered on D1 and D8 every 3 weeks. Cohort B: BL-M07D1 is administered on D1 every 3 weeks. The infusion time of the first administration is 120 min±10min. If the infusion reaction is tolerable during the first administration, the infusion can be completed in 60-120 min±10min for subsequent administration. After the DLT observation period, a 3-day window (+3 days) is allowed for the dosing interval specified in the protocol. The administration will be terminated if disease progression or unacceptable toxicity or for other reasons (e.g. withdrawal of the Informed Consent Form or death). In the phase Ia (dose escalation phase), the administration schedule (including change of administration frequency, impact dose setting, etc.) and dose can be adjusted according to the obtained data of safety, tolerance, PK/PD, etc.<br>**Phase Ib:** According to the safety, pharmacokinetics and preliminary efficacy data of Phase Ia, the appropriate dose group and administration regimen will be selected for phase Ib study.
**Maximum Tolerated Dose:**	In the dose-escalation phase, the highest dose at which estimated DLT rate is closest to the target DLT rate but does not exceed the upper bound of the DLT rate equivalence interval is selected as the MTD. If there are multiple dose groups with DLT rate estimates that all meet the above conditions, then:<br>1) If this estimate DLT rate is less than the target DLT rate, the highest of these doses is selected as the MTD;<br>2) If this estimate DLT rate is greater than the target DLT rate, the lowest of these doses is selected as the MTD.
**Dose-limiting Toxicities:**	The DLT observation period is the first treatment cycle for each dose level. If there is a dose delay, the DLT observation period shall

	be extended accordingly (with the maximum DLT observation period not exceeding 28 days). Toxicity should be graded according to NCI-CTCAE v5.0. During the DLT observation period, prophylactic medications that may affect the assessment of DLT are not permitted prior to the infusion of the study drug, with the exception of those used for nausea and vomiting, stomatitis, skin toxicity, or neutropenia. After the infusion of the study drug, supportive treatment is allowed if toxicity defined as grade ≥2 per CTCAE v5.0 occurs. DLT is defined as follows:<br>Hematological toxicities:<br>• After supportive care, Grade 4 neutropenia lasting >7 days; Grade≥3 febrile neutropenia;<br>• Grade 3 thrombocytopenia lasting >7 days after supportive care, or Grade ≥3 thrombocytopenia with significant bleeding;<br>• Grade 4 anemia lasting >7 days after supportive care.<br>Hepatic organ toxicities:<br>• Grade 4 elevated AST and/or ALT;<br>• AST and/or ALT > 5 × ULN, accompanied by Grade ≥2 elevated blood bilirubin;<br>• For patients without liver metastasis: AST and/or ALT > 5 × ULN for more than 3 consecutive days;<br>• For patients with liver metastasis: ① When baseline AST and/or ALT ≤ 3 × ULN, AST and/or ALT > 5 × ULN for more than 3 consecutive days; ② When baseline AST/ALT > 3 × ULN, AST/ALT > 8 × ULN for more than 3 consecutive days.<br>Other Grade ≥ 3 non-hematological, non-hepatic toxicities, except for:<br>• Grade 3 skin toxicity, mucositis, stomatitis, constipation,

	nausea, vomiting, anorexia, or electrolyte abnormalities, resolved to Grade≤2 (per CTCAE v5.0) within ≤7 days with symptomatic supportive care;<br>• Grade 3 diarrhea: resolved to Grade≤ 2 (per CTCAE v5.0) within 3 days with symptomatic supportive care;<br>• Grade 3 fatigue: resolved to Grade≤1 within 7 days after treatment;<br>• Asymptomatic Grade 3/4 laboratory abnormalities (e.g., elevated ALP, hyperuricemia, hyperglycemia).<br>• Any grade hair loss；<br>• Infusion-related Reactions (IRR).<br>Infusion-related reactions are generally the adverse reactions of blood products, protein products, biological agents that often occur during intravenous infusion and are not considered as DLT. However, if the patient develops a grade 3 or 4 infusion response and is unable to continue treatment with the study drug, an additional patient will be required in the dose group.
**Pharmacokinetic (PK):**	**In dose-escalation phase Cohort A blood collection points are designed as follows:**<br>Cycle 1:<br>PK blood samples will be collected after the 1st infusion of BL-M07D1 at the following time points:<br>- Within 4 hours before the 1st administration<br>- Within 15 minutes after end of infusion (EOI)<br>- 2h±30 min,4h±30min,6h±30min after EOI<br>- Day 2 (24 h±1h after EOI)<br>- Day 3 (48 h±2 h after EOI)<br>- Day 5 (96 h±4 h after EOI)

	- Day 8 (168 h±4 h after EOI)<br>If the patient receives the second dose of the first cycle, the blood collection before the second dose of the first cycle and the blood collection point of the first dose of the cycle are repeated on the 8th day (within 168-4 h after the end of infusion), and only one collection is required. If the patient no longer receives the second dose of the first cycle, the blood collection is only once. PK blood collection should be completed on the 8th day of the first dose of the cycle (within 168-4 h after the end of infusion).<br>PK blood samples will be collected after the 2nd infusion of BL-M07D1 at the following time points:<br>- Day 8 within 4 hours before the 2nd administration<br>- Within 15 minutes after end of infusion (EOI)<br>- 2h±30 min,4h±30min,6h±30min after EOI<br>- Day 9 (24 h±1h after EOI)<br>- Day 10 (48 h±2 h after EOI)<br>- Day 12 (96 h±4 h after EOI)<br>- Day 15 (168 h±4 h after EOI)<br>- Day 22 (336 h -4 h after EOI)<br>Subsequent Cycles:<br>PK blood samples will be collected after the 1st infusion of BL-M07D1 at the following time points:<br>- Within 4 hours before the 1st administration<br>- Within 15 minutes after end of infusion (EOI)<br>PK blood samples will be collected after the 2nd infusion of BL-M07D1 at the following time points:<br>- Within 4 hours before end of the 2nd administration

	- Within 15 minutes after end of infusion (EOI)<br>- Day 22 (336 h -4 h after EOI)<br>If the patient receives the next dose cycle, the blood collection before the next dose cycle D1 is repeated with the blood collection point of the same cycle D22, and only one collection is required. If the patient no longer receives the next dose cycle, the PK blood collection of the same cycle D22 is required.<br>**In dose-escalation phase Cohort B blood collection points are designed as follows:**<br>Cycle 1:<br>PK blood samples will be collected after the 1st infusion of BL-M07D1 at the following time points:<br>- Within 4 hours before the 1st administration<br>- Within 15 minutes after end of infusion (EOI)<br>- 2h±30 min,4h±30 min,6h±30 min after EOI<br>- Day 2 (24 h±1h after EOI)<br>- Day 3 (48 h±2 h after EOI)<br>- Day 5 (96 h±4 h after EOI)<br>- Day 8 (168 h±4 h after EOI)<br>- Day 15 (336 h±4 h after EOI)<br>- Day 22 (504 h -4 h after EOI)<br>Cycle 2:<br>- Within 4 hours before the administration<br>- Within 15 minutes after end of infusion (EOI)<br>- Day 8 (168 h±4 h after EOI)<br>- Day 15 (336 h±4 h after EOI)

	- Day 22 (504 h -4 h after EOI)<br>Subsequent Cycles:<br>- Within 4 hours before the administration<br>- Within 15 minutes after end of infusion (EOI)<br>- Day 22 (504 h -4 h after EOI)<br>If the patient receives the next dose cycle, the blood collection before the next dose cycle D1 is repeated with the blood collection point of the same cycle D22, and only one collection is required. If the patient no longer receives the next dose cycle, the PK blood collection of the same cycle D22 is required.<br>Each PK sampling requires 4 mL of blood to be collected.<br>During the dose escalation phase, based on preliminary pharmacokinetic data obtained from dose groups with PK sampling completed, adjustments to blood collection times for subsequent patients may be made accordingly. For the expanded enrollment phase, blood collection time points will reference the PK study from the dose escalation phase. Only the first three subjects in the 6.2 mg/kg and higher dose groups at the leading study site are required to collect blood samples at all time points for PK studies. Other subjects at the leading site and all subjects at other sites will only require sampling at two time points: within 4 hours before administration and immediately after administration (+15 minutes). Blood collection time points for the expanded enrollment phase may be adjusted based on results from the dose escalation phase.
**Immunogenicity evaluation:**	Detection of ADA and neutralizing antibody (Nab) in serum at the following time points:<br>Before first infusion in cycle 1；<br>From cycle 2 onwards, before the first infusion in each even-numbered cycle (within -4h)；

	Within 0-7 days after end of treatment.<br>Each sampling requires 3ml of blood.
**Study Outcomes:**	**Safety outcomes:**<br>All subjects will be assessed at the end of Cycle 1 (initial 3 weeks) and DLT will be assessed after each dose of Cycle 1 and graded and tabulated according to NCI-CTCAE v5.0. Patient safety and tolerability assessment will be based on the analysis of physical examination, adverse events (including DLT), vital signs, ECOG performance status, echocardiography, electrocardiography, laboratory tests, and concomitant medications, etc.<br>Adverse events and serious adverse events will be graded according to NCI-CTCAE v5.0 criteria. The investigator should assess causal relationship between the adverse event and BL-M07D1.<br>**Immunogenicity outcomes:**<br>Anti-BL-M07D1 antibody and neutralizing antibody.<br>**PK parameters:**<br>AUC_{0-t} and AUC_{0-inf}, T_{max}, C_{max}, $T_{1/2}$, C_{trough}, etc.<br>**Efficacy outcomes:**<br>Tumor response will be evaluated by the investigator according to RECIST version 1.1. Antitumor effects will be assessed by the following parameters:<br>• objective response rate (ORR);<br>• progression free survival (PFS);<br>• overall survival (OS);<br>• disease control rate (DCR);<br>• duration of response (DOR).

**Statistical Analyses:**	Descriptive statistical analysis will be conducted for safety outcomes, efficacy outcomes, PK parameters, immunogenicity outcomes, biomarker measurements, etc., without statistical tests. These data will be presented in graphical summaries or lists.<br>Descriptive statistics on continuous data will include number of cases, means, standard deviations, medians, quartiles, minimum and maximum. Descriptive statistics are used for categorical variables by frequency and percentage.<br>**DLT:**<br>The number and percentage of patients with DLT will be summarized for each dosing schedule and dose level.<br>**Safety variables:**<br>All AEs will be graded according to NCI-CTCAE v5.0 criteria, and the number of cases and incidence will be summarized by system organ classification (SOC) and preferred terminology (PT).<br>**Pharmacokinetic parameters:**<br>The drug-time curve of each patient and the average drug-time curve of each dose group (including semi-logarithmic plots) will be plotted based on the blood concentration-time data measured in the trial, and the non-compartmental model is used to estimate the pharmacokinetic parameters and find the main metabolic kinetic parameters of BL-M07D1.<br>**Efficacy variables:**<br>Statistical description will be made for objective response rate (ORR), disease control rate (DCR), duration of response (DOR), progression-free survival (PFS), and overall survival (OS). 95% CI will be calculated using the Clopper-Pearson interval. Median survival time will be estimated using the Kaplan-Meier method for DOR, PFS and OS.
**Study Duration:**	August 2022 - August 2024

Table1. Phase I Research Schedule

**Assessments**	**Screening**		**Cycle 1 (21 days)[15]**						**Cycle 2**				**Subsequent Cycles**			**EOT[16]**	**Safety Visit[17]**	**Follow -up[18]**
**Day**	**-28~ -1**	**-7 ~ -1**	**1**	**2**	**3**	**8[23]**	**15[24]**	**22[25]**	**1[19][26]**	**8[19][27]**	**15[19][28]**	**22[29]**	**1[19][30]**	**8[19][31]**	**22[29]**	**Within 0-7 days after PD or EOT**	**28 (+7) days after EOT**	
Informed consent	×																	
Screening number	×																	
Eligibility	×																	
Review eligibility		×																
Test tumor samples[1]	×																	
Demographics[2]	×																	
Medical history and prior treatments[3]	×																	
Vital signs[4]	×		×	×	×	×	×	×	×	×	×	×	×	×	×	×	×	
ECOG assessment	×		×	×	×	×	×	×	×	×	×	×	×	×	×	×	×	
Height,weight[20]		×	×			×	×	×	×	×	×	×	×	×	×	×	×	
Physical examinations[5]	×		×	×	×	×	×	×	×	×	×	×	×	×	×	×	×	
Serum virology and HIV testing[6] *	×															×		
ECG[7] *		×	×	×	×	×	×	×	×	×	×	×	×	×	×	×	×	

**Assessments**	**Screening**		**Cycle 1 (21 days)[15]**						**Cycle 2**				**Subsequent Cycles**			**EOT[16]**	**Safety Visit[17]**	**Follow -up[18]**
**Day**	**-28~-1**	**-7 ~ -1**	**1**	**2**	**3**	**8[23]**	**15[24]**	**22[25]**	**1[19][26]**	**8[19][27]**	**15[19][28]**	**22[29]**	**1[19][30]**	**8[19][31]**	**22[29]**	**Within 0-7 days after PD or EOT**	**28 (+7) days after EOT**	
Echocardiography[8] #	×		×(D22±7 at Cycle 1，Cycle 2 and subsequent cycles, D22±7 at even-numbered cycle													×		
Blood routine[9] *		×	×			×	×	×	×	×	×	×	×	×	×	×	×	
Blood biochemistry[10] *		×	×			×	×	×	×	×	×	×	×	×	×	×	×	
Coagulation function[11] *		×				×	×	×	×	×		×	×	×	×	×	×	
Myocardial injury markers[11] #		×				×	×	×	×			×	×		×	×	×	
Urinalysis*		×				×	×	×	×			×	×		×	×	×	
Stool routine+occult blood		×						×	×			×	×		×	×	×	
Pregnancy test[12]		×														×	×	
Tumor assessments (including biomarkers)[13]	×		×(In the first year of treatment, assessments are conducted once every 6 weeks ± 7 days; in the second year of treatment, assessments are performed once every 12 weeks ± 7 days.）													×		×
Whole-body bone nuclide scan (if necessary) [21]																		
Dosing			×			×			×	×			×	×				

**Assessments**	**Screening**		**Cycle 1 (21 days)[15]**						**Cycle 2**				**Subsequent Cycles**			**EOT[16]**	**Safety Visit[17]**	**Follow -up[18]**
**Day**	**-28~-1**	**-7 ~ -1**	**1**	**2**	**3**	**8[23]**	**15[24]**	**22[25]**	**1[19][26]**	**8[19][27]**	**15[19][28]**	**22[29]**	**1[19][30]**	**8[19][31]**	**22[29]**	**Within 0-7 days after PD or EOT**	**28 (+7) days after EOT**	
PK blood collection[14]			×(See Section 8 pharmacokinetic study)															
Immunogenicity blood collection[22]			×(Blood collection time points: before the first administration in Cycle 1; starting from Cycle 2, before the first infusion of the study drug in each even-numbered cycle (within -4 hours); after the end of all treatment cycles (within 0-7 days after end of treatment).													×		
Concomitant medications and therapies	×		×															
AE	×		×															

Note:

1. All patients during the screening period are required to provide tumor tissue specimens obtained within 2 years (either FFPE blocks or approximately 6-12 unstained slides with a specification of 5 μm) to the central laboratory for HER2 protein expression detection, which will be used for exploratory retrospective analysis. If a subject is unable to provide tumor tissue specimens, they may be enrolled after investigator assessment, provided they meet other inclusion/exclusion criteria (patients with HER2 IHC 0 must provide the specimens);

2. Demographic data includes date of birth or age, gender, and ethnicity.

3. Medical history includes past history and current history. Past history records medical history, surgical history, etc. (excluding target tumor history), and previous medications within 4 weeks before signing the ICF; current history records the date of first diagnosis of the target tumor, overall tumor stage at screening, tumor treatment history, etc., including surgical history, chemotherapy history, radiotherapy history, anti-cancer traditional Chinese medicine, and molecular targeted therapy. Smoking and drinking history should also be recorded.

4. Vital signs include pulse, blood pressure, respiration, and body temperature. All subjects require routine electrocardiographic monitoring from 30 minutes before the first dose to 30 minutes after the end of administration. Specific recording points are: 30 minutes ± 10 minutes before administration; the first recording point after the start of administration is 30 minutes ± 10 minutes, and subsequent recording points are every 30 minutes ± 10 minutes; once each at 2 hours ± 30 minutes, 4 hours ± 30 minutes, and 6 hours ± 30 minutes after the end of administration. If vital signs are unstable after medication, electrocardiographic monitoring may be extended until stable. If vital signs are stable during and after the first administration, electrocardiographic monitoring is only performed from 30 minutes before administration to 30 minutes after the end of administration for subsequent study drug infusions, with vital signs recorded every 30 minutes ± 10 minutes. If there are clinical indications, researchers may decide to increase vital sign monitoring.

5. A complete physical examination is performed at baseline and at the end of treatment. ① A complete physical examination includes: the subject's general condition, head and face, skin system, lymph nodes, eyes, ears, nose, throat, mouth, respiratory system, cardiovascular system, abdomen, genitourinary system, musculoskeletal system, nervous system, and mental state. ② For other visits, targeted physical examinations are performed when clinically indicated.

6. Hepatitis B surface antigen (HbsAg), hepatitis B surface antibody (anti-HBs), hepatitis B e antigen (HbeAg), hepatitis B e antibody (anti-Hbe), hepatitis B core antibody (anti-HBc), hepatitis C antibody, and HIV testing. For HbsAg-positive patients, HBV-DNA quantitative PCR testing is required; for HCV antibody-positive patients, HCV-RNA quantitative testing is required.

7. Record all ECG conditions and corresponding values, including but not limited to heart rate, QTc interval, and other clinically significant abnormalities. All subjects undergo 12-lead ECG examinations within 1 hour before the first dose, 30 minutes ± 10 minutes, 2 hours ± 30 minutes, 4 hours ± 30 minutes, and 6 hours ± 30 minutes after the end of administration. For subsequent study drug infusions, 12-lead ECG examinations are only performed within 1 hour before administration and 30 minutes ± 10 minutes after the end of administration. If there are clinical indications, researchers may decide to increase 12-lead ECG examinations.

8. For subjects with a history of pericardial effusion, the study doctor determines whether to additionally increase the frequency of echocardiogram examinations based on clinical indications or medical routines.

9. Blood routine examination items include reticulocytes, hemoglobin, red blood cell count, white blood cell count, neutrophil count, lymphocyte count, eosinophil count, basophil count, monocyte count, and platelet count. The subject's inclusion/exclusion criteria are based on the blood routine results during the screening period to determine whether C1D1 can be administered. The subject's inclusion/exclusion criteria are not guided by the blood routine results before C1D1 administration, and there is no need to wait for the test results before administration. The test results only serve as the baseline value for evaluating subsequent test results during the study. Routine blood tests shall not be repeated within three days. For subsequent visits, blood routine examinations should be completed within 2 days before administration, and administration can only be performed after the results are available.

10. Blood biochemistry examination items include alanine aminotransferase, aspartate aminotransferase, total bilirubin, direct bilirubin, alkaline phosphatase, total protein, albumin, urea, creatinine, fasting blood glucose, total cholesterol, triglycerides, lactate dehydrogenase, and electrolytes (including sodium, potassium, magnesium, chlorine, calcium, and phosphorus). The subject's inclusion/exclusion criteria are based on the blood biochemistry results during the screening period to determine whether C1D1 can be administered. The subject's inclusion/exclusion criteria are not guided by the blood biochemistry results before C1D1 administration, and there is no need to wait for the test results before administration. The test results only serve as the baseline value for evaluating subsequent test results during the study. Blood biochemistry tests shall not be repeated within three days. For subsequent visits, blood biochemistry examinations should be completed within 2 days before administration, and administration can only be performed after the results are available.

When creatinine > 1.5 × ULN, calculate the creatinine clearance rate (using the Cockcroft and Gault formula).

Coagulation function: prothrombin time (PT), activated partial thromboplastin time (APTT), thrombin time (TT), fibrinogen (FIB), and international normalized ratio (INR).

11. Myocardial injury marker tests include myoglobin (Mb), B-type natriuretic peptide (BNP), creatine kinase isoenzyme MB (CK-MB), and cardiac troponin (cTn). Among them, myoglobin (Mb) and creatine kinase isoenzyme MB (CK-MB) tests are determined according to the actual situation of the center and are not mandatory.

12. Serum/urine pregnancy tests are performed during the screening period to rule out pregnancy. If pregnancy is suspected during the trial, serum/urine pregnancy tests should be retested in a timely manner.

13. Enhanced CT or enhanced MRI is preferred for tumor assessment (CT slice thickness does not exceed 5mm). If the patient is allergic to CT contrast agents, plain CT or enhanced MRI can be performed. For special lesions (such as skin lesions), only clinical examinations are acceptable (relevant color photos need to be archived). The imaging examination sites at baseline should include: neck, chest, entire abdomen (including pelvis). If the patient has had imaging examinations within 28 days before the first dose that meet the requirements, no repeated examinations are needed. All subjects should undergo brain imaging examinations at baseline, preferably enhanced cranial MRI. If the patient subsequently develops symptoms related to brain metastasis, brain imaging re-examinations can be performed; if the patient has no relevant symptoms, no re-examinations are performed. The same imaging technology should be used for subjects throughout the study. If progression is suspected during the study, additional imaging examinations can be performed at any time. Imaging examinations are counted from the start of administration. During treatment: in the first year of treatment, evaluations are performed every 6 weeks ± 7 days; in the second year of treatment, evaluations are performed every 12 weeks ± 7 days; in the first year after the last dose, evaluations are performed every 12 weeks ± 7 days; in the second year after the last dose, evaluations are performed every 24 weeks ± 7 days.

For tumor marker tests, test results within 14 days before signing the informed consent form do not need to be repeated during the screening period.

The sponsor will collect and properly manage patients' imaging data with sensitive information removed (including but not limited to patients' names, addresses, etc.), which will be retained for subsequent exploratory analysis or retrospective analysis.

14. See Section 8. Pharmacokinetic Study for pharmacokinetic blood collection points.

15. Cohort A: Administered on Day 1 and Day 8, with a 3-week cycle. The first cycle is the DLT observation period, during which dose adjustments are not allowed. If there is a dose delay, the DLT observation period shall be extended accordingly (with the maximum DLT observation period not exceeding 28 days); Cohort B: Administered on Day 1, with a 3-week cycle; the first cycle is the DLT observation period (3 weeks in total), during which dose adjustments are not allowed.

16. If a subject terminates treatment for reasons other than disease progression, imaging examinations must still be performed to evaluate efficacy. Test results within 7 days before treatment termination do not need to be repeated.

17. Follow up on drug-related adverse events present at the end of treatment visit and collect new adverse events; serious adverse events are followed up until stable or returned to baseline.

18. For patients who terminate treatment due to reasons other than disease progression, after the safety visit, tumor assessments will be performed every 12 weeks ± 7 days in the first year after the last dose; every 24 weeks ± 7 days in the second year after the last dose, until the patient starts new anti-tumor drug treatment, or the patient experiences disease progression, or death, loss to follow-up, withdrawal of informed consent, or termination of the study by the sponsor, whichever occurs first. At the same time, survival follow-up is performed, with a follow-up frequency of once every 8 weeks ± 7 days, or investigators can arrange follow-up according to the actual situation of the subject. Follow-up will continue until 12 months after the last subject completes study participation, or until the patient dies or is lost to follow-up, whichever occurs first. For patients who terminate treatment due to disease progression, after the safety visit, survival follow-up is started, with a follow-up frequency of once every 8 weeks ± 7 days, or investigators can arrange follow-up according to the actual situation of the subject. Follow-up will continue until 12 months after the last subject completes study participation, or until the patient dies or is lost to follow-up, whichever occurs first.

19. For phase Ia dose-escalation patients, after the DLT observation period, the subsequent administration cycle interval allows a 3-day window (+3 days). If delayed administration occurs, it is calculated based on the actual administration time; for phase Ib expansion patients, the administration interval allows a 3-day window (+3 days). If delayed administration occurs, it is calculated based on the actual administration time. For delayed administration rules, see "Section 3.4.4. Administration Plan and Definitions of DLT and MTD, part 2) Suspension, Termination of Administration or Dose Adjustment".

20. Height and weight will only be measured during the screening period. During subsequent treatment cycles, only weight needs to be measured before drug

administration (weight measurement at the Day 8 visit of each cycle is only required for Cohort A; Cohort B does not need to undergo weight measurement at this visit). The weight measurement window is -1 day.

21. During the study, investigators determine whether a whole-body bone scan is needed based on the subject's condition and clinical decision-making needs.

22. Immunogenicity blood collection points are: before the first dose of the first cycle; starting from the second cycle, before the first infusion of the test drug in each even-number cycle (within -4 hours); after the end of all treatment cycles (within 0-7 days after treatment termination).

23. Visit checkpoints: examination on day 8 ± 2 days of the first cycle (21 days).

24. Visit checkpoints: examination on day 15 ± 2 days of the first cycle (21 days).

25. Visit checkpoints: examination on day 22 ± 2 days of the first cycle (21 days). If the patient receives the next cycle of administration, the blood collection on D1 of the next cycle overlaps with the D22 blood collection point of this cycle, and only one collection is needed. If the patient no longer receives the next cycle of treatment, the PK blood collection on D22 of this cycle needs to be completed. For patients who no longer receive the second cycle of treatment, complete the visit study content after the end of the first cycle.

26. Visit checkpoints: examination on day 1 ± 2 days of the second cycle.

27. Visit checkpoints: examination on day 8 ± 2 days of the second cycle.

28. Visit checkpoints: examination on day 15 ± 2 days of the second cycle.

29. Visit checkpoints: examination on day 22 ± 2 days of the second and subsequent cycles. If the patient receives the next cycle of administration, the D1 visit point of the next cycle overlaps with the D22 visit point of this cycle, and only one visit is performed.

30. Visit checkpoints: examination on day 1 ± 2 days of subsequent cycles.

31. Visit checkpoints: examination on day 8 ± 2 days of subsequent cycles (**this visit is not performed for Cohort B**).

32. Cohort A: administered on D1 and D8 of each cycle; Cohort B: administered on D1 of each cycle.

33. Subject screening number assignment: After a subject signs the informed consent form, a 3-digit screening number (e.g., 001, 002) will be assigned in sequential order; subsequently, if screening is successful, an enrollment number will be assigned to the subject, which is composed of a 2-digit site number plus the 3-digit screening number (e.g., 01001, 01002). If screening fails, the subject’s enrollment number will not be supplemented—for example, if Subject 003 fails screening and Subject 004 succeeds, the enrollment number 01003 (for Subject 003) becomes invalid and will not be supplemented, and the enrollment number for Subject 004 will

be 01004.

Rules for name abbreviations: Name abbreviations shall be uniformly 4 characters long (e.g., Li Si → LISI; Li Sisi → LSSI). For special cases where the abbreviation is less than 4 characters, use "-" to make up the length (e.g., Li E → LIE-).

* During the screening period, one retest is allowed;

# During the screening period, retests are not allowed.

<u>For adverse reactions occurring during the study, investigators can adjust the testing items and frequency according to the patient's situation.</u>

LIST OF ABBREVIATIONS

ADA	Anti-Drug Antibody
AE	Adverse Event
ALP	Alkaline Phosphatase
ALT	Alanine aminotransferase
ANC	Absolute Neutrophil Count
APTT	Activated Partial Thromboplastin Time
AST	Aspartate aminotransferase
AUC	Area Under the Curve
$AUC_{0\text{-}inf}$	Area Under the Curve (0-inf)
$AUC_{0\text{-}t}$	Area Under the Curve (0-t)
Ccr	Creatine Clearance Rate
CHF	Congestive Heart Failure
CL	Clearance
C_{max}	Maximum Drug Concentration
Cr	Creatine
CR	Complete Response
CRA	Clinical Research Associate
CRO	Contract Research Organization
CSR	Clinical Study Report
CTCAE	Common Terminology Criteria for Adverse Events
CV	Coefficient of Variation
DLT	Dose Limiting Toxicity
DOR	Duration of Response
ECG	Electrocardiogram
ECOG	Eastern Cooperative Oncology Group
eCRF	Electronic Case Report Form
EDC	Electronic Data Capture
FIB	Fibrinogen
FDA	Food and Drug Administration
G-CSF	granulocyte colony-stimulating factor
HED	Human Equivalent Dose
FIH	First-in-human
FISH	Fluorescence in situ Hybridization
GCP	Good Clinical Practice
GLP	Good Laboratory Practices
HER2	Human Epidermal Growth Factor Receptor 2
HIV	Human Immunodeficiency Virus
HNSTD	Highest Non-Severely Toxic Dose
ICH	International Concil on Harmonization
IEC	Independent Ethics Committee
IHC	Immunohistochemistry
ILD	Interstitial Lung Disease

INR	International Normalized Ratio
IRR	Infusion-related Reactions
LVEF	left Ventricular Ejection Fraction
MedDRA	Medical Dictionary for Regulatory Activities
MTD	Maximum tolerated dose
NCI	National Cancer Institute
ND	Not Detectable
NOAEL	No Observed Adverse Effects Level
NYHA	New York Heart Association
ORR	Objective Response Rate
OS	Overall Survival
PD	Progressive Disease
PFS	Progression Free Survival
PK	Pharmacokinetics
PR	Partial Response
PT	Prothrombin Time
Q3W	Every 3 week
Q2W	Every 2 week
QW	Every 1 week
QTc	Revised QT Period
RBC	Red Blood Cell
RECIST	Response Evaluation Criteria in Solid Tumors
RP2D	Recommended Phase II Dose
SAE	Serious Adverse Event
SAP	Statistics Analysis Plan
SAR	Statistics Analysis Report
SOC	System Organ Class
SD	Stable Disease
$t_{1/2}$	Half–life
TBIL	Total Bilirubin
TT	Thrombin Clotting Time
T_{max}	Time at Which the Maximum Drug Concentration Occurs
ULN	Upper Limit of Normal
V_d	Apparent Volume of Distribution
WBC	White Blood Cell
WHODD	WHO Drug Dictionary

1. Background

Currently, antibody-drug conjugates (ADCs) have become a rapidly expanding category in tumor-targeted therapeutics, with new targets, structures, and varieties continuously entering clinical development. Gemtuzumab Ozogamicin (Mylotarg), the first marketed ADC, was approved in 2000 through the FDA's accelerated approval process for patients with acute myeloid leukemia (AML). However, it was subsequently withdrawn due to potential severe hepatic and pulmonary complications. After dose adjustments and the supplementation of additional data, Gemtuzumab Ozogamicin was finally reintroduced in 2017 for the treatment of CD33-positive acute myeloid leukemia (AML) patients [3]. Beyond Gemtuzumab Ozogamicin, growing evidence indicates three key factors limiting the clinical efficacy and safety of ADCs: ① Selection of target antigens: Target antigens must be highly expressed in tumors but not expressed or weakly expressed in healthy cells [4], present on the surface of tumor cells [5], and capable of internalization [6]; ② Linker design: Linkers must ensure the stability of ADCs during blood circulation to prevent toxin release in off-target tissues [7]. Additionally, linkers should enable toxin release and exert cytotoxic effects at the tumor site and/or within tumor cells [8]; ③ Toxin design: Cytotoxic small molecules, once released at the tumor site and/or within tumor cells, must possess sufficient activity to kill tumors [9-10].

HER2 plays a crucial role in tumor cell proliferation and metastasis. HER2 protein is highly expressed in various tumors, including solid malignancies such as breast cancer (BC), gastric or gastroesophageal junction cancer (GC/GEJ), non-small cell lung cancer (NSCLC), ovarian cancer (OC), endometrial cancer (EC), and urothelial cancer (UC).

Currently in China, only Roche’s and Remegen’s HER2-targeted ADCs have been approved, for HER2-positive early breast cancer and HER2-overexpressing locally advanced or metastatic gastric cancer, respectively. Globally, a total of three HER2-targeted ADCs have received marketing approval: Roche’s T-DM1 and Daiichi

Sankyo's DS-8201 were first approved by the US FDA, while Remegen's RC48 was first approved by China's NMPA. Meanwhile, numerous domestic and international pharmaceutical companies are developing HER2-targeted ADCs.

BL-M07D1 is an anti-HER2 ADC independently developed by the sponsor. It can deliver small-molecule toxins precisely to HER2-positive tumor sites and/or within tumor cells, enabling targeted killing of tumor cells while reducing damage to normal cells. It holds the potential for breakthrough clinical efficacy.

Introduction to the mechanism of BL-M07D1:

BL-M07D1 is a recombinant antibody-drug conjugate with anti-tumor activity, and its tumor-killing mechanisms are as follows: A. BL-M07D1 can specifically bind to HER2 on the surface of tumor cells, enter tumor cells through endocytosis, and release the toxin Ed-04 (a camptothecin derivative and topoisomerase I inhibitor) via enzymatic cleavage in lysosomes, which blocks DNA replication in tumor cells, disrupts DNA structure, and thereby kills tumor cells; B. The antibody component of BL-M07D1 is the anti-HER2 monoclonal antibody trastuzumab, which can specifically bind to HER2 on the surface of tumor cells, block the cleavage of the extracellular domain of HER2, and inhibit the activation of downstream signaling pathways, thereby enhancing anti-tumor activity; C. The Fc segment of the antibody component in BL-M07D1 can mediate ADCC (antibody-dependent cellular cytotoxicity) effects, further enhancing the killing of tumor cells.

1.1. Introduction of Investigational Drug

1.1.1. Drug name

General name: BL-M07D1 for injection

Trade name: to be applied

Pingyin: zhusheyong BL-M07D1

1.1.2. Chemical Structure and Physicochemical Properties

Chemical structure:

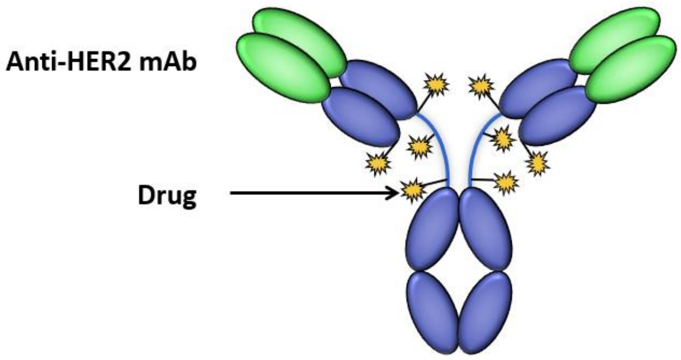

Active ingredient: antibody-drug conjugate BL-M07D1

Appearance: [redacted]

Specification: 50 mg/vial or 50 mg

Stability and preserve conditions: [redacted]

1.2. Summary of nonclinical research

1.2.1. Safety Research Results of Pre-clinical study and risk warnings

1.2.1.1 Safety Research Results of Pre-clinical study

[redacted]

[redacted]

[redacted]

[redacted]

[redacted]

[redacted]

[redacted]

1.2.1.2 Implications of preclinical safety results for clinical risk warnings

[redacted]

[redacted]

[redacted]

[redacted]

[redacted]

[redacted]

1.2.2. Properties of pre-clinical pharmacodynamics

[redacted]

[redacted]

[redacted]

[redacted]

[redacted]

[redacted]

[redacted]

[redacted]

[redacted]

[redacted]

[redacted]

[redacted]

1.2.3. Properties of pre-clinical pharmacokinetics

[redacted]

[redacted]

[redacted]

2. Overall Study Plan

2.1. Study Plan of clinical trial

The clinical development of BL-M07D1 will be planned in accordance with the relevant guidelines of NMPA (National Medical Products Administration), including Guidelines for General Considerations in Drug Clinical Trials and Technical Guidelines for Clinical Trials of Antitumor Drugs.

A series of clinical trials are planned to be designed in phases I, II, and III to investigate the safety and efficacy of BL-M07D1 in HER2-positive/negative breast

cancer and other solid tumors, and ultimately determine the optimal indication population, optimal combination therapy strategy, and dosing regimen for BL-M07D1.

2.1.1. Phase I Clinical trial

This clinical study is the first in human clinical study of BL-M07D1, which is defined as a phase I study of the safety, tolerability, pharmacokinetics and preliminary efficacy of BL-M07D1 in patients with locally advanced or metastatic HER2-positive/low-expressing breast cancer and other solid tumors. The study will be carried out in patients with locally advanced or metastatic HER2-positive/negative breast cancer and other solid tumors who have failed or could not access a standard therapy. The study is divided into two phases: a dose escalation phase (Phase Ia) and a cohort expansion phase (Phase Ib).

The phase Ia will adopt a design combining accelerated titration and the "3+3" method, and is planned to enroll approximately 28 patients with locally advanced or metastatic HER2-positive/low-expression breast cancer and other solid tumors (to be determined based on study progress). The study is scheduled to be conducted at 1 to 10 research centers

Phase Ib is divided into two parts, a dose expansion cohort and an indication expansion cohort. The dose expansion cohort will select 1 or more doses for cohort expansion to further evaluate the safety and efficacy of BL-M07D1 at different doses. The indication expansion cohort will select multiple tumor types to further evaluate the safety and efficacy of BL-M07D1 in different indications. The actual number of participants for each indication will be adjusted according to the efficacy and safety, and it is planned to be carried out in 10 to 20 research centers. The main tasks of Phase Ia and Phase Ib clinical study are shown in Table 2.1.1-1.

Table 2.1.1-1 The main tasks of Phase Ia and Phase Ib clinical research

Phase	Research content	Trial population and objectives
**Phase Ia**	Single-drug	● Trial population: locally advanced or metastatic HER2-

	tolerance and preliminary pharmacokinetic/pharmacodynamic studies	positive/low-expression breast cancer and other solid tumors.<br>● Trial objective: To observe the safety and tolerability of BL-M07D1 in patients with locally advanced or metastatic HER2-positive/low-expression breast cancer and other solid tumors, so as to determine the maximum tolerated dose (MTD) and dose-limiting toxicity (DLT).
**Phase Ib**	Dose- and tumor-specific cohort expansion studies	● Trial population: the specific tumor type will be determined according to the results of phase Ia.<br>● The actual tumor type and number of cases may be adjusted according to the results of Phase Ia. If the efficacy of an indication cohort is confirmed to be significant, a further increase in the number of enrolled patients is allowed. Trial objective: Select multiple doses (including preliminary RP2D and other effective dose levels, or fixed dose administration with similar exposure) based on the results of Phase Ia research to further observe the safety and preliminary efficacy of BL-M07D1 in specific tumor types, and determine the recommended phase II dose (RP2D).

2.1.2. Phase II Clinical trial

At this phase, based on the results of Phase I clinical pharmacology study and preliminary efficacy, the exploration of drug combination in Phase II will be carried out, and the dose and regimen of the combined drug will be determined. In addition, single drug will be keeping on in phase II clinical studies, and will be explored the efficacy and safety on designated tumor types, as shown in Table 2.1.2-1.

Table 2.1.2-1 The main tasks of Phase II clinical research

**Phase**	**Research content**	**Trial population and objectives**
Phase II	Multi-cohort study of BL-M07D1 combined with	● Trial population and dosage regimen: The actual tumor types and combination regimens are formulated based on Phase I clinical data, which may include:

	targeted therapy or immunotherapy	Cohort A BL-M07D1+ targeted therapy<br>Cohort B BL-M07D1+ Immunotherapy<br>Combination studies actually conducted may be adjusted based on Phase I trial results or data from nonclinical studies.<br>● Study objective: To evaluate the safety, tolerability and pharmacokinetic characteristics of BL-M07D1 combined with targeted therapy or immunotherapy, and to determine the dose for combination therapy and administration schedule.
Phase II	Multiple Phase II clinical studies of BL-M07D1 as a single drug in multiple tumor types	● Study population: The specific tumor types depends on the results of the Phase I clinical trial.<br>– The patient may have a tumor type that has been included in the phase I clinical trial or a tumor type that has not been included;<br>– The patient may have no standard treatment, or no standard treatment after previously treated second/third line treatment, or patients who are intolerant to standard treatment;<br>– Actual Phase II monotherapy studies may be adjusted based on the results of Phase I trial or the results of preclinical study.<br>Study objective: To evaluate the efficacy of BL-M07D1 monotherapy in study population.

2.1.3. Phase III Clinical trial

The task at this phase is to select the most clinically valuable tumor types on the basis of Phase I or/and Phase II clinical trials, and to expand the sample size for confirmatory trials of efficacy, including but not limited to BL-M07D1 monotherapy or combination, at the same time, to further verify the safety and efficacy of BL-M07D1 in designated tumor types. Enrolled patients may be newly diagnosed patients or patients who have disease progressed after previous treatment. After confirming the efficacy of BL-M07D1 in the tumor types selected in the preliminary studies, other

potentially responsive tumor types will be chosen for expanded indication research.

This phase aims to obtain the efficacy and safety of BL-M07D1, evaluate the benefit/risk relationship, and provide sufficient evidence for being launched.

Table 2.1.3-1 The main tasks of Phase III clinical research

Phase	Research content	Trial population and objectives
Phase III	Confirmatory trial of BL-M07D1 monotherapy or combination therapy	● Trial population: According to the results of phase I or/and phase II clinical trials, phase III clinical confirmatory trials will be conducted on tumor types with confirmed efficacy. Enrolled patients may be newly diagnosed patients or patients who have disease progressed after previous treatment. The actual tumor types for Phase III studies may be adjusted based on the results of Phase I/II trials and preclinical studies.<br>● Trial objective: To select the most clinically valuable tumor types, expand the sample size for confirmatory trials of efficacy, including but not limited to BL-M07D1 monotherapy or combination, at the same time, to further verify the safety and efficacy of BL-M07D1 in designated tumor types.<br>To obtain the results of efficacy and safety of BL-M07D1, evaluate the benefit/risk relationship, and provide sufficient evidence for being launched.

2.2. Evaluation methods of Drug safety

Drug safety will be evaluated by monitoring all AEs in this study, and be defined and graded according to NCI-CTCAE v5.0. Patients were assessed for safety (including laboratory metrics) on a planned schedule. All enrolled patients will be evaluated for safety by clinical symptoms, signs and laboratory assays prior to and periodically during their participation in the study. Safety evaluation includes medical interviews, AE records, physical examination, laboratory assays (blood routine, blood biochemistry, etc.) and other evaluation methods, and descriptive statistical analysis

was performed on all safety data in the safety analysis set.

It will mainly be analyzed from the following aspects:

1） Dose-Limiting Toxicity (DLT)

DLT is the primary endpoint of the Phase Ia clinical study.

The first cycle is the DLT observation period, during which dose adjustments are not allowed. If there is a delay in drug administration, the DLT observation period will be extended accordingly (with the maximum DLT observation period not exceeding 28 days). DLT will be evaluated to determine whether to escalate to the next dose level; if the escalation criteria are met, the study will proceed to dose escalation for the next dose level.

2） Analysis of Drug exposure

Analysis of drug exposure will be performed in Phase I, II, and III clinical trials.

PK samples will be taken at specified time points in the research flow chart, analyze and describe the patient's plasma concentration-time characteristics, and calculate the pharmacokinetic parameters such as the patient's total exposure (area under the curve AUC_{0-t} and/or AUC_{0-inf}). Pharmacodynamic analysis will be performed based on the pharmacodynamic (PD) analysis dataset. Pharmacodynamic parameters will be summarized by each visit time, including percent change or change from baseline, which including the relationship between exposure levels of BL-M07D1 and cytokine release.

3） Adverse event (AE) analysis

Adverse events will be analyzed in Phase I, II, and III clinical trials.

All adverse events that occur after a patient treated with the investigational product must be fully documented on the patient's electronic case report form (eCRF). Abnormal laboratory test results (eg, blood biochemistry, blood routine, coagulation function, urine routine) or other abnormal test results (eg, ECG, CT/MRI, or vital signs), which are judged to be clinically significant by the investigator, will be recorded as AE or SAE, including abnormal laboratory test results or other abnormal test results with

clinical meaning that worsened significantly at baseline and during the study period. All adverse events will be coded and summarized using MedDRA. The toxicity of adverse events will be classified, summarized and tabulated according to NCI CTCAE v5.0. Numbers of cases and incidences will be summarized by system organ class (SOC) and preferred terms.

4） Laboratory tests

Laboratory tests will be performed in Phase I, II, and III clinical trials.

Evaluate various indicators of laboratory tests (such as blood routine, serum biochemistry, etc.) by the reference values of each laboratory, and label abnormal values. Descriptive statistical analyses of laboratory tests and their changes from baseline will be performed. Laboratory tests will be graded according to NCI-CTCAE v5.0 and summarized by the grade of NCI-CTCAE v5.0.

5） Vital signs and physical examination

Vital signs will be evaluated in Phase I, II, and III clinical trials

Any abnormalities identified during the baseline period should be recorded on the eCRF's General History and Baseline Status page. Descriptive statistical analyses will be performed to vital signs (blood pressure, pulse, respiratory rate, body temperature), physical examination, and their changes from baseline at all visit times, and the results will be presented by patient and visit time.

3. Study Contents

3.1. Study Objectives

3.1.1. Dose Escalation (Ia)

1)Primary Objective

- Observing the safety and tolerability of BL-M07D1 in patients with locally advanced or metastatic HER2-positive/low-expression breast cancer and other solid

tumors to determine the maximum tolerance dose (MTD) and dose-limiting toxicity (DLT) of BL-M07D1.

2)Secondary Objectives

- To assess the pharmacokinetic (PK) profile of BL-M07D1 in patients with locally advanced or metastatic HER2-positive/low-expression breast cancer and other solid tumors.
- To assess the incidence of immunogenicity of BL-M07D1 in patients with HER2-positive/low-expression breast cancer and other solid tumors.

3)Exploratory Objective:

- To detect the expression of HER2 protein in tumor tissues and conduct an exploratory study on the correlation between HER2 protein expression and the efficacy outcomes of BL-M07D1.

3.1.2. Enrollment Expansion (Ib)

1)Primary Objective

- Further observing the safety and tolerability of BL-M07D1 at the recommended phase Ia dose to determine the recommended phase II dose (RP2D).

2)Secondary Objectives

- To evaluate the preliminary efficacy of BL-M07D1 in patients with locally advanced or metastatic HER2-positive/negative breast cancer and other solid tumors.
- To further assess the PK of BL-M07D1.
- To assess the incidence of immunogenicity of BL-M07D1.

3)Exploratory Objective:

- According to the results of phase Ia, biomarkers will be optimized to further study the correlation between selected biomarkers and efficacy outcomes.

3.2. Study Endpoints

3.2.1. Dose Escalation (Ia)

1)Primary Endpoint

- Dose-limiting toxicity (DLT), Maximum tolerance dose (MTD).

2)Secondary Endpoints

- Type, frequency and severity of treatment-emergent adverse event (TEAE) during the treatment of BL-M07D1； Abnormal physical examination; Abnormal laboratory tests;
- Pharmacokinetic (PK) parameters: C_{max}, T_{max}, $T_{1/2}$, AUC_{0-t}, CL, C_{trough} etc;
- Immunogenicity: incidence of anti-BL-M07D1 antibody；

3)Exploratory Endpoints

- The expression level of HER2 in tumor tissues, as a biomarker for predicting the efficacy outcomes of BL-M07D1;
- Neutralizing activity of anti-BL-M07D1 antibody.

3.2.2. Enrollment Expansion (Ib)

1)Primary Endpoint

- Recommended phase II dose (RP2D).

2)Secondary Endpoints

- Type, frequency and severity of treatment-emergent adverse event (TEAE) during the treatment of BL-M07D1; Abnormal physical examination; Abnormal laboratory tests;
- Objective response rate (ORR), disease control rate (DCR), duration of response (DOR);
- Pharmacokinetic (PK) parameters: C_{max}, T_{max}, $T_{1/2}$, AUC_{0-t}, CL, C_{trough},etc;
- Immunogenicity: incidence of anti-BL-M07D1 antibody.

3)Exploratory Endpoints

- Progression-free survival (PFS), overall survival (OS);
- The expression level of HER2 in tumor tissues, as a biomarker for predicting the efficacy outcomes of BL-M07D1;
- Neutralizing activity of anti-BL-M07D1 antibody.

3.3. Study Stage

Phase I, including dose escalation (Ia) and enrollment expansion (Ib).

3.4. Study Design

3.4.1. Overall Design of Study

This is an open, multicenter, dose escalation, and expanded enrollment nonrandomized Phase I clinical study evaluating the safety, tolerability, pharmacokinetic characteristics, and initial efficacy of injectable BL-M07D1 in locally advanced or metastatic HER2-positive/negative breast cancer and other solid tumors. the research is divided into two stages: stage of dose escalation (Ia) and enrollment expansion (Ib).

Accelerated titration and a combined i3+3 design was used for dose escalation to evaluate the safety, tolerability, pharmacokinetic characteristics and preliminary efficacy of BL-M07D1 in patients with locally advanced or metastatic HER2-positive/low-expression breast cancer and other solid tumors in Phase Ia. Cohort A: The study drug is administered via intravenous infusion on Day 1 and Day 8, with a 3-week cycle. Cohort B: The study drug will be administered via intravenous infusion on Day 1, with a 3-week cycle. The first cycle of each dose group is the dose-limiting toxicity (DLT) observation period, during which DLT evaluation will be performed. During the DLT observation period, dose adjustments are not allowed. If there is a delay in drug administration, the DLT observation period will be extended accordingly (with the

maximum duration of the DLT observation period not exceeding 28 days).

Phase Ib will select one or more safe and effective dose groups for an expanded study based on the safety, pharmacokinetic and preliminary efficacy data obtained in Phase Ia to further evaluate the safety, preliminary efficacy and pharmacokinetic characteristics of BL-M07D1. Cohort A: The study drug is administered via intravenous infusion on Day 1 and Day 8, with a 3-week cycle. Cohort B: The study drug will be administered via intravenous infusion on Day 1, with a 3-week cycle.

Setting of Test Dose Group

The original dose-escalation plan:

The initial preset dose groups of BL-M07D1 were 1.0 mg/kg, 3.0 mg/kg, 4.0 mg/kg, 5.0 mg/kg, and 6.0 mg/kg. Starting from 3.0 mg/kg, the default increment for dose escalation is 1.0 mg/kg. The dose escalation increment will be adjusted as follows (see the Dose Escalation Adjustment Table for details):

① Beginning from 3.0 mg/kg, if a dose-limiting toxicity (DLT) occurs for the first time, the adjusted increment for subsequent dose escalations will be modified to 0.5 mg/kg. If the first DLT occurs at 6.0 mg/kg, no adjustment will be made.

② If multiple DLTs occur at the same dose, or if DLTs occur at subsequent different doses, the default dose escalation increment will no longer be adjusted. However, the increment may be adjusted or intermediate doses may be added after consultation with the investigator(s).

Dose adjustment table during dose escalation:

The dose group in which the first DLT occurred	Dose groups
3.0 mg/kg	1.0, 3.0, 3.5, 4.0, 4.5, 5.0, 5.5, 6.0 mg/kg
4.0 mg/kg	1.0, 3.0, 4.0, 4.5, 5.0, 5.5, 6.0 mg/kg
5.0 mg/kg	1.0, 3.0, 4.0, 5.0, 5.5, 6.0 mg/kg
6.0 mg/kg	1.0, 3.0, 4.0, 5.0, 6.0 mg/kg

In this study, administration is scheduled on Day 1 (D1) and Day 8 (D8) of each 3-week cycle, with the first cycle serving as the DLT observation period. During dose escalation under this administration schedule, for dose groups that have completed the DLT observation and demonstrated favorable safety profile, further exploration may proceed at twice the dose or a dose mutually determined by consultation with investigators, following a once-every-3-week (Q3W) schedule in 3-week cycles. For example, after completing the exploration of 3.0 mg/kg administered on D1 and D8 of each 3-week cycle (with a total Q3W dose of 6.0 mg/kg), exploration of 6.0 mg/kg administered on D1 of each Q3W cycle may be initiated. The number of subjects enrolled for exploring administration frequency will not be counted in the sample size for dose escalation.

If dose reduction is required in accordance with escalation rules or due to other safety considerations, adjustments will be made as follows, or alternative doses may be selected upon discussion and agreement between the sponsor and investigators:

① If the interval between the intolerable dose (DLT uncleared) and the highest tolerated dose that has completed escalation (DLT cleared) is 2.0 mg/kg, the dose will be reduced to [highest tolerated dose that completed escalation + 1.5 mg/kg]. For example, if 1.0 mg/kg is tolerated but 3.0 mg/kg is intolerable, the dose will be reduced to 2.5 mg/kg.

② If the interval between the intolerable dose (DLT uncleared) and the highest tolerated dose that completed escalation (DLT cleared) is 1.5 mg/kg, the dose will be reduced to [highest tolerated dose that completed escalation + 1.0 mg/kg]. For example, if 1.0 mg/kg is tolerated but 2.5 mg/kg is intolerable, the dose will be reduced to 2.0 mg/kg.

③ If the interval between the intolerable dose (DLT uncleared) and the highest tolerated dose that completed escalation (DLT cleared) is 1.0 mg/kg, the dose will be reduced to [highest dose that completed escalation + 0.5 mg/kg]. For example, if 3.0 mg/kg is tolerated but 4.0 mg/kg is intolerable, the dose will be reduced to 3.5 mg/kg.

The current dose-escalation plan:

Considering the comprehensive balance between subject safety and benefits, after completing the dose escalation of the 1.0 mg/kg dose group (i.e., Cohort A) — administered on Day 1 (D1) and Day 8 (D8) with a once-every-3-week (Q3W) schedule — the subsequent escalation doses will be reduced, and the escalation protocol will be adjusted to **Cohort B**. Cohort B will use the following doses: 2.6 mg/kg, 3.2 mg/kg, 3.8 mg/kg, 4.4 mg/kg, 5.0 mg/kg, 5.6 mg/kg, 6.2 mg/kg, 6.8 mg/kg, and 7.4 mg/kg, administered on D1 of each 3-week cycle.

If dose adjustment is required in accordance with escalation rules or due to other safety considerations, the dose may be reduced to [highest dose that has completed escalation + 0.3 mg/kg]. For example, if 3.8 mg/kg is tolerated but 4.4 mg/kg is not, the dose will be reduced to 4.1 mg/kg. Alternatively, after discussion between investigators and the sponsor, other appropriate doses or administration schedules may be selected for the ongoing dose escalation study.

Determination of initial dose:

[redacted]

[redacted]

[redacted]			[redacted]	[redacted]
[redacted]	[redacted]	[redacted]	[redacted]	[redacted]
	[redacted]	[redacted]	[redacted]	[redacted]
	[redacted]	[redacted]	[redacted]	[redacted]

			[redacted]	
	[redacted]	[redacted]	[redacted]	[redacted]
[redacted]	[redacted]	[redacted]	[redacted]	[redacted]

[redacted]

[redacted]

[redacted]

[redacted]

[redacted]

Dose Escalation Cohort Design

Based on the starting dose setting of this study drug, the effective dose of the marketed competitor drug DS-8201, and the tolerability of patients in the previous dose escalation phase, the dose escalation cohorts of this study drug are designed with full consideration of the patient's benefit-risk ratio, as follows:

Cohort A: D1, D8, Q3W<br>Dose (mg/kg)	Cohort B: D1, Q3W<br>Dose (mg/kg)
1.0	-
-	2.6
-	3.2
-	3.8
-	4.4
-	5.0
-	5.6
-	6.2
-	6.8
-	7.4

[redacted]

Considering that this product has the activity and toxicity of small molecules, after completing the dose escalation of the starting dose (1.0 mg/kg, administered on Days 1 and 8, with a 3-week cycle), dose escalation for Cohort B will be conducted: administration once every 3 weeks (Q3W) with a 3-week cycle, as shown in the table above. This is to allow patients sufficient time to recover from small molecule-induced myelosuppression and improve treatment compliance.

In summary, to explore the maximum tolerated dose (MTD) and therapeutic window of this product, after the completion of the starting dose, 9 dose levels will be designed for Cohort B: 2.6, 3.2, 3.8, 4.4, 5.0, 5.6, 6.2, 6.8, and 7.4 mg/kg, administered on Day 1 with a 3-week cycle. The accelerated titration strategy will be adopted for the first dose (1.0 mg/kg, administered on Days 1 and 8, with a 3-week cycle) to reduce the number of patients exposed to ineffective or low-efficiency doses.

3.4.2. Selection Basis of Control Group

This study is a single-arm design without a control group.

3.4.3. Dose Escalation Method

For the dose escalation study, approximately 28 subjects will be enrolled. Dose escalation is performed by a combination of accelerated titration[1] and i3 +3[2] in a cohort of 2 to 4 subjects. Accelerated titration design will be used for the first dose group (only 1 subject will be enrolled):

a. If DLT or two grade≥2 drug-related AE (except infusion related reactions) are observed in the accelerated titration dose group, an additional 2 to 3 subjects will be added to the current dose level, and the subsequent study design will automatically be changed to an i3 +3 design of 2 to 4 subjects;

b. If no DLT is observed in the accelerated titration dose groups and the number of grade≥2 drug-related AE is less than two, the i3+3 design of 2 to 4 subjects will be used for dose escalation starting from the second dose group.

c. If safety events are observed in the accelerated titration dose group, the investigator evaluates that it is necessary to add subjects and continue to observe drug toxicity at the current dose level.

The i3 +3 design uses a Bayesian statistical framework and Beta-Bernoulli model to pre-calculate dose-increasing decision rules. The parameters of the i3 +3 design in

this study will adopt a target DLT rate of 28% and an equivalent interval of 23% ~ 33% (i.e., target DLT rate ±5%).

During the dose-escalation phase, dosing will be spaced at least 24 hours between enrolled subjects to allow for an initial assessment of tolerability and toxicity.

Stop dose escalation if any of the following conditions are met:

• Maximum sample size (approximately 55 cases in total);

• A sample size of ≥ 6 subjects treated at the same dose and the dose is determined to be MTD.

Note: Estimates of the maximum sample size are based on the number of subjects who are assumed to have climbed to the highest dose group and terminated the climb after full exposure to toxicity.

Based on the i3+3 design, the detailed rules of dose increase/decrease are shown in Table 1 and Table 2 below.

Table 1 i3 +3 dose increase/decrease rule (according to 3-subject cohort)

Total number of patients treated at current dose	*3*	*6*	*9*	*12*
Increase dose if number of DLT ≤	0	1	2	2
Dose reduction if number of DLT ≥	2	3	4	4
Terminate administration if number of DLT ≥	3	4	5	6

Note:

1. Termination of dosing represents the withdrawal of the current dose and all higher doses from the study for the purpose of protecting the subject. When termination occurs, the dose is automatically lowered to the next dose. If termination occurs at the lowest dose, the entire trial is stopped and MTD cannot be confirmed.
2. If no dose increase, dose decrease, or dose termination occurs, maintain the dose.
3. If the current dose is the minimum dose but the dose reduction is indicated in Table 1, the trial is stopped and MTD cannot be confirmed. If the current dose is the maximum dose, but the table indicates an increase in the dose, subsequent patients will be

maintained at the current maximum dose until the discontinuation condition is met.

Table 2 i3 +3 detailed dose increase/decrease rule

Number of Patients

Number of DLTs	1	2	3	4	5	6	7	8	9	10	11	12
0	E	E	E	E	E	E	E	E	E	E	E	E
1	S	S	S	S	E	E	E	E	E	E	E	E
2		DU	D	D	S	S	S	S	E	E	E	E
3			DU	DU	D	D	D	D	S	S	S	S
4				DU	DU	DU	DU	D	D	D	D	D
5					DU	DU	DU	DU	DU	D	D	D
6						DU	DU	DU	DU	DU	DU	DU
7							DU	DU	DU	DU	DU	DU
8								DU	DU	DU	DU	DU
9									DU	DU	DU	DU
10										DU	DU	DU
11											DU	DU
12												DU

Note 1: Target DLT rates pT=0.28, ε1=0.05, ε2=0.05, equivalent range 0.23~ 0.33.

Note 2: E Dose increase; S Maintain current dose; D Reduce to a previous dose; DU Lower to previous dose and cancel all doses above. Dose increases will only be triggered if the number of subjects in the group is ≥3, otherwise the current dose will be maintained.

After the dose escalation stopped in the dose increasing stage, Isotonic Regression is implemented by PAVA (Pool Adjacent Violators Algorithm) to estimate the probability of DLT occurrence in each dose group[4], Then, MTD is selected as the highest dose whose DLT rate estimate is closest to the target DLT rate, but does not exceed the upper limit of the equivalent interval of DLT rate.

Within the expected effective dose range, the investigator may adjust the magnitude of dose escalation, increase the intermediate dose and the rate of dose escalation, or perform a dose reduction climb, after communication with the sponsor medical personnel.

3.4.4. Administration regimen and definition of DLT and MTD

1）Administration Regimen

Phase Ia: BL-M07D1 will be administered intravenously. The infusion time of the first administration is 120 min±10min. If the infusion reaction is tolerable during the first administration, the infusion can be completed in 60-120 min±10min for subsequent administration. Cohort A: BL-M07D1 is administered on D1 and D8 every 3 weeks. Cohort B: BL-M07D1 is administered on D1 every 3 weeks. The administration will be terminated if disease progression or unacceptable toxicity or for other reasons (e.g. withdrawal of the Informed Consent Form or death). In the phase Ia (dose escalation phase), the administration schedule (including change of administration frequency, impact dose setting, etc.) and dose can be adjusted according to the obtained data of safety, tolerance, PK/PD, etc.

Phase Ib: According to the safety, pharmacokinetics and preliminary efficacy data of Phase Ia, the appropriate dose group and administration regimen will be selected for phase Ib study.

Before each administration of BL-M07D1, a complete blood count (CBC) test is required. Except for the first dose, neutrophils and platelets must meet the following criteria before each subsequent administration (for cohort B, only D1 dosing):

During the first cycle (DLT observation period), patients must meet the following criteria before each dose administration:

	Neutrophils≥	Platelets≥
D 0 or 1	1.5 x 10^9 /L	90 x 10^9 / L
D 7 or 8	1.5 x 10^9 / L	75 x 10^9 / L

In subsequent cycles, patients must meet the following criteria before each dose administration:

	Neutrophils≥	Platelets≥
D 0 or 1[1]	1.5 x 10^9 /L	75 x 10^9 / L

D 7 or 8[1]	1.0×10^9 / L	75×10^9 / L

Note1: In the Phase Ib expansion period, except for the first dose administration in Cycle 1, this standard shall apply to each subsequent dose administration.

2）Suspension, Termination, or Dose Adjustment

There are 3 dose reduction levels for each dose group of BL-M07D1. Refer to the following table for the initial dose group and dose reduction level of BL-M07D1

Table for BL-M07D1 dose reduction levels

Initial Dose Groups	Reduce one dose level	Reduce two dose levels
1.0 mg/kg D1D8	-	-
2.6 mg/kg D1	2.0 mg/kg D1	-
3.2 mg/kg D1	2.6 mg/kg D1	2.0 mg/kg D1
3.8 mg/kg D1	3.2 mg/kg D1	2.6 mg/kg D1
4.4 mg/kg D1	3.8 mg/kg D1	3.2 mg/kg D1
5.0 mg/kg D1	4.4 mg/kg D1	3.8 mg/kg D1
5.6 mg/kg D1	5.0 mg/kg D1	4.4 mg/kg D1
6.2 mg/kg D1	5.6 mg/kg D1	5.0 mg/kg D1
6.8 mg/kg D1	6.2 mg/kg D1	5.6 mg/kg D1
7.4 mg/kg D1	6.8 mg/kg D1	6.2 mg/kg D1

- Once the treatment dose is reduced due to toxicity, re-escalation of the dose is not allowed. After 2 dose reductions (except for the 1.0 mg/kg D1D8 and 2.6 mg/kg D1 dose levels), if toxicity requiring further dose reduction still occurs, the investigator may, based on the assessment of the subject's benefits and risks, either continue treatment for the patient at the dose "Reduce two dose levels" mentioned above or terminate treatment. If it is considered that the patient can still benefit from further dose reduction, the subsequent treatment dose may be discussed with

the sponsor. If toxicity requiring dose reduction still occurs after 1 dose reduction in the 2.6 mg/kg D1 dose level, the investigator may, based on the assessment of the subject's benefits and risks, either continue treatment for the patient at the dose "Reduce one dose level" or terminate treatment. If it is considered that the patient can still benefit from further dose reduction, the subsequent treatment dose may be discussed with the sponsor. If toxicity requiring dose reduction occurs in the 1.0 mg/kg D1D8 dose level, the investigator may discuss the subsequent dose reduction level with the sponsor based on the assessment of the subject's benefits and risks.

- During the DLT observation period, dose adjustments are not allowed, but for Cohort A, drug administration may be suspended, delayed, or discontinued. If there is a delay in drug administration, the DLT observation period will be extended accordingly (the maximum duration of the DLT observation period shall not exceed 28 days).
- If the drug administration is delayed for more than 28 days, the subject will withdraw from the study.

[redacted]

[redacted]

[redacted]	[redacted]
[redacted]	[redacted]
[redacted]	
[redacted]	[redacted]
[redacted]	[redacted]

[redacted]	[redacted]
[redacted]	[redacted]
[redacted]	[redacted]
[redacted]	
[redacted]	
[redacted]	[redacted]
[redacted]	[redacted]
[redacted]	[redacted]
[redacted]	[redacted]
[redacted]	
[redacted]	
[redacted]	[redacted]
[redacted]	
[redacted]	[redacted]
[redacted]	[redacted]
[redacted]	
[redacted]	[redacted]
[redacted]	[redacted]
[redacted]	[redacted]
[redacted]	
[redacted]	

[redacted]	[redacted]
[redacted]	
[redacted]	[redacted]
[redacted]	[redacted]
[redacted]	
[redacted]	[redacted]
[redacted]	[redacted]
[redacted]	
[redacted]	
[redacted]	[redacted]
[redacted]	
[redacted]	[redacted]
[redacted]	[redacted]
[redacted]	
[redacted]	

[redacted]	
[redacted]	[redacted]
[redacted]	
[redacted]	[redacted]
[redacted]	[redacted]
[redacted]	[redacted]
[redacted]	[redacted]
[redacted]	
[redacted]	[redacted]
[redacted]	[redacted]

[redacted]	[redacted]
	[redacted]<br>[redacted]<br>[redacted]
[redacted]	[redacted]
[redacted]	
[redacted]	[redacted]
[redacted]	
[redacted]	[redacted]
[redacted]	[redacted]
[redacted]	
[redacted]	
[redacted]	[redacted]
[redacted]	[redacted]<br>[redacted]
[redacted]	[redacted]
[redacted]	[redacted]<br>[redacted]
[redacted]	
[redacted]	[redacted]<br>[redacted]
[redacted]	[redacted]
[redacted]	

[redacted]	[redacted]
[redacted]<br>[redacted]	[redacted]<br>[redacted]<br>[redacted]<br>[redacted]<br>[redacted]
[redacted]<br>[redacted]	[redacted]<br>[redacted]<br>[redacted]<br>[redacted]<br>[redacted]
[redacted]	
[redacted]	[redacted]
[redacted]	[redacted]
[redacted]	
[redacted]	[redacted]<br>[redacted]<br>[redacted]
[redacted]	[redacted]
[redacted]	
[redacted]	[redacted]<br>[redacted]<br>[redacted]

[redacted]	[redacted]
[redacted]	[redacted]

The suspension, discontinuation of administration, or dosage adjustment shall be conducted in accordance with the above general principles. Investigators may, based on a comprehensive assessment of the risks and benefits to the subjects in the study in clinical practice, perform reasonable operations.

3） DLT Definition

The DLT observation period is the first treatment cycle for each dose level. If there is a dose delay, the DLT observation period shall be extended accordingly (with the maximum DLT observation period not exceeding 28 days). Toxicity should be graded according to NCI-CTCAE v5.0. During the DLT observation period, prophylactic medications that may affect the assessment of DLT are not permitted prior to the infusion of the study drug, with the exception of those used for nausea and vomiting, stomatitis, skin toxicity, or neutropenia. After the infusion of the study drug, supportive treatment is allowed if toxicity defined as grade ≥2 per CTCAE v5.0 occurs. DLT is defined as follows:

Hematological toxicities:

- After supportive care, Grade 4 neutropenia lasting >7 days; Grade≥3 febrile neutropenia;
- Grade 3 thrombocytopenia lasting >7 days after supportive care, or Grade ≥3 thrombocytopenia with significant bleeding;
- Grade 4 anemia lasting >7 days after supportive care.

Hepatic organ toxicities:

- Grade 4 elevated AST and/or ALT;
- AST and/or ALT > 5 × ULN, accompanied by Grade ≥2 elevated blood bilirubin;

- For patients without liver metastasis: AST and/or ALT > 5 × ULN for more than 3 consecutive days;
- For patients with liver metastasis: ① When baseline AST and/or ALT ≤ 3 × ULN, AST and/or ALT > 5 × ULN for more than 3 consecutive days; ② When baseline AST/ALT > 3 × ULN, AST/ALT > 8 × ULN for more than 3 consecutive days.

Other Grade ≥ 3 non-hematological, non-hepatic toxicities, except for:

- Grade 3 skin toxicity, mucositis, stomatitis, constipation, nausea, vomiting, anorexia, or electrolyte abnormalities, resolved to Grade≤2 (per CTCAE v5.0) within ≤7 days with symptomatic supportive care;
- Grade 3 diarrhea: resolved to Grade≤ 2 (per CTCAE v5.0) within 3 days with symptomatic supportive care;
- Grade 3 fatigue: resolved to Grade≤1 within 7 days after treatment;
- Asymptomatic Grade 3/4 laboratory abnormalities (e.g., elevated ALP, hyperuricemia, hyperglycemia).
- Any grade hair loss；
- Infusion-related Reactions (IRR).

Infusion-related reactions are generally the adverse reactions of blood products, protein products, biological agents that often occur during intravenous infusion and are not considered as DLT. However, if the patient develops a grade 3 or 4 infusion response and is unable to continue treatment with the study drug, an additional patient will be required in the dose group.

4）MTD Definition

In the dose-escalation phase, the highest dose of which estimated DLT rate is closest to the target DLT rate but does not exceed the upper bound of the DLT rate equivalence interval is selected as the MTD. If there are multiple dose groups with DLT rate estimates that all meet the above conditions, then:

1) If this estimate DLT rate is less than the target DLT rate, the highest of these doses is selected as the MTD;

2) If this estimate DLT rate is greater than the target DLT rate, the lowest of these doses is selected as the MTD.

3.4.5. Supplementary Subject

If during the first cycle of treatment, subjects drop out of the study for reasons other than DLT or voluntarily withdrew from the study, ① If the subject has completed the predicted total dose for DLT and completed at least 21 days of DLT observation, the subject is considered evaluable; ② If the subject has not completed all administration during the DLT period, or has not completed at least 21 days of DLT observation, the subject will be considered as unevaluable. Subjects who develop DLT during cycle 1 and drop out of the study are considered evaluable subjects. Supplementary rules for non-evaluable subjects are as follows:

① If the number of evaluable subjects and DLT in the current dose is determined as " Dose increase" or " Reduce to a previous dose" or " Lower to previous dose and cancel all doses above " according to the "Dose increase/decrease Rule ", the subject does not need to be supplemented at the current dose.

② If, in accordance with the "Dose Escalation/De-escalation Rules Table", the number of evaluable subjects and the number of DLT occurrences at the current dose are determined to "maintain the current dose", it is necessary to supplement this subject at the current dose or continue enrolling 2-4 additional subjects at this dose level.

4. Target Subject

The target population of this study consists of patients with locally advanced or metastatic HER2-positive/negative breast cancer and other solid tumors who have failed standard treatment or have no access to standard treatment.

4.1. Inclusion Criteria

1. Signs the informed consent voluntarily and follow the protocol requirements;
2. Either Sex;
3. Age: ≥18 years and ≤75 years (phase Ia);
   Age: ≥18 years (phase Ib);
4. Has a life expectancy of ≥ 3 months;
5. Patients with unresectable locally advanced or metastatic HER2-positive/negative breast cancer and other solid tumors, confirmed by histopathology and/or cytology, who have failed standard treatment, have no access to standard treatment regimens, or are not eligible for standard treatment at the current stage;
   HER2-positive: IHC 3+, or IHC 2+ with ISH-positive;
   HER2-negative: IHC 2+ with ISH-negative, IHC 1+, or IHC 0;
6. Subjects must agree to provide archived tumor tissue specimens (within 2 years) or fresh tissue samples from the primary or metastatic lesion. These samples will be used to detect HER2 protein expression in tumor pathological tissue and explore the correlation between this expression and the efficacy indicators of BL-M07D1. If a subject is unable to provide tumor tissue samples, they may still be enrolled after investigator assessment, provided they meet other inclusion/exclusion criteria. However, subjects with HER2 IHC 0 must provide such samples.
7. Has at least one measurable lesion based on RECIST V1.1;
8. Has an Eastern Cooperative Oncology Group performance status (ECOG PS) 0-1;
9. Toxicity of previous antitumor therapy has returned to grade ≤1 as defined by NCI-CTCAE V5.0 (except for asymptomatic laboratory abnormalities at the discretion of investigator, such as elevated ALP, hyperuricemia, and elevated blood glucose; except for toxicity that the investigator determined to have no safety risk, such as alopecia, hyperpigmentation, grade 2 peripheral neurotoxicity etc.);
10. Has not serious cardiac dysfunction, left ventricular ejection fraction ≥50%;

11. Within 14 days prior to the first administration of the study drug, subjects must not have received blood transfusions, not have used colony-stimulating factors, any cell growth factors, or other injectables, and not have used albumin. On this premise, their organ function must meet the following requirements and standards:
    - Marrow Function: Absolute neutrophil count (ANC) ≥1.5×10^9/L, Platelet count ≥90×10^9/L, Hemoglobin (Hb) ≥90 g/L;
    - Hepatic function: Total bilirubin (TBIL≤1.5 ULN), AST and ALT (without liver metastasis) ≤2.5 ULN, AST and ALT (with liver metastasis) ≤5.0 ULN; Albumin≥30 g/L；
    - Renal function: Creatinine (Cr)≤1.5 ULN, or creatinine clearance (Ccr) ≥ 50 mL/min (According to the Cockcroft and Gault).
12. Coagulation function: international normalized ratio (INR)≤1.5×ULN, and activated partial thromboplastin time (APTT)≤1.5ULN;
13. Urinary protein ≤2+ or ≤1000mg/24h;
14. For premenopausal women with childbearing potential, a pregnancy test must be taken within 7 days prior to the start of treatment. Serum or urine pregnancy must be negative and must be non-lactating. Adequate barrier contraceptive measures should be taken during the treatment and 6 months after the end of treatment for all participants (regardless of male or female).

4.2. Exclusion Criteria

Patients screened for any of the following conditions are excluded from the study:

1. Chemotherapy, biological therapy, immunotherapy, radical radiotherapy, major surgery (defined by the investigator), targeted therapy (including small molecule inhibitor of tyrosine kinase), and other anti-tumor therapy within 4 weeks or 5 half-lives (whichever is shorter) prior to the first administration; mitomycin and nitrosoureas treatment within 6 weeks prior to the first administration; oral fluorouracil-like drugs such as S-1, capecitabine, or palliative radiotherapy within

2 weeks prior to the first administration; For traditional Chinese medicines (TCMs) with anti-tumor indications, within 2 weeks prior to the first dose administration.

2. Having received prior treatment with ADC drugs conjugated with camptothecin derivatives (topoisomerase I inhibitors) as toxins (Phase Ib only);
3. Participants with history of severe heart disease, such as: symptomatic congestive heart failure (CHF) ≥ grade 2 (CTCAE 5.0), New York Heart Association (NYHA) ≥ grade 2 heart failure, history of transmural myocardial infarction, unstable angina pectoris etc;
4. Participants with prolonged QT interval (male QTc> 450 msec or female QTc> 470 msec), complete left bundle branch block, III grade atrioventricular block;
5. Active autoimmune diseases and inflammatory diseases, such as: systemic lupus erythematosus, psoriasis requiring systemic treatment, rheumatoid arthritis, inflammatory bowel disease and Hashimoto's thyroiditis, etc., except for type I diabetes, hypothyroidism that can be controlled only by alternative treatment, and skin diseases that do not require systemic treatment (such as vitiligo, psoriasis);
6. Other malignant tumors were diagnosed within 5 years prior to the first administration with the following exceptions: basal cell carcinoma of the skin, squamous cell carcinoma of the skin and/or carcinoma in situ after radical resection;
7. Unstable thrombotic events such as deep vein thrombosis, arterial thrombosis, and pulmonary embolism requiring therapeutic intervention within 6 months prior to screening; Thrombus formation associated with infusion set is excluded;
8. Patients with massive serous cavity effusion, or symptomatic serous cavity effusion, or poorly controlled serous cavity effusion (defined as requiring 2 or more puncture drainages within 1 month);
9. Participants with poorly controlled hypertension by two kinds of antihypertensive drugs (systolic blood pressure>150 mmHg or diastolic blood pressure>100 mmHg);
10. Patients with pulmonary diseases defined as grade ≥3 per CTCAE v5.0, radiation pneumonitis of grade ≥2, or current or a history of interstitial lung disease (ILD);

11. Having active symptoms of central nervous system (CNS) metastasis. However, patients with stable brain parenchyma metastasis may be enrolled, as deemed by the investigator. The definition of "stable" must meet all four of the following criteria:

    a. No seizure activity for more than 12 weeks, with or without the use of antiepileptic drugs;

    b. No requirement for glucocorticoid use;

    c. Imaging stability confirmed by two consecutive MRI scans (with an interval of at least 4 weeks between scans);

    d. Asymptomatic status for more than 1 month after treatment;
12. Participants who have a history of allergies to recombinant humanized antibodies or human-mouse chimeric antibodies or any of the components of BL-M07D1;
13. Participants have a history of autologous or allogeneic stem cell transplantation (Allo-HSCT);
14. Having received prior anthracycline treatment with a cumulative doxorubicin-equivalent dose exceeding 360 mg/m^2;
15. Human immunodeficiency virus antibody (HIVAb) positive, active tuberculosis, active hepatitis B virus infection (HBV-DNA copy number> lower limit of detection) or active hepatitis C virus infection (HCV antibody positive and HCV-RNA > the lower limit of detection);
16. Participants with active infections requiring systemic treatment, such as severe pneumonia, bacteremia, sepsis, etc;
17. Having participated in another clinical trial within 4 weeks prior to the first administration of the study drug (calculated from the date of the last dose). For clinical trials involving marketed drugs or drugs with a known half-life, refer to exclusion criterion 1;
18. Women who are pregnant or breastfeeding;
19. Other conditions that the investigator believes that it is not suitable for participating in this clinical trial.

4.3. Study Duration

Cohort A: BL-M07D1 is administered on D1 and D8 every 3 weeks. Cohort B: BL-M07D1 is administered on D1 every 3 weeks. The administration will be terminated if disease progression or unacceptable toxicity or for other reasons (e.g. withdrawal of the Informed Consent Form or death).

4.4. Early Termination or Suspension Enrollment of Study

The sponsor reserves the right to terminate the participation of a research center or the entire study at any time and for any reason. Reasons for termination of the study include but are not limited to the following:

1. Dose escalation studies are discontinued after MTD or expected biological effects are achieved and safety is comprehensive considered.
2. Serious safety problems are found in the trial, and the researchers and sponsors agree that continuing the study carried serious risks;
3. The trial should be terminated in time in order to protect the rights and interests of the subjects and avoid unnecessary economic losses if the drug is found to be of no clinical value during the trial;
4. Major errors are found in the design of the trial, which makes it difficult to evaluate the drug, or there are major deviations in the implementation of the study protocol, which affects the final evaluation of the drug during the trial;
5. The sponsor has decided to suspend or terminate this clinical study or the development of BL-M07D1.

All investigators and appropriate regulatory authorities should be notified immediately of the decision to terminate the study early.

If two unexplained deaths are observed, possibly related to the experimental drug, we will discontinue enrollment. We will immediately meet with the investigator to

review each case and decide whether to revise the study plan, terminate or continue the study.

4.5. Patient Withdrawal from Treatment Criteria

The patient will withdraw from treatment if any of the following conditions occur and will be noted in the medical record during the trial.

1. The patient requests to withdraw informed consent;
2. The investigator determines that treatment should be terminated in the patient's best interest;
3. Occurrence of intolerable adverse reactions that meet the criteria for treatment discontinuation;
4. Evidence of disease progression: After disease progression, if the investigator assesses that continued treatment is beneficial to the subject and the subject is willing to continue, the subject may continue treatment;
5. The patient becomes pregnant during the study;
6. Poor patient compliance or major protocol violations that affect the evaluation of drug tolerability, safety, or pharmacokinetics;
7. Use of other anti-tumor drugs for treatment;
8. The patient is lost to follow-up;
9. The patient dies;
10. Other reasons.

In addition to patients requesting withdrawal of informed consent, loss of follow-up and death, follow-up should be completed as far as possible for those who quit treatment above.

4.6. Definition of Patient Completion of The Study

Patients completing the study is defined as disease progression (PD) as assessed by RECIST V1.1 is observed and completing follow-up visits; Dose-limiting toxicity

and follow-up are completed before disease progression is observed; Death is also considered completion of the study. Reasons for early withdrawal from the study include:

1. The patient requests to withdraw informed consent;
2. Adverse events occur;
3. The patient is lost to follow-up;
4. No disease progression occurs after 24 months of BL-M07D1 administration;
5. The sponsor decides to terminate the study, with no further follow-up conducted;
6. The investigator and/or sponsor deems the patient's compliance to be poor;
7. The research center where the patient is enrolled terminates its participation in the trial;
8. Other reasons.

5. Investigational Product

5.1. Information on Investigational Product

【Investigational Product】

Chinese name: 注射用 BL-M07D1

English name: BL-M07D1 for Injection

Chinese phonetic: Zhusheyong BL-M07D1

Generic name: Pending Requests

Product name: Pending Requests

【Ingredient】

Active ingredient: Antibody-drug conjugate BL-M07D1

【Characteristics】[redacted]

【Stability and Storage Conditions】[redacted]

【Specification】[redacted]

【Packing】[redacted]

【Preparation】[redacted]

5.2. Preparation of Investigational Product

5.2.1. Preparation of BL-M07D1 for Injection

[redacted]

[redacted]

[redacted]

[redacted]

[redacted]

[redacted]

[redacted]

5.2.2. Recommended of Infusion Rate

The prepared injection solution should be a colorless to pale yellow liquid. Once the infusion of this product is ready, administration is recommended as soon as possible. The product will be administered via intravenous infusion through a peripheral venous or central venous catheter. The first infusion of BL-M07D1 for injection should last 120 minutes ± 10 minutes; if the infusion reaction is tolerable during the first administration, subsequent infusions can be completed within 60-120 minutes.

5.2.3. Precautions

(1) The preparation process of the test drug should be carried out under class A conditions such as biosafety cabinet, ultra-clean workbench or Class A laminar flow cover. If the liquid preparation under class A conditions cannot be met, microbial contamination should be prevented to the maximum extent;

(2) When preparing the study drug, sterile water for injection should be added at a volume of 2.5 ml per vial for reconstitution. The drug can only be diluted with 0.9% sodium chloride injection and must not be mixed with other drugs or diluted with other drugs or solvents.

(3) The preparation process should be gentle, not violent shock, should try to avoid foam. If the foam is generated, it should be left standing until the foam disappears;

(4) It is recommended to infuse the product immediately after preparation. If immediate infusion is not possible, when preparing the solution at room temperature, the total time from removing the product from 2–8°C, starting preparation, to completing the infusion shall not exceed 6 hours.

(5) If adverse reactions occur during the infusion, prompt management is required,

and the remaining unused infusion solution must be immediately stored at -20°C. After discussion between the investigator and the sponsor, a decision will be made on whether to seal the remaining unused solution and send it back to the sponsor. If return is required, the solution shall be sent back to the sponsor under conditions of -20°C or below; if the remaining unused solution is not stored at the specified temperature after the occurrence of adverse reactions, there is no need to send the remaining solution back to the sponsor.

(6) In case of turbidity, precipitation and other problems, the injection should be suspended and sealed up, and immediately notify the sponsor;

(7) Although this product has high safety in animal experiments, it should still avoid drug exudation.

5.3. Supply, Packaging, Labelling and Storage of Investigational Product

Sponsor to provide sufficient study drugs for the study period. These investigational drugs will be packaged in accordance with the study requirements, provided to the research center by the sponsor, and received by authorized participants. The center will store all investigational drugs as required.

Information such as study number, drug number, batch number, specification, storage conditions, production date, expiration date, name of sponsor, etc. shall be printed on the package of investigational drugs, and "for clinical investigational drugs only" shall be indicated.

5.4. Reception, Distribution and Custody of the Investigational Drug

The sponsor will provide the study drug in sufficient quantities to ensure the study is completed. The receipt, storage, distribution and counting of all investigatory drugs

will be supervised by authorized personnel in accordance with GCP requirements. The study drug will be placed in a restricted access area and stored at 2~8°C away from light.

It is the responsibility of the investigator to maintain accurate count records of the investigational drug during the clinical study. Each patient's medication status will be recorded in the original medical record and electronic Case Report Form (eCRF).

5.5. Randomness and Blindness

Do not apply. An open, single-arm design will be used in this study.

5.6. Concomitant Medications Before and During the Study

5.6.1. Drugs not to be Used during the Study

The concomitant use of anti-tumor therapy (including chemotherapy, immunotherapy, radiotherapy, biological response modifiers, Chinese medicines with anti-tumor indications, etc.) and other investigational drugs is prohibited during the study period, and palliative radiotherapy for bone pain relief is allowed during the study period.

5.6.2. Medications Allowed to be Used During the Study

Patients may receive palliative and supportive care for tumor-related symptoms and treatment-related toxicities of the study drug, which may include (but is not limited to): antiemetics, opioid or non-opioid analgesics, and granulocyte and erythrocyte growth factors. For potential nausea and vomiting in patients, prophylactic medications may be administered before drug administration based on clinical practice or with reference to the *2019 Chinese Expert Consensus on the Prevention and Treatment of Nausea and Vomiting Related to Antineoplastic Drug Therapy* [11], such as serotonin

receptor antagonists combined with hormonal drugs (dexamethasone); for potential oral mucositis, prophylactic medications may be given before administration based on clinical practice or with reference to the *2021 Expert Consensus on the Diagnosis, Prevention and Treatment of Acute Oral Mucositis Caused by Antitumor Therapy* [12]; and for potential skin toxicities, prophylactic medications may be used before administration based on clinical practice or with reference to the *2021 Clinical Practice Guidelines for the Prevention and Management of Anticancer Drug-Related Skin Toxicities* [13]. Additionally, patients are allowed to continue long-term medications required for comorbid conditions (e.g., patients with bone metastases may receive long-term bisphosphonates for bone metastasis treatment; if bone metastasis lesions with pain cannot be effectively controlled by systemic therapy or local analgesia, palliative radiotherapy for small areas is permitted, with the radiotherapy field must be < 5% of the bone marrow area).

[redacted]

[redacted]

[redacted]

[redacted]

[redacted]

[redacted]	[redacted]
	[redacted]
	[redacted]
	[redacted]
[redacted]	[redacted]

[redacted]

[redacted]

[redacted]

[redacted]

[redacted]

[redacted]

[redacted]

[redacted]

[redacted]

[redacted]

[redacted]	[redacted]
[redacted]	

	[redacted]
[redacted]	[redacted]

[redacted]

[redacted]

[redacted]

[redacted]

[redacted]

[redacted]

[redacted]

[redacted]

[redacted]

[redacted]

[redacted]

[redacted]

[redacted]

[redacted]

[redacted]

[redacted]		
[redacted]		[redacted]
[redacted]	[redacted]	[redacted]
	[redacted]	

[redacted]

[redacted]

[redacted]

[redacted]

[redacted]

[redacted]

[redacted]

[redacted]

[redacted]	[redacted]
[redacted]	[redacted]
[redacted]	[redacted]

	[redacted]
[redacted]	[redacted]

	[redacted]

[redacted]

[redacted]			
	[redacted]		
[redacted]	[redacted]	[redacted]	[redacted]
[redacted]		[redacted]	[redacted]
[redacted]	[redacted]	[redacted]	[redacted]

[redacted]

[redacted]

[redacted]

[redacted]

[redacted]

[redacted]

[redacted]

[redacted]

[redacted]

[redacted]

[redacted]

[redacted]

[redacted]

[redacted]

[redacted]

[redacted]

[redacted]

[redacted]

[redacted]

[redacted]

[redacted]	[redacted]	[redacted]					
			[redacted]				
		[redacted]	[redacted]			[redacted]	[redacted]
[redacted]	[redacted]	[redacted]	[redacted]	[redacted]	[redacted]	[redacted]	[redacted]

		[redacted]			[redacted]	[redacted]	
[redacted]	[redacted]	[redacted]	[redacted]	[redacted]			
[redacted]	[redacted]		[redacted]	[redacted]	[redacted]		[redacted]
[redacted]	[redacted]		[redacted]	[redacted]			
[redacted]							

[redacted]

[redacted]

[redacted]

[redacted]

[redacted]

[redacted]

[redacted]		[redacted]	[redacted]	[redacted]
[redacted]	[redacted]	[redacted]	[redacted]	[redacted]

			[redacted]	
[redacted]	[redacted]	[redacted]<br>[redacted]<br>[redacted]	[redacted]<br>[redacted]	[redacted]<br>[redacted]<br>[redacted]<br>[redacted]

			[redacted]	
[redacted]		[redacted]	[redacted]	[redacted]
[redacted]		[redacted]	[redacted]	[redacted]

		[redacted]		[redacted]

During the study period, specific information about concomitant medications administered to subjects and details of palliative/supportive care (e.g., blood transfusion) must be documented in the subjects' medical records and reported in the Case Report Form (CRF).

6. STUDY PROCEDURES

6.1 Phase Ia Study Procedures

6.1.1 Visiting 1: Screening (Day-28 ~ Day-1)

- Screening number assignment;
- Collect demographic information;
- Obtain medical history and prior treatment;
- Vital sign examination;
- ECOG Performance Status assessment;
- Record height, weight (During Day-7 to Day -1);
- Physical examination;
- Serum virology and HIV antibody test;
- 12-Lead ECG (Day -7 ~ Day -1);
- Echocardiogram;

- Laboratory examination: Blood routine, Serum biochemistry, coagulation, markers of myocardial damage, Urinalysis, stool routine and occult blood (Day -7 ~ Day -1)；
- Pregnancy test (Day -7 ~ Day -1)；
- Tumor assessment (Including tumor markers),and whole body osteonuclide scan (when necessary)；
- Obtain tumor tissue sample；
- Record concomitant medications and treatment；
- Record SAE before administration；
- Assess inclusion/exclusion criteria.

6.1.2 Visiting 2 (Cycle 1 Day 1,administration)

- Review inclusion and exclusion criteria before drug administration;
- Vital sign: ECG monitoring is performed from 30min before administration to 30min after administration, and recorded every 30min±10min. Vital signs are recorded at 2 h±30 min, 4 h±30 min and 6 h±30 min after administration, respectively. If the vital signs are unstable after administration, the ECG monitoring could be extended until stable;
- Before administration, perform physical examination；
- Before administration, assess ECOG；
- Before administration, weight (The time window is -1 day, and dosage will be calculated according to the weight)；
- 12-Lead ECG (perform ECG monitoring when necessary): ECG examinations are performed 1 hour before administration, 30 min±10min, 2 h±30 min, 4 h±30 min, 6 h±30 min after administration.
- Collect pharmacokinetic blood samples (See Section 8. Pharmacokinetic studies)；
- Administer BL-M07D1 intravenously；

- Before administration, collect blood samples for immunogenicity test;
- Record concomitant medications and treatment;
- Record AEs。

6.1.3 Visiting 3,4 (Cycle 1 Day 2,3)

- Vital sign;
- Physical examination;
- ECOG Performance Status assessment;
- 12-Lead ECG (perform ECG monitoring when necessary);
- Record concomitant medications and treatment;
- Record AEs.

6.1.4 Visiting 5-8 (Cycle 1 Day 8, 15 and 22)

- Vital sign: ECG monitoring is performed from 30min before administration to 30min after administration, and recorded every 30min±10min. If there is a clinical indication, the investigator may decide whether to add vital sign monitoring time;
- ECOG Performance Status assessment;
- Physical examination;
- Before Cohort A administration, weight (The time window is -1 day, and dosage will be calculated according to the weight);
- 12-Lead ECG (perform ECG monitoring when necessary): ECG examination is performed at 1 hour before administration and 30min±10min after administration. If there is a clinical indication, the investigator may decide whether to add 12-Lead ECG examination;
- Laboratory examination before administration: blood routine, Serum biochemistry, coagulation, markers of myocardial damage, Urinalysis, stool routine and occult blood (C1D22);

- Echocardiogram (perform on C1D22±7 days);
- Cohort A: administration on D1 and D8, 3-week cycle; BL-M07D1 intravenous administration (day 8);
- Collect pharmacokinetic blood samples(see Section 8. Pharmacokinetic studies);
- Record concomitant medications and treatment;
- Record AEs.

6.1.5 Visiting 7-10 (Cycle 2 Day 1, 8, 15 and 22)

- Vital sign: ECG monitoring is performed from 30min before administration to 30min after administration, and recorded every 30min±10min. If there is a clinical indication, the investigator may decide whether to add vital sign monitoring time;
- ECOG Performance Status assessment (before each administration);
- Physical examination;
- Before administration, weight (The time window is -1 day, and dosage will be calculated according to the weight);
- 12-Lead ECG (perform ECG monitoring when necessary): ECG examination is performed at 1 hour before administration and 30min±10min after administration. If there is a clinical indication, the investigator may decide whether to add 12-Lead ECG examination;
- Laboratory examination before administration: blood routine, Serum biochemistry (C2D1, C2D8, C2D15, C2D22), coagulation (C2D1, C2D8, C2D22), markers of myocardial damage, Urinalysis, stool routine and occult blood (C2D1, C2D22);
- Echocardiogram (perform on C2D22±7 days);
- Cohort A: administration on D1 and D8, 3-week cycle; BL-M07D1 intravenous administration (day 1 and 8); Cohort B: administration on D1, 3-week cycle; BL-M07D1 intravenous administration (day 1);
- Collect pharmacokinetic blood samples(see Section 8. Pharmacokinetic studies);
- Record concomitant medications and treatment;

- Record AEs.

6.1.6 Subsequent Cycles

Examination will be performed in subsequent cycles: **Cohort B will not undergo subsequent Day 8 visits in the following cycles.**

- Vital sign: ECG monitoring is performed from 30min before administration to 30min after administration, and recorded every 30min±10min. If there is a clinical indication, the investigator may decide whether to add vital sign monitoring time;
- Record weight (Before each administration, the next dose will be calculated according to the current body weight, and the time window is -1 day)；
- Physical examination (Before each administration)；
- ECOG assessment (Before each administration)；
- 12-Lead ECG (perform ECG monitoring when necessary) ECG examination is performed at 1 hour before administration and 30min after administration. If there is a clinical indication, the investigator may decide whether to add 12-Lead ECG examination；
- Echocardiogram (performed on the Day 22±7 days of each even-numbered cycle beginning with cycle 2)；
- Laboratory examination: Blood routine, Serum biochemistry, coagulation (before each administration), Urinalysis, stool routine and occult blood test, markers of myocardial damage (Before the first administration of each cycle)；
- Tumor assessment (including serum tumor markers): Duration of administration: Tumor evaluation is performed once every 6 weeks ±7 days in the first year, and once every 12 weeks ±7 days in the second year. Tumor assessment is performed every 12 weeks ±7 days in the first year after the last administration. Tumor assessment is performed every 24 weeks ±7 days in the second year after the last administration.
- Cohort A：administration on D1 and D8, 3-week cycle; BL-M07D1 intravenous

administration (day 1 and 8); Cohort B: administration on D1, 3-week cycle; BL-M07D1 intravenous administration (day 1);

- Collect blood samples for immunogenicity test before administration (Before the first administration in each even-numbered cycle (within -4h))；
- Collect pharmacokinetic blood samples (see Section 8. Pharmacokinetic studies)；
- Record concomitant medications and treatment (continuously)；
- Record AEs (continuously).

6.1.7 End of treatment visit

End of treatment visits should be conducted within 0-7 days of disease progression with the following tests:

- Vital sign；
- ECOG Performance Status assessment；
- Record weight;
- Physical examination；
- 12-Lead ECG (perform ECG monitoring when necessary);
- Echocardiogram;
- Laboratory examination: blood routine, Serum biochemistry, coagulation, markers of myocardial damage, Urinalysis, stool routine and occult blood;
- Serum virology and HIV testing;
- Collect blood samples for immunogenicity test;
- Pregnancy test;
- Tumor assessment (Including serum tumor markers; If subjects discontinue treatment for reasons other than disease progression, imaging studies will be required to assess efficacy);
- Record concomitant medications and treatment;

- Record AE.

6.1.8 Safety Follow-up

A safety visit should be conducted 28 (+7) days after the treatment discontinuation visit to follow up on drug-related adverse events present at the treatment discontinuation visit and collect newly occurring adverse events. Adverse events should be followed up until they stabilize or return to baseline. If the patient initiates a new anti-tumor treatment within 28 (+7) days after the treatment discontinuation visit, the safety visit is not required.

The examination items are as follows:

- Vital signs;
- ECOG performance status assessment;
- Record Body weight;
- Physical examination;
- 12-lead ECG examination;
- Laboratory tests: blood routine, blood biochemistry, coagulation function, myocardial injury markers, urine routine, stool routine + occult blood test;
- Pregnancy test;
- Recording of concomitant medications and treatments;
- Recording of adverse events.

6.1.9 Follow-up Visit

For patients who discontinue treatment due to reasons other than disease progression, tumor efficacy assessments will be conducted once every 12 weeks ±7 days in the first year after the safety visit, and once every 24 weeks ±7 days in the second year. This will continue until the patient initiates a new anti-tumor drug treatment, experiences disease progression, withdraws informed consent, drops out, is

lost to follow-up, or dies—whichever occurs first. Subsequent survival follow-up for these patients will be performed once every 8 weeks ±7 days, or as arranged by the investigator based on the patient's actual condition. Follow-up will end 12 months after the last patient completes the study, or when the patient dies or is lost to follow-up—whichever occurs first.

For patients who discontinue treatment due to disease progression, survival follow-up will start after the safety visit or the initiation of a new anti-tumor drug treatment. The follow-up frequency will be once every 8 weeks ±7 days, or as arranged by the investigator based on the patient's actual condition. Follow-up will end 12 months after the last patient completes the study, or when the patient dies or is lost to follow-up—whichever occurs first.

6.2 Phase Ib Study Procedures

In the phase Ib, appropriate dose groups and administration regimens will be selected based on the safety, pharmacokinetic, and preliminary efficacy data from the phase Ia to conduct the phase Ib study. The study procedures and processes (screening period and administration methods) in the phase Ib will be the same as those in the corresponding steps of the phase Ia.

7 Safety and Tolerance Assessments

7.1 Vital signs and body weight

Vital signs include respiratory rate, pulse, systolic and diastolic blood pressure, and body temperature.

A complete physical examination including height and weight will be performed at screening. Only body weight is measured during the subsequent administration period, and the dose is calculated based on the body weight before each administration.

7.2 Physical Examination

The complete physical examination includes: subjects' general condition, head and face, cutaneous system, lymph nodes, eyes, otolaryngological region, oral cavity, respiratory system, cardiovascular system, abdomen, genitourinary system, musculoskeletal system, nervous system, and mental state. Any abnormalities found during the baseline period should be recorded on the eCRF's general history and Baseline status page. At follow-up visits, if clinically indicated, a targeted physical examination should be performed, and any abnormalities from baseline should be recorded in the patient's course. New or worsening clinically significant abnormalities should be recorded as adverse events on the eCRF's Adverse Events page.

7.3 Laboratory Examination

Blood routine test (reticulocyte, hemoglobin, red blood cell count, white blood cell count, neutrophil count, lymphocyte count, eosinophil count, basophil count, monocyte count, and platelet count).

Serum biochemistry (alanine aminotransferase, aspartate aminotransferase, total bilirubin, direct bilirubin, alkaline phosphatase, total protein, albumin, urea, creatinine, fasting blood glucose, total cholesterol, triglyceride, lactate dehydrogenase, electrolytes (including sodium, potassium, magnesium, chloride, calcium, phosphorus) etc.).

Coagulation function tests [prothrombin time (PT), activated partial thrombin time (APTT), thrombin time (TT), fibrinogen (FIB) and international standardized ratio (INR)].

Serum/urine pregnancy tests (women of childbearing potential, including those with a history of tubal ligation).

Myocardial injury markers: myoglobin (Mb), B-type natriuretic peptide (BNP), creatine kinase isoenzyme Mb (CK-MB), cardiac troponin (cTn). Creatine kinase isoenzyme MB (CK-MB) examination is not required according to the actual situation

of the center.

7.4 ECG

The ECG should be performed only after the patient fully rests in supine position. All ECG parameters are recorded, including, but not limited to, heart rate, P-R interval, QRS interval, QT interval, QTc interval, assessment of QRS and T wave morphology, and the investigator's assessment of the ECG curve.

7.5 Echocardiogram

Cardiac, great vessel organic structure (such as heart size, valves, etc.), pericardium and valvular function and left ventricular ejection fraction (LVEF) would be detected.

8 Pharmacokinetic study

8.1 Phase Ia Pharmacokinetic study

Cohort A: drug administration on D1 and D8 with a cycle every 3 weeks. Blood is collected according to Table 1. Cohort B: D1 administration, one cycle every 3 weeks. Blood is collected according to Table 2. Each blood collection is about 4mL. In phase Ia, the pharmacokinetics of ADC, total antibody and small molecule toxin are studied. Among them, about 2mL of blood is collected each time for analyzing ADC and total antibody, and about 2mL of blood is collected each time for analyzing small molecule toxins. In the dose escalation phase, based on the preliminary pharmacokinetic data obtained from the dose groups with escalation completed, the blood collection time of patients in the subsequent dose group may be adjusted accordingly.

Table 1. D1, D8 administration, a cycle every 3 weeks PK blood collection flow chart

Blood Collection Timepoints	Cycle 1 (21 days)										Subsequent Cycles		
	1	2	3	5	8	9	10	12	15	22	1	8	22
Before 1st administration - within 4 hours	X										X		
Immediately after 1st dosing (+within 15 minutes).	X										X		
2 hours ±30 min after the end of 1st administration	X												
4 hours ±30min after the end of 1st administration	X												
6 hours ±30min after the end of 1st administration	X												
24 hours ±1 hour (D2) after the end of 1st administration		X											
48 hours ±2 hours (D3) after the end of 1st administration			X										
96 hours ±4 hours (D5) after the end of 1st administration				X									
after the end of 1st dosing within 168 hours to 4 hours (Day 8) [2]/ within 4 hours before 2nd administration (Day 8)					X							X	
End of 2nd dosing immediately (+within 15 minutes).					X							X	
2h±30min after the end of 2nd administration					X								
4 hours ±30min after the end of 2nd administration					X								
6 hours ±30min after the end of 2nd administration					X								
24 hours ±1 hours (Day 9) after the end of 2nd administration						X							
48 hours ±2 hours (Day 10) after the end of 2nd administration							X						
96 hours ±4 hours (Day 12) after the end of 2nd administration								X					
168 hours ±4 hours (Day 15) after the end of 2nd administration									X				
within 336 hours -4 hours (Day 22) after the end of 2nd administration										X [1]			X [1]

Note:

1. If the patient receives administration in the next cycle, the blood collection before Day 1 administration of the next cycle overlaps with this blood collection time point, and only one blood collection is required. If the patient no longer receives treatment in the next cycle, the PK blood collection on Day 22 of this cycle must

be completed.

2. If the patient receives the second administration in Cycle 1, the blood collection before the second administration of Cycle 1 overlaps with the blood collection time point on Day 8 of the first administration in this cycle (within 168 h - 4 h after the end of the 1st administration), and only one blood collection is required. If the patient no longer receives the second administration in Cycle 1, the PK blood collection on Day 8 of the first administration in this cycle (within 168 h - 4 h after the end of the 1st administration) must be completed.

Table 2. D1 administration, a cycle every 3 weeks PK blood collection flow chart

Blood Collection Timepoints	Cycle 1 (21 days)							Cycle 2 (21 days)				Subsequent Cycles	
	1	2	3	5	8	15	22	D1	D8	D15	D22	1	22
Before administration - within 4 hours	X							X				X	
Immediately after dosing (+15 minutes)	X							X				X	
2 hours after the end of administration ±30 minutes	X												
4 hours ±30 minutes after the end of administration	X												
6 hours after the end of the drug administration±30 minutes	X												
24 hours after the end of administration ±1 hour (Day 2)		X											
48 hours ±2 hours after the end of dosing (Day 3)			X										
96 hours ±4 hours after the end of dosing (Day 5)				X									
168 hours ±4 hours after the end of dosing (Day 8)					X				X				
336 hours ±4 hours after the end of dosing (Day 15)						X				X			
Within 504 hours -4 hours after the end of administration (Day 22)							X [1]				X[1]		X [1]

Note:

1. If the patient proceeds with the next cycle of administration, the blood collection prior to Day 1 (D1) administration of the next cycle will coincide with this blood collection time point, and only one blood collection is required. If the patient does not continue with the next cycle of treatment, the pharmacokinetic (PK) blood collection on Day 22 (D22) of the current cycle must be completed.

8.2 Phase Ib Pharmacokinetic study

The blood sampling time points in the expanded enrollment phase refer to those of the pharmacokinetic (PK) study in the dose escalation phase. Specifically, only the first three subjects in the 6.2 mg/kg and higher dose groups at the leading site need to undergo blood sampling at all time points specified in the PK study. For other expanded subjects at the leading site and all expanded subjects at the sub-sites, blood sampling will only be retained at two time points: within 4 hours before drug administration and immediately after drug administration (within +15 minutes). The blood sampling time points in the expanded enrollment phase may be adjusted based on the results of the dose escalation phase.

8.3 Collection, processing and storage of biological samples

After collecting PK whole blood samples, serum should be separated and aliquoted in accordance with the requirements of the laboratory manual, then stored in a refrigerator at below -60°C. It is important to note that the collection date and exact time of each blood sample from all patients participating in the study must be recorded in the electronic Case Report Form (eCRF), with notes on any issues encountered during sample collection (e.g., deviation from the scheduled sampling time).

Serum levels of anti-drug antibodies (ADA) and neutralizing antibodies (Nab) will be tested at the following time points:

Before the first dose of the first treatment cycle;

Starting from the second treatment cycle, before the first infusion of the study drug in each even-numbered cycle (within -4 hours);

Within 0–7 days after disease progression, following the completion of all treatment cycles.

Approximately 3 mL of blood will be collected for each sample.

8.4 Cold chain system for biological sample transport

Send samples to central laboratory for analysis according to laboratory manual.

9 Immunogenicity evaluation

The immunogenicity evaluation outcome included anti-drug antibody (ADA) of BL-M07D1. The effects of neutralizing antibodies on responses to BL-M07D1, pharmacokinetics and pharmacodynamics may be analyzed.

The production of ADA and neutralizing antibody (Nab) in serum is detected at the following time points:

Before the first administration in the first cycle;

From cycle 2, before the first administration of the test drug in each even-numbered cycle (within -4h);

After completion of all treatment cycles (0-7 days after end of treatment).

About 3mL of blood is collected each time.

10 Efficacy Evaluation

10.1 Tumor evaluation method and time

Serum tumor marker testing and tumor imaging examinations will be performed during the screening period. In addition, investigators may determine whether a whole-body bone scintigraphy is necessary based on the patient's condition. Serum tumor marker testing will be based on the specific type of tumor.

Tumor radiographic evaluates tumor response in all subjects according to RECIST V1.1 criteria.

Screening assessment must include the chest, and entire abdomen, including the pelvic cavity. Except for subjects with nasopharyngeal carcinoma, all subjects with other tumor types should undergo brain imaging examinations during the screening

period. Enhanced brain MRI is the first choice. If the patient has received imaging examination within 28 days prior to the first drug administration and meets the requirements, repeat examination may not be required. For tumor evaluation, enhanced CT or enhanced MRI (CT thickness less than 5mm) is the first choice. If subjects are allergic to CT contrast agent, plain CT or enhanced MRI can be used for examination. For special lesions (such as skin lesions), only clinical examination can be performed (relevant color photos should be archived). Subjects should use the same imaging technique throughout the study. Additional imaging may be performed at any time if disease progression is suspected by investigator during the study. From C1D1, during study treatment: tumor evaluation is performed every 6 weeks ±7 days in the first year of administration and every 12 weeks ±7 days in the second year of administration; Tumor assessment is performed every 12 weeks ±7 days in the first year after the last administration; Tumor assessment is performed every 24 weeks ±7 days in the second year after the last administration. Until the patient starts new antineoplastic therapy, or the patient develops disease progression, or the patient withdraws informed consent, drops out, lost to follow-up, or dies, whichever comes first. The timing of imaging examinations should be consistent with the calendar day and should not be adjusted for delayed or extended treatment cycle.

The sponsor will collect and properly manage patients' imaging data with sensitive information removed (including but not limited to patients' names, addresses, etc.), which will be retained for subsequent exploratory analysis or retrospective analysis.

10.2 Efficacy evaluation criteria

Tumor efficacy evaluation will be classified according to RECIST V1.1 efficacy criteria: CR (complete response), PR (partial response), SD (stable), and PD (progression). Target lesion progression is assessed compared to the minimum tumor size (the minimum sum of lesion diameters previously recorded during study treatment). When there is no progression, baseline tumor sizes obtained prior to initiation of

treatment are compared to assess tumor efficacy (CR, PR, SD).

In order to determine the objective response rate (ORR), once CR or PR is present during follow-up, it needs to be confirmed at the next imaging examination.

If the investigator is in doubt about whether disease progression has occurred, it is recommended that treatment continues until follow-up evaluation is confirmed. If a review scan confirms disease progression, the date of the original scan should be used as the date of disease progression.

In order to confirm "definite progression" of non-target lesions, the non-target disease must have deteriorated substantially at the overall level, so that the increase in overall tumor load is sufficient to cause termination of treatment even in the case of SD or PR in the target lesion. A mild "increase" in the size of one or more non-target lesions is usually insufficient to be considered definite disease progression.

In this study, an independent imaging evaluation institution can be introduced to independently analyze and judge the imaging data of all subjects at each time point in the treatment period according to RECIST V1.1 standards in blind condition, and the IRC evaluation charter can be used in the operation.

11. Adverse Events

11.1. The Definition and Report of Adverse Event

Adverse Event (AE) refers to all adverse medical events occurred after a subject received the investigational drug, manifesting as symptoms, signs or abnormal laboratory tests, without a define causal relationship with the investigational drug.

New conditions or the deterioration of original conditions will be considered as AE. Preexisting stable chronic conditions, which do not worsen during the study period, such as arthritis, will not be regarded as AE. Laboratory abnormalities, clinical symptoms or signs that are clinically significant as determined by the investigator will be considered as AE.

In this study, disease progression (such as tumor enlargement, metastasis, etc.) is not reported as an Adverse Event (AE). Adverse conditions caused by disease progression need to be reported as AEs. For example, if a patient develops pleural effusion due to disease progression with lung metastasis, the term for this event should be "pleural effusion" rather than disease progression. If a patient develops fatal multiple organ failure due to disease progression, "multiple organ failure" should be reported as a Serious Adverse Event (SAE) with a fatal outcome, rather than "fatal disease progression" or "death due to disease progression". If death occurs due to tumor progression without specific complications, the SAE name can be "Disease Progression". During this study, anti-drug antibody positive is not considered as an AE, unless the investigator believes that anti-drug antibody positive is or might be related to clinically significant adverse events, thus the event will be reported as AE.

Collection Route and Time of Adverse event:

All adverse events occurring from the first administration of the study drug until 28 days after the last administration must be completely documented in the subject’s case report form. Serious Adverse Events (SAEs) related to study procedures that occur after signing the Informed Consent Form (ICF) but before the first administration of the study drug must also be collected. Other adverse events that occur during the same period (after signing the ICF but before the first administration of the study drug) will be documented as medical history/concomitant diseases, rather than Adverse Events (AEs).

Each time, the investigator shall ask the subject about the occurrence of adverse event by open-ended questions (e.g., "Has anything change occurred since last visit? "Or" Are there any other questions?). The investigator shall avoid asking the subject in a way that has an impact on the subject.

The investigator will record the onset date, AE description, severity, action taken, event outcome, and evaluate the relationship between the events and the investigational drug.

11.2. Adverse Event Record and Follow-up

All adverse events occurred after the subject received the investigational drug must be recorded completely on the patient's case report form, with support of raw data for the documentation. Detailed description of each case is required, which include the onset date, end date, severity, relationship with the investigational drug, action taken, and outcome. The outcomes of adverse events are defined as follows: Recovered/Cured (no sequelae), Persistent (not recovered/not cured), Improved (recovered or cured with sequelae), Death, Aggravated, and Unknown.

The investigator has the responsibility to provide appropriate medical management to treat all the adverse events. The physical conditions of each subject will be monitored by the investigator or designated personnel throughout the clinical trial. During each visit, symptoms and signs observed by the investigator (or designated personnel) or voluntarily reported by the subject will be recorded.

Adverse event will be followed up for outcome before recovery.

11.3. Criteria for Adverse Event Severity Evaluation

All adverse events occurred during the study should be reported and documented in the original medical history and case report form. Severity is assessed according to NCI-CTCAE V5.0.

Grade 1 -- mild; no symptom or mild symptoms; only with clinical or diagnostic findings; no treatment required.

Grade 2 -- moderate; minimal, local or non-invasive treatment indications; with age-related limitations in instrumental activities of daily life.

Grade 3 - severe or clinically significant, but not immediately life-threatening; with indications for hospitalization or prolonged hospitalization; disabling; with limitations in automatic daily activities.

Grade 4 - life threatening, requiring urgent medical treatment.

Grade 5 - resulting in death.

It is crucial to distinguish between severity of adverse events (AE) and serious adverse events (SAE). An AE with severe intensity is not necessarily considered serious. For example, nausea lasting several hours may be classified as "severe nausea" but will not be regarded as an SAE unless it meets the corresponding criteria. On the other hand, a stroke causing mild disability may be identified as a "mild stroke," but it will be reported as an SAE if it satisfies the SAE criteria. Additionally, an isolated CTCAE Grade 4 laboratory abnormality should not be considered a life-threatening condition, unless the associated clinical signs/symptoms or diagnosis meet the criteria for a life-threatening SAE.

11.4. Determination of Causality between Adverse Events and the Investigational Drug

Investigators should determine whether there is a reasonable association between an adverse event and the study drug based on their understanding of the patient, the patient’s condition before and after the occurrence of the adverse event, and an evaluation of any potential causes. The assessment should be conducted in accordance with the following guidelines:

- Temporal relationship between the occurrence of the event and the start of study drug treatment
- Known correlation between the event and the study drug or similar treatments
- Known correlation between the event and the study disease
- Presence of risk factors in the patient, or concurrent use of medications known to increase the incidence of the event
- Presence of known non-treatment-related factors associated with the occurrence of the event.

The investigator’s judgment on the causal relationship is one of the criteria that directly determines the sponsor’s regulatory expedited reporting of Suspected Unexpected Serious Adverse Reactions (SUSARs). Investigators must provide a

reasonable medical judgment based on existing clinical evidence, referring to information in the Investigator's Brochure (IB) or the study drug's package insert.

The investigator will determine the relationship between an adverse event (AE) and the trial drug into five levels based on their clinical judgment and the following definitions: definitely related, probably related, possibly related, possibly unrelated, and unrelated. The specific judgment criteria are as follows:

- Definitely related: Occurs after administration of the study drug, or there is a reasonable temporal association between the occurrence of the AE and the use of the study drug; the AE is more reasonably explained by the study drug than by other etiologies.
- Probably related: Occurs after administration of the study drug, or there is a reasonable temporal association between the occurrence of the AE and the use of the study drug; the AE is more reasonably explained by the study drug than by other etiologies; and there is a positive dechallenge response (improvement or resolution after drug discontinuation).
- Possibly related: Occurs after administration of the study drug, or there is a reasonable temporal association between the occurrence of the AE and the use of the study drug; however, there are other potential etiologies that could explain the AE; and there is a positive dechallenge response.
- Possibly unrelated: Occurs after administration of the study drug, or there is a certain temporal association between the occurrence of the AE and the use of the study drug (e.g., it may be a delayed side effect); however, there are other reasonable etiologies that could explain the AE; and the dechallenge response is negative or unclear.
- Unrelated: The study drug is not used; or there is no reasonable temporal association between the use of the study drug and the occurrence of the AE; or there is another clear cause for the AE.

The possible causal relationship between AE and the investigational drug and the concomitant medications should be determined by the investigator. In addition to the evaluation of causal relationship between AE and the investigational drug, the investigator shall elaborate the basis for judgment as far as possible. When the severity

of an event worsens or a serious adverse event occurs, the principal investigator or the coordinating investigator shall take the primary responsibility for the judgment of causality, which shall be reflected in the medical records. Relevant medical professionals can be invited for consultation and judgment if necessary.

11.5. Serious Adverse Event

11.5.1. Definition of Serious Adverse Event

Serious adverse event (SAE) refers to an adverse event meets one or more of the following criteria:

1. Resulting in death;
2. Life threatening: refers to that the immediate risk of death of a seriously ill patient, rather than the death may occur when serious development occurs in the future;
3. Resulting in hospitalization or prolongation of hospital stay. It is necessary to clearly confirm that the cause of this condition is an adverse event, rather than hospitalization due to elective surgery, or non-medical reasons.
4. Permanent or severe disability or loss of function;
5. Congenital disease or birth defect.
6. Other important medical events: medical and scientific judgment must be used to determine whether to expedite the reporting of other conditions, for instance, the important medical event may not expose immediately life threatens, death or hospitalization, but medical measures are required to prevent the occurrence of such event, thus the event is also considered as a serious adverse event. For example, critical care in the emergency room or allergic bronchospasm occurred at home, outpatient dyscrasia or convulsions, drug dependence or addiction.

SAE Exemption Instructions

The following specific hospitalizations and prolonged hospital stays do not need to be reported as Serious Adverse Events (SAEs):

1. Hospitalizations for protocol-specified visits during the clinical trial, with no occurrence of new adverse events or exacerbation of existing diseases (e.g., procedures or administration of study drugs as required by the protocol; routine clinical management of CTCAE Grade 1-2 laboratory abnormalities during scheduled visit hospitalizations is not considered a prolonged hospital stay and should be reported as non-serious AEs in accordance with the AE definition).
2. Elective hospitalizations unrelated to the deterioration of adverse events (e.g., elective surgery).
3. Hospitalizations for administrative reasons (e.g., annual routine physical examinations).
4. Hospitalizations for reasons such as medical insurance reimbursement (e.g., routine conditions that do not require hospitalization, such as the common cold or general conditioning).
5. Medical or surgical procedures (e.g., endoscopy, appendectomy): The condition leading to such procedures should be recorded as an AE (e.g., appendicitis).

Unexpected adverse drug reaction refers to the nature, severity, consequence, or frequency of an adverse reaction occurred during the study are different from the expected risks described in the current documents of the investigational drug (e.g., investigator's brochure). The investigator's brochure, as the primary document, provides reference safety information to determine whether an adverse event is expected or unexpected. For example: (1) acute renal failure is listed as an adverse reaction in the investigator's brochure, thus interstitial nephritis occurred during the study should be judged as an unexpected adverse reaction; (2) hepatitis is listed as an adverse reaction in the investigator's brochure, thus acute severe hepatitis occurred during the study should be judged as an unexpected adverse reaction.

Suspected unexpected serious adverse reaction (SUSAR) is an adverse reaction that is not only serious and unexpected, but also suspected drug-related, which shall file rapid report to the health authority in accordance with the Standards and Procedures for Rapid Reporting of Safety Data during Drug Clinical Trials

11.5.2. Reporting and follow-up of serious adverse events

Any SAE that occurred during the study must be fully documented in the patient's case report form. At the same time, the investigator must fill in the Severe Adverse Event Report Form and report to the sponsor (or the contract Research Organization (CRO) appointed by the sponsor), the ethics Committee of the research center (according to requirement of study centers) within 24 hours after learning the SAE. The investigator must record in the Severe Adverse Event Report Form the patient number, the date of SAE occurrence, the date SAE was known, the name of the drug under study, symptom description, severity, duration, measures taken and outcome, and a preliminary assessment of causality.

If the investigator cannot learn about the serious adverse event in a timely manner (for example, the patient was first admitted to another hospital), he/she should report and record the time of first learning about the serious adverse event within 24 hours after learning about it.

For all serious adverse events, it is the investigator's responsibility to follow up and provide information to the sponsor in accordance with the reporting deadlines specified above. In addition, the sponsor may require the investigator to quickly collect specific additional information. This information may be more detailed than that recorded in the Serious Adverse Event Report Form. Normally, this information should include a detailed description of the serious adverse event to allow for a complete medical assessment of the event and an initial independent assessment of the probable cause. In addition, information on other possible causes, such as concomitant medications and concomitant diseases, must be provided. In the event of the death of the patient, the autopsy report, if available, must be submitted to the sponsor or its designated representative as soon as possible. The sponsor will promptly submit individual case safety reports to the national drug regulatory authority and the National Health Commission in accordance with the Standards and Procedures for Expedited Reporting of Safety Data During Clinical Trials of Drugs. Then, the SUSAR report shall be promptly distributed to the Principal Investigators (PIs) of each center, so that the PIs can submit it to the ethics committee and research institution of their respective research centers.

In addition to individual safety reports of unexpected serious adverse reactions, information on other potential serious safety risks identified by the investigator and sponsor should also be reported to the National Drug evaluation Authority as soon as possible, and medical and scientific judgments should be made on each case. In general, this applies to information that has a significant impact on the risk benefit assessment of a drug product, or that may consider a change in drug use, or affect the overall drug development process, for example: (1) for known, serious adverse reactions, the incidence is increased and judged to be clinically important; (2) obvious harm to the exposed population, such as ineffective drugs in the treatment of life-threatening diseases; (3) Significant safety findings (such as carcinogenicity) in recently completed animal tests.

For all serious adverse events (including serious adverse events that are still evolving after the study ends, as well as those occurring within 28 days after the study conclusion), the investigators need to follow-up until fully recovered or stability, or return to baseline, or start a new anti-tumor treatment, death, lost to follow-up, withdraw the informed consent form, and provide detailed follow-up information.

SAE that occurred 28 days after the last administration or after trial withdrawal need to be reported only if the investigator determines that the event is definitively related to the study drug.

11.5.3. Pregnancy

Women of childbearing age can also participate in this study as long as adequate contraceptive measures are adopted. During the screening, the investigator must inform women of childbearing age the importance of contraception, acceptable contraceptive methods and the potential risks of unintended pregnancy during the study. If a woman is suspected to be pregnant during the study, she must notify the investigator immediately. The investigator must promptly carry out pregnancy test for the subject. If the test result is positive, the investigator should file the Pregnancy Report Form provided by the sponsor within 24 hours, and send to the sponsor (or the sponsor's designated CRO), the ethics committee of the study site as soon as possible. The sponsor will decide whether the female subject shall continue the study based on her pregnancy conditions. The investigator should follow up the pregnancy event and

provide outcomes to the sponsor (or the sponsor's designated CRO) in a timely manner. Pregnancy itself is not an adverse event, but the event should be evaluated to determine whether the event shall be reported as an adverse event. Abortion, whether is accidental, therapeutic or spontaneous, shall be recorded and reported as SAE.

The above-mentioned consultation and follow-ups shall be provided to the sex partner of the pregnant subject.

The investigator will collect all the information related to pregnancy from conceiving to delivery, using forms provided by the sponsor (or the sponsor's designated CRO).

12. Statistical Analysis

12.1. Determination of sample size

The sample size of this study was not based on statistical assumptions, but on the number of DLT cases observed at each dose level and the number of dose groups required to determine MTD, which is estimated to be about 28 cases in total.

The maximum sample size is estimated in accordance with the traditional 3+3 dose escalation design: 1 subject will be enrolled in one dose group for accelerated titration. Under different circumstances, there will be a maximum of 9 dose groups excluding the accelerated titration dose group, resulting in a total of 55 subjects (calculation: $1 + 6\times9 = 55$).

12.2. Analysis Set

12.2.1. Definition of analysis set

The definition of analysis sets and criteria for inclusion/exclusion of subjects from each analysis set will be detailed in the SAP.

This study includes 8 data analysis sets: the Screened Subjects Set, Enrolled Subjects Set, Safety Analysis Set, Dose Escalation Analysis Set, Blood Drug Concentration Analysis Set, Pharmacokinetic Analysis Set, Immunogenicity Analysis

Set, and Efficacy Analysis Set. Their definitions are as follows:

- **Screened Subjects Set**: All patients who have signed the informed consent form.
- **Enrolled Subjects Set**: All patients who have passed the screening.
- **Safety Analysis Set**: All patients who have passed the screening and received the study drug at least once.

This set will be used for the analysis of safety data and other baseline data (e.g., demographic characteristics).

- **Dose Escalation Analysis Set**: Includes patients who experienced Dose-Limiting Toxicity (DLT) during the dose escalation phase, as well as patients who did not experience DLT and completed the first cycle of study drug administration.
- **Blood Drug Concentration Analysis Set**: All patients who have passed the screening, received the study drug at least once, and have at least one valid blood drug concentration data.
- **Pharmacokinetic (PK) Analysis Set**: All patients who have passed the screening, received the study drug at least once, and have at least one calculable PK parameter.
- **Immunogenicity Analysis Set**: All patients who have passed the screening, received the study drug at least once, and had at least one collection of immunogenicity data (excluding baseline) during the trial.
- **Efficacy Analysis Set**: All patients in the expanded enrollment phase who have passed the screening, received the study drug at least once, and have baseline tumor assessment data and post-treatment efficacy evaluation data. Patients in the dose escalation phase may also be included in the Efficacy Analysis Set if they meet the efficacy analysis criteria of the expanded enrollment phase and receive the same dose as in the expanded enrollment phase

12.2.2. Determination of analysis set

Patients in the Safety Analysis Set will be analyzed based on the actual dose administered at the initiation of the trial. However, some lists (e.g., adverse event lists) will specify the actual treatment dose received at the time of the adverse event (e.g., dose reduction, administration interruption, etc.).

Patients in the Dose Escalation Analysis Set will be analyzed based on the actual dose administered at the initiation of the trial. However, some lists (e.g., adverse event lists) will specify the actual treatment dose received at the time of the adverse event (e.g., dose reduction, administration interruption, etc.).

The Pharmacokinetic (PK) Analysis Set will be determined by at least the head of PK analysis, investigators, and study statisticians prior to database lock. Adverse events or protocol deviations may result in patients being excluded from the PK Analysis Set, and these cases will be discussed in data review meetings.

For the Efficacy Analysis Set, whether patients from the dose escalation phase can be included in the analysis set will be determined based on their actual medication status, and such determination will be made prior to database lock.

12.3. Statistical Methods

12.3.1. General Statistical Considerations

Descriptive statistics of continuous variables include number of cases, mean, standard deviation, median, quartile, minimum and maximum. Descriptive statistics were used for classification variables by frequency and percentage. Detailed statistical analysis strategies and procedures will be described in a separate Statistical analysis plan (SAP).

Descriptive statistical analysis was conducted for safety outcomes, efficacy outcomes, PK parameters, immunogenicity, biomarker measurements, etc., without statistical tests. These data were presented in summary charts or lists.

After the start of the trial, if there are changes to the primary study outcomes and important secondary study outcomes, or changes to the corresponding statistical analysis methods, the protocol and statistical analysis plan shall be revised and the final version shall be obtained before the database is locked. After the database lock, additional exploratory analyses not covered by the protocol and statistical analysis plan need to be described in detail in the statistical analysis report (SAR) and clinical trial report (CSR).

12.3.2. Demographic Analysis

The safety analysis data set will be used for baseline data analysis. Patient characteristics include baseline history and disease characteristics, and each patient will be listed by trial phase and dose group. Descriptive statistical analyses will be performed to summarize the demographic and baseline characteristics of the study population and each dose group.

12.3.3. Dose-Limiting Toxicity Evaluation

Summarize the number and percentage of patients with DLT by dosing schedule and dose level. The analysis will be based on the dose escalation analysis set.

12.3.4. Safety Analysis

With the exception of DLT, all safety analyses are based on safety analysis set.

Safety data are not statistically tested.

All adverse events will be coded and summarized using MedDRA. Adverse event toxicity will be classified and summarized according to NCI-CTCAE V5.0. The number of cases and incidence were summarized by systematic organ classification (SOC) and preferred terms.

Changes in other safety outcomes, such as vital signs and laboratory tests, from baseline, if applicable, will also be summarized by dose regimens and dose level. In the case of qualitative laboratory tests, the frequency and percentage of test results were calculated at each point in time of visit.

Outliers from laboratory tests and vital signs will be identified in the list and, where applicable, a list of clinically significant abnormal test values will also be provided (if applicable).

The results of the physical examination are presented only in the list.

Concomitant medications will be coded and summarized using the WHO Drug Dictionary (WHODD).

12.3.5. Pharmacokinetic Analysis

Based on the plasma drug concentration-time data of each patient measured in the experiment, plot the drug-time curve for each individual patient and the mean drug-time curve for each dose group (including the semi-logarithmic plot), adopts the non-compartmental model analysis to estimate the pharmacokinetic parameters, obtained the main pharmacokinetic parameters of drugs, to fully reflect the characteristics of the drug distribution in human body. For samples at concentrations below the lower limit, PK parameters should be calculated as zero before C_{max} and Not detectable (ND) after C_{max}.

PK samples will be taken at the specified time points in the study flow chart to analyze and describe the concentration-time characteristics of the patients and calculate the following pharmacokinetic parameters of the patients:

Total exposure (area under the curve AUC_{0-T} and/or AUC_{0-INF})

Peak concentration (C_{max}) and peak time (T_{max})

Half-life $T_{1/2}$

Valley concentration (C_{trough})

Clearance rate (CL)

Volume of distribution (Vd)

And other pharmacokinetic parameters

At each sampling time, pharmacokinetic parameters will be statistically described as follows: N, arithmetic mean, standard deviation and Coefficient of Variation (CV), geometric mean, median, quartile, minimum and maximum.

12.3.6. Efficacy Analysis

The efficacy analysis will use the efficacy analysis set to conduct statistical descriptions of objective response rate (ORR), disease control rate (DCR), duration of response (DOR), progression-free survival (PFS), and overall survival (OS). The ORR and its 95% confidence interval in each dose group will be calculated using the Clopper-Pearson exact probability method. The best overall response will be summarized. The

median DOR, PFS, OS and their 95% confidence intervals for each dose group will be estimated using the Kaplan-Meier method, respectively.

Objective response rate (ORR): All patients will be assessed individually according to RECIST criteria 1.1. ORR is defined as the proportion of patients whose best response is complete or partial response to the total number of evaluable patients. Patients for whom lesion and tumor response assessment has not been performed will be classified as non-evaluable patients and excluded from the calculation.

Disease control rate (DCR): defined as the number of cases with remission and disease stabilization after treatment as a percentage of the total evaluable cases.

Duration of remission (DOR): Defined as the time from the first recorded remission to the time of disease progression or death. The results of tumor evaluation are analyzed by Kaplan-Meier method.

Progression-free survival (PFS): the time between the start of study medication and the first observation of disease progression (based on imaging), calculated using the actual time for tumor evaluation. If patients died from other causes before disease progression is observed, the number of days from the beginning of study medication to death will be calculated. For patients with no disease progression or death at the time of analysis, progression-free survival will be dated at the time of the last tumor evaluation and processed as censored data.

Overall survival (OS): the time between the start of study medication and death. If patients are alive at the end of the study, overall survival will be calculated based on the last contact date or the end date of the study, whichever occurs first. The last contact date is the last recorded date in the database.

DOR, PFS and OS will be analyzed based on the tumor evaluation results of investigators. Kaplan-Meier method will be used to estimate the median DOR, PFS, OS and 95% confidence interval of each dose group, and K-M curves will be drawn.

13. Deal with abnormal situation during study

13.1. Risk assessment and risk management plan

The test site must be equipped with necessary medical rescue equipment, first-aid

drugs and emergency measures. Set up emergency medical emergency team when necessary, deal with emergency medical emergency and unexpected disaster according to relevant standard operation procedures.

Patients must be enrolled strictly in accordance with the inclusion/exclusion criteria and the time intervals specified both within each dose group and between different dose groups.

Closely observe possible adverse events, especially unexpected adverse events, timely analyze and communicate, and fill in the adverse event observation log.

The same patient can only be enrolled in one dose group, and dose escalation is not allowed in the same patient.

Establish liaison procedures with the hospital intensive care unit to coordinate patient transfer and care.

Establish communication and communication between investigators, laboratories and sponsors to ensure timely communication and treatment of possible adverse events.

Patients with adverse reactions during treatment may continue to participate in the study according to the suspension or termination of the treatment plan (Section 3.4.5), or discontinue the study treatment if the withdrawal criteria are met.

13.1.1. Risks associated with study drug

Based on the preclinical safety study of BL-M07D1 and the clinical trial results of DS-8201 (a HER2-ADC of the same class), the potential adverse events (AEs) of this study are predicted.

[redacted]

In the phase III clinical trial of DS-8201 (a HER2-ADC of the same class) in patients with HER2-positive breast cancer, the most common grade 3 or higher drug-

related treatment-emergent adverse events (TEAEs) were: neutropenia (19.1%), thrombocytopenia (7.0%), leukopenia (6.6%), nausea (6.6%), anemia (5.8%), fatigue (5.1%), vomiting (1.6%), increased alanine transaminase (ALT, 1.6%), decreased appetite (1.2%), increased aspartate transaminase (AST, 0.8%), diarrhea (0.4%), and alopecia (0.4%). Overall, 10.5% of patients were confirmed to have interstitial lung disease (ILD) or treatment-related pneumonia. Most ILD events (9.7%) were primarily low-grade: grade 1 (2.7%) or grade 2 (7.0%); 2 cases of grade 3 events (0.8%) were reported. No grade 4 or grade 5 ILD or pneumonia events occurred [12].

13.1.2. Overall plan for safety management

For adverse reactions occurring during the study, it is recommended to follow the following procedures for review or follow-up, and consult a specialist doctor when necessary:

[redacted]

[redacted]

[redacted]

[redacted]

[redacted]

[redacted]

[redacted]

[redacted]	
[redacted]	[redacted]
[redacted]	[redacted]
[redacted]	[redacted]
[redacted]	[redacted]

[redacted]

[redacted]

[redacted]

[redacted]

[redacted]

[redacted]

[redacted]

[redacted]

[redacted]

[redacted]

[redacted]

[redacted]

[redacted]

[redacted]

[redacted]

[redacted]

[redacted]

[redacted]

[redacted]

[redacted]

[redacted]

[redacted]

[redacted]

[redacted]

[redacted]

[redacted]

[redacted]

[redacted]

[redacted]

[redacted]

[redacted]

13.2. Protocol Deviation

Refer to the Protocol Deviation Manual.

14. Protocol Approval and Amendment

Prior to the start of this clinical study, the protocol and other relevant documents of the clinical study should be submitted to the Independent Ethics Committee (IEC) /competent authorities for approval as per local legal requirements. The sponsor and investigator must ensure that all ethical and legal requirements are met before the first subject is enrolled in this clinical study.

During the study, this study protocol must be strictly followed. If a protocol modification is required, a written amendment shall be used. Before implementation (if applicable), the amendment shall be issued by the responsible personnel and approved by the Institutional Ethics Committee (IEC)/competent authority.

Administrative changes do not require formal amendments but still need to be described in the comprehensive clinical study report. All amendments with appropriate instructions shall be distributed to all protocol recipients.

15. Data Management

15.1. Requirements for the investigator to fill in data

An Electronic Data Capture (EDC) system will be used in this study. The study data (excluding pharmacokinetic, immunogenic and exploratory study data) will be entered into eCRF by the investigator or authorized staff of the study site. Before launching the study site or entering the data, the investigator and the authorized staff of the study site shall be properly trained, and appropriate information security measures will be taken.

The eCRF should be completed during or after the visit period as soon as possible, and be timely updated to ensure that it can reflect the latest information of the subjects participating in this study. In order to avoid differences in evaluation results between different evaluators, it should be ensured as far as possible that the baseline and all subsequent efficacy and safety evaluations for the same subject are completed by the same person. The investigator must audit the data to ensure the accuracy and correctness of all the data entered into the eCRF. In case some evaluations are missing or some information is unavailable, inapplicable or unknown in the study process, the investigator should record them in the eCRF. The investigator shall electronically sign the audited data.

Unless otherwise specified, the eCRF can only be used as a form for data collection, not as original data. The original documents are all the records that are used by the investigator or the study site and related to the subjects and that can prove the existence of the subjects, their inclusion/exclusion criteria and their participation in this study, including laboratory records, ECG results, subject folders, etc.

The investigator is responsible for maintaining all original documents, and ensuring to provide them to the clinical research associate (CRA) for monitoring in each visit. In addition, regardless of the length of their participation in this study, the investigator must submit an eCRF for each subject participating in this study. The study

number and subject number of all the supporting documents submitted together with the eCRF (such as laboratory records and study site records) should be carefully verified, and, to protect the privacy of the subjects, all the personal private information (including subject name) should be deleted or made unidentifiable.

When the study data are entered into eCRF, the system will automatically add the identity of data entry person through the ID of logged-in user. The investigator uses its electronic signature record to prove that a record has been audited, and thus guarantees the data accuracy of this record. Electronic signature will be completed via the investigator's user ID and password, and the system will also automatically attach the date and time of signature. If the data in eCRF need to be changed, it shall be done as per the workflow defined by EDC system. All changes and their reasons shall be recorded in the audit trail.

15.2. Data Monitoring

The CRA assigned by the Sponsor (or the contract research organization (CRO) appointed by the Sponsor) will review original medical records and eCRF, evaluate and ensure their completeness and consistency, while the CRA will compare the eCRF with the original and other relevant documents to ensure the integrity and consistency of critical data.

15.3. Database Establishment and Data Entry

The investigator or its authorized personnel will be responsible for the entry, correction and changes of all data, while the CRA has no such authority. The data in the eCRF is submitted to the data server, and any changes to the data will be recorded in the audit trail, that is, the reason for changes, operator name, time and date of changes will be recorded. The roles and permissions of the study site personnel responsible for data entry will be determined in advance. If there is a data query, the CRA or data management personnel will issue the query in the EDC system, and the study site

personnel will be responsible for answering the query. The EDC system will record the audit trail of the query, including the investigator's name, time and date.

15.4. Protection of Confidential Data

All clinical study results and documents will be kept confidential. The investigator and his study team members must not disclose such information without the prior approval of the Sponsor.

The identities of subjects participating in this study must not be disclosed. In eCRF and other documents, the subject can only be represented by subject number, initials or date of birth, while the subject's name must not appear. The documents identifying the subjects (e.g. signed information for subjects and informed consent forms) must be kept confidential by the investigator.

15.5. Data Lock and Handover

A data review meeting shall be held, where the principal investigator, investigator, testing facility, sponsor, manager of statistical analysis, Person in charge of data management and CRA shall jointly determine the population of data set, and then the database will be locked by the data management personnel.

When locked, the database is handed over to statistical analysts for statistical analysis as per the requirements of the SAP. After the completion of statistical analysis, statistical analysts shall prepare the statistical analysis report, and the principal investigator shall be responsible for the clinical study report.

16. Quality Assurance

16.1. Quality Assurance of Clinical Study Process

16.1.1. Monitoring

This study will be monitored according to China GCP and ICH-GCP. The Sponsor or the contract research organization (CRO) appointed by the Sponsor shall conduct clinical monitoring on this study. The clinical research associate (CRA) shall conduct the monitoring in accordance with the SOP, and have the same rights and responsibilities as the monitor of the Sponsor. The CRA should maintain regular communication with the investigator and the Sponsor.

The CRA shall perform monitoring visits in accordance with the requirements of all relevant laws and regulations. The CRA shall evaluate the capabilities of the study site and report the related issues of facilities, technical equipment, or the investigator to the Sponsor. Regular monitoring visits to the study site will be performed from the time subjects are enrolled. After each visit to the study site, the CRA shall submit a written report to the Sponsor. During the study, the CRA will be responsible for monitoring whether the written ICFs of all subjects have been obtained, and whether the data records are correct and complete. Meanwhile, the CRA will also compare the data entered into eCRF with the original data and inform the investigator of any errors or omissions. The CRA will also monitor the compliance of the study site with the protocol, the supply and preservation of investigational drugs.

16.1.2. Data Verification

In this study, the CRA is required to have direct access to the original data for verification, which is realized by verifying the data in the subject's eCRF and his original data. The data verification process is an important part of quality assurance of the study, in which transcription errors and omissions can be corrected.

16.1.3. Audit and Inspection

The representative of the Sponsor, regulatory departments and the IEC may visit the center for audit or inspection, including the verification of original data.

16.1.4. Personnel Training

The principal investigator will keep a list of authorized assignments and training records for all study-related personnel. It is ensured that all such personnel have been properly trained in relation to the study and that any information related to the conduct of the study has been passed on to the relevant personnel.

16.2. Quality Assurance of Sample Testing Process

Each sample shall be labeled as per laboratory requirements.

The pharmacokinetic analysis and testing of this study can be located in a third-party testing facility as appropriate.

The collected biological samples will be transported at a low temperature to a third-party testing facility for storage by a designated professional express company.

17. Ethical Criterion and Informed Consent Form

17.1. Ethical Criterion

This protocol shall be reviewed and approved by the Medical Ethics Committee before implementation. During the review, the Sponsor and investigator shall provide the Ethics Committee with documents such as “Clinical study approvals for the drug", "Certificate of analysis for the drug", "Sample of informed consent form", "Study protocol", "Investigator's brochure" and "qualification certificates of principal investigator” and so on. All communications with the Ethics Committee should be kept in the investigator's folder.

Any changes of the protocol during the study must also need to approved by the Ethics Committee and can be implemented only after the approval is obtained.

17.2. Informed Consent Form

The informed consent form must comply with ICH-GCP, China GCP, Chinese regulations and legal requirements.

The investigator must obtain the signed informed consent form from each subject before any study-related activities can be started. The informed consent form used in this study and any changes in the course of this study must be approved by the Ethics Committee before use. The investigator shall keep the informed consent form signed by each subject. The informed consent form shall be retained as a clinical study document for future reference.

18. Research Reports

18.1. Tolerability and Preliminary Efficacy Evaluation Report

The main contents are as follows:

- Safe dose range for phase I clinical trials；
- Recommended dose and rationale for Phase II clinical studies；
- Doses without adverse reactions；
- Doses in which adverse reactions occurred；
- Doses in which mild adverse reactions occurred；
- Analysis of adverse reactions: For any adverse reactions that occur, an analysis shall be conducted to assess the relationship between reaction severity and dose dependence; regarding the timeline of adverse reactions, attention shall be paid to observing whether the reaction gradually worsens or resolves spontaneously, and a comparative analysis of the patient’s condition before and after the reaction shall be performed.

- Efficacy analysis;
- Summary by the principal investigator of the Phase I trial.

18.2. Pharmacokinetic Study Report

The main contents are as follows:

- Protocol implementation;
- Patient safety evaluation;
- Establishment of detection methods;
- Patient biologic sample concentrations and calculated pharmacokinetic parameters;
- Drug accumulation evaluation;
- Summary by the principal investigator of the Phase I trial.

19. Data Retention

During the implementation of the study and after the study is completed, the study site/Sponsor shall file the necessary documents to the study files in accordance with the requirements of China GCP.

In addition, these documents will be kept until

- at least 5 years after study completion or early discontinuation of the study;
- or at least 5 years after the final approval of marketing application in China, and until there are no pending or under review marketing applications in China;
- or at least 5 years after the formal termination of clinical development project of the investigational drug; whichever is longer.

The Sponsor shall timely inform the investigator of any documents that need no further preservation. These documents must not be destroyed/moved before the Sponsor is informed and its consent is obtained.

20. Liability and Insurance

The insurance coverage shall be set in accordance with the GCP guidelines and relevant local insurance laws. The subjects will be compensated in the event of death or any damage to their health or living conditions as a result of their participation in this study.

The insurance policy shall be preserved in the master folder of the study.

Reference

[1] Simon, R., Rubinstein, L., Arbuck, S. G., Christian, M. C., Freidlin, B., & Collins, J. (1997). Accelerated titration designs for phase I clinical trials in oncology. Journal of the National Cancer Institute, 89(15), 1138-1147.

[2] Meizi Liu, Sue-Jane Wang & Yuan Ji (2019). The i3+3 design for phase I clinical trials, Journal of Biopharmaceutical Statistics, DOI: 10.1080/10543406.2019.1636811.

[3] Gamis A S, Alonzo T A, Meshinchi S, et al. Gemtuzumab Ozogamicin in Children and Adolescents With De Novo Acute Myeloid Leukemia Improves Event-Free Survival by Reducing Relapse Risk: Results from the Randomized Phase III Children's Oncology Group Trial AAML0531. Journal of Clinical Oncology, 2014, 32(27):3021-3032.

[4] Damelin Marc, Zhong Wenyan, Myers Jeremy et al. Evolving Strategies for Target Selection for Antibody-Drug Conjugates. Pharm Res, 2015, 32: 3494-507.

[5] Tipton Thomas R W, Roghanian Ali, Oldham Robert J, et al. Antigenic modulation limits the effector cell mechanisms employed by type I anti-CD20 monoclonal antibodies. Blood, 2015, 125: 1901-9.

[6] Donaghy Heather. Effects of antibody, drug, and linker on the preclinical and clinical toxicities of antibody-drug conjugates. MAbs, 2016, 8: 659-71.

[7] Alley Stephen C, Benjamin Dennis R, Jeffrey Scott C et al. Contribution of linker stability to the activities of anticancer immunoconjugates. Bioconjug Chem, 2008, 19: 759-65.

[8] Nagayama Aiko, Ellisen Leif W, Chabner Bruce, et al. Antibody-Drug Conjugates for the Treatment of Solid Tumors: Clinical Experience and Latest Developments. Target Oncol, 2017, 12: 719-739.

[9] Shefet-Carasso L, Benhar I. Antibody-targeted drugs and drug resistance challenges and solutions. Drug Resist Updat. 2015,18:36-46.

[10] Li Fu, Emmerton Kim K, Jonas Mechthild et al. Intracellular Released Payload Influences Potency and Bystander-Killing Effects of Antibody-Drug Conjugates in Preclinical Models.

Cancer Res, 2016, 76: 2710-9.

[11] 姜文奇, 巴一, 冯继锋,等. 肿瘤药物治疗相关恶心呕吐防治中国专家共识(2019 年版)[J]. 中国医学前沿杂志（电子版）, 2019,11(11):16-26.

[12] 中国临床肿瘤学会抗肿瘤药物安全管理专家委员会, 中国临床肿瘤学会肿瘤支持与康复治疗专家委员会. 抗肿瘤治疗引起急性口腔黏膜炎的诊断和防治专家共识[J]. 临床肿瘤学杂志, 2021.

[13] Lacouture, M. E , Sibaud, et al. Prevention and management of dermatological toxicities related to anticancer agents: ESMO Clinical Practice Guidelines. Ann Oncol.2021,32(2):157-170.

[14] 秦叔逵,马军.中国临床肿瘤学会（CSCO）肿瘤放化疗相关中性粒细胞减少症规范化管理指南（2021）[J].临床肿瘤学杂志, 2021,26(07):638-648.

[15] 2022 年 CSCO 肿瘤治疗所致血小板减少诊疗指南.

[16] 史艳侠,邢镨元,张俊,沈波.中国肿瘤化疗相关性血小板减少症专家诊疗共识（2019 版）[J].中国肿瘤临床,2019,46(18):923-929.

[17] 辉瑞制药有限公司, 盐酸伊立替康注射液说明书, Version No: 20210311

[18] Bossi P, Antonuzzo A, Cherny NI, Rosengarten O, Pernot S, Trippa F, Schuler U, Snegovoy A, Jordan K, Ripamonti CI; ESMO Guidelines Committee. Diarrhoea in adult cancer patients: ESMO Clinical Practice Guidelines. Ann Oncol. 2018 Oct 1;29(Suppl 4):iv126-iv142.

[19] Conte P, Ascierto P A, Patelli G, et al. Drug-induced interstitial lung disease during cancer therapies: expert opinion on diagnosis and treatment[J]. ESMO open, 2022, 7(2): 100404.

[20] Swain S M, Nishino M, Lancaster L H, et al. Multidisciplinary Clinical Guidance on Trastuzumab Deruxtecan (T-DXd)–Related Interstitial Lung Disease—Focus on Proactive Monitoring, Diagnosis, and Management[J]. Cancer Treatment Reviews, 2022: 102378.
